# Potential exposure risk of global timber production, carbon stocking, and biodiversity to forest pests

**DOI:** 10.1038/s41467-026-78728-x

**Published:** 2026-10-15

**Authors:** Yuxin Qiao, Jianquan Liu, David P. Edwards

**Affiliations:** 1 State Key Laboratory of Herbage Innovation and Grassland Agro-Ecosystem, College of Ecology, Lanzhou University, Lanzhou, China; 2 Department of Plant Sciences and Centre for Global Wood Security, University of Cambridge, Cambridge, UK; 3 Conservation Research Institute, University of Cambridge, Cambridge, UK

**Keywords:** Conservation biology, Forestry, Forest ecology, Ecosystem ecology

## Abstract

Pests pose escalating threats to forest ecosystem services globally, yet the patterns of forest vulnerability to these threats remain unclear. Leveraging over 7 million occurrence records of 1875 pest species, we identify northwestern Europe and eastern North America as global epicenters of forest-pest richness, dominated by insect fauna. Potential risk zones where timber production, carbon-rich forests, or diverse tree communities are exposed to high pest richness within and outside host native ranges cover 3.5% and 1.6% of forest globally, respectively. Forests in regions with stable temperatures, adequate precipitation, and high timber import volumes support high ecosystem services but also favor elevated pest richness, while forest fragmentation amplifies vulnerability to pests. Our findings provide a spatial blueprint for global pest management and underscore the urgent need for protecting forest health.

## Abstract (web summary)

Forest pests threaten timber production, carbon storage and biodiversity worldwide. This study identifies exposure hotspots in northwestern Europe and eastern North America, shaped by climate, trade and forest fragmentation.

## Introduction

Forests are vital for global ecosystem services, including carbon storage, biodiversity, timber production, and soil and water provisioning^1,2^. However, these services are increasingly jeopardized by outbreaks of pests—any living organism that is injurious to plants or plant products^3^. In forest ecosystems, these pests include insects, pathogenic microbes, parasitic plants, and any other harmful organisms that cause physiological and structural damage to trees^3^. The threat is escalating globally, with over 7000 alien insect species documented and new introductions occurring continuously^4^. The proliferation of insect pests is further aided by the expansion of their plant hosts^5^, which act as both food sources and corridors^6^. Concurrently, pests within host native ranges are increasing their range and impact due to climate change and global trade^7,8^, leading to severe damage^1,2^.

The escalating global threat from forest pests reflects the combined effects of climate change, landscape modification, and international trade. Climate warming and more frequent extreme droughts weaken tree resistance, extend pest overwintering ranges, and enhance pesticide resistance^9,10^, exacerbating pest spread. In Europe, the overlap between forest and pest habitats is projected to increase by 51% under medium-to-high emissions scenarios^11^. These climatic shifts are compounded by human-mediated dispersal. Global trade is a primary vector for such introductions, as exemplified, for example, by the flight-limited European spongy moth, which causes extensive damage in Canada—its spread accelerated by the transport of infested wood materials^12^. Once introduced, the establishment and rapid colonization of pests are further facilitated by landscape modification because of human disturbances to natural environments. Habitat degradation and fragmentation^13^, combined with the uniform genetic composition and closely spaced trees of monoculture plantations, provide easily accessible food for pests, leading to rapid colonization^14^.

These intersecting drivers can result in severe ecological and economic consequences, ranging from tree mortality and growth inhibition to species extinction^15^. For example, the introduction of the pathogen causing chestnut blight disease from Asia to the USA, which led to the functional extinction of the American chestnut, killed an estimated four billion trees^16^. Similarly, the box tree moth, introduced via the plant trade, has rapidly devastated box tree populations across Europe^17^. Such tree loss impairs critical ecosystem functions like rainfall interception and soil stability, exacerbating climate change impacts^18,19^. The economic costs are therefore substantial, including lost timber and increased management expenses^2^, and the degradation of forest carbon stocks threatens climate mitigation goals, including those of the Paris Agreement^20^.

Mapping global pest distributions and their overlap with key ecosystem services is essential for sustainable forest management. We assemble a global dataset of over seven million occurrence records of 1875 forest-pest taxa (Supplementary Fig. 1) to: (1) map the global patterns of pest richness; (2) for critical forest ecosystem services, evaluate the potential risk zones for forest pests occurring within and outside their host native ranges (hereafter within and outside host native ranges; see “Methods”; Supplementary Fig. 2); and (3) elucidate the environmental, landscape, and socioeconomic drivers of these potential risk zones. To provide a structured framework for this assessment, we adopt the IPCC Sixth Assessment Report (AR6) risk framework, which defines risk as hazard, exposure, and vulnerability^21^. Here, we conceptualize pest richness as the hazard component (i.e., the potential of pests to cause harm) and the spatial distributions of timber production, carbon storage, and tree biodiversity as the exposure component (i.e., the presence of valuable forest assets that may be affected). Accordingly, our analysis identifies potential risk zones (hazard-exposure overlap zones), characterized by the co-occurrence of high pest richness and three critical forest ecosystem services.

## Results

### Richness pattern of forest pests

**Fig. 1:**
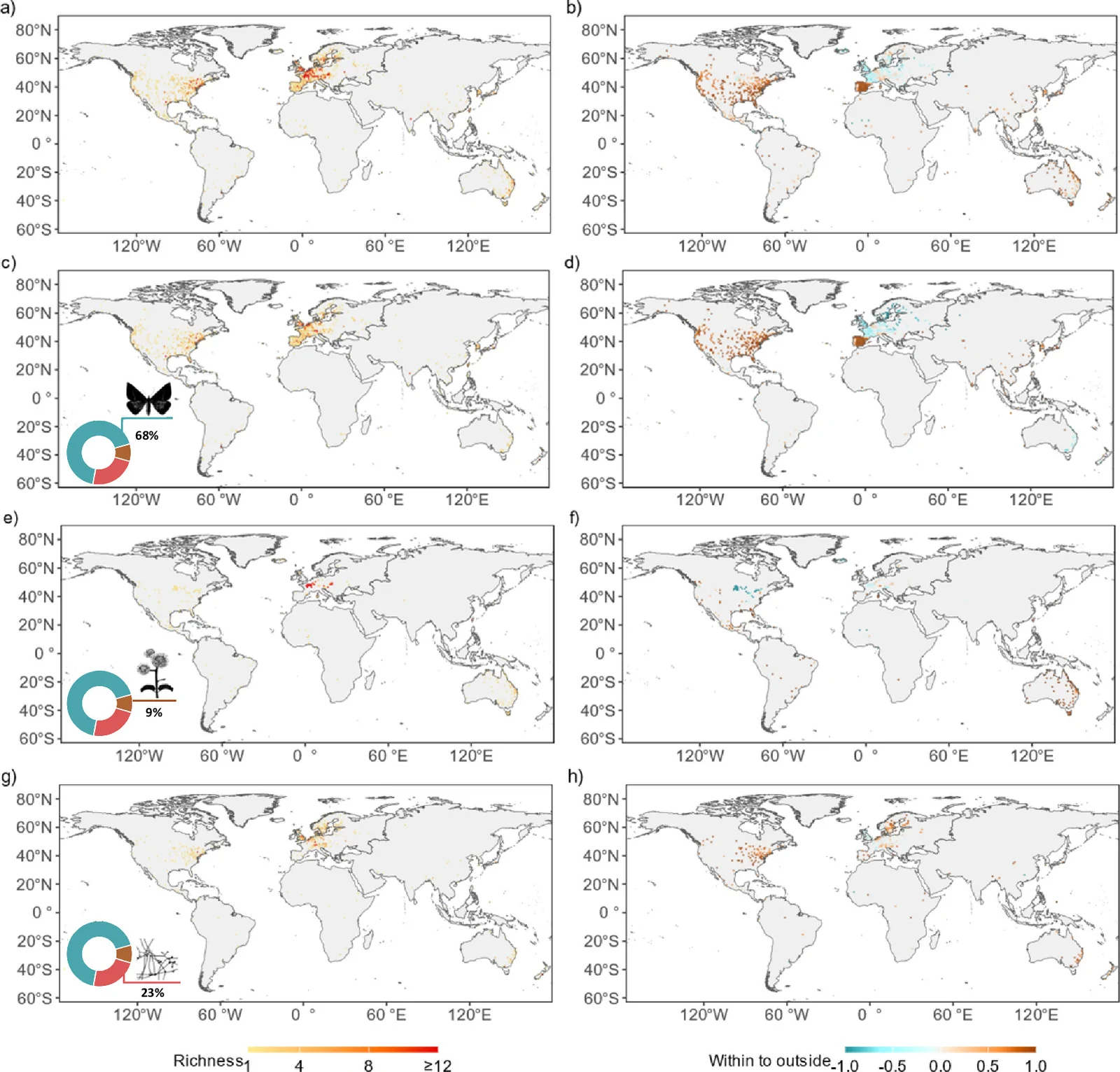
Global spatial pattern of forest pest richness. All forest pests with **a** overall richness and **b** scaled richness ranging from −1 to +1, comparing the relative difference in pest richness within and outside the native ranges of host trees (positive values indicate higher pest richness within host native ranges). **c**, **d** Fauna**. e**, **f** Flora**. g**, **h** Pathogenic forest pests. Circle plots in the bottom-left indicate the proportion of each pest group among all forest pests. The color bar indicates the species richness of each pixel, with warmer colors representing higher values. To improve display, all maps have been resampled to 1° resolution.

Forest pests are primarily distributed in Europe, as well as the United States of America (USA), Mexico, southern South America, and eastern Australia (Fig. 1a), with the epicenter of pest richness (top 5% of cells) concentrated in northwestern Europe and in smaller areas of eastern USA (Supplementary Fig. 3a). Forest pests within host native ranges were concentrated in southwest Europe and southeast USA (Fig. 1b), whereas those outside host native ranges were mainly distributed in northwest to central Europe (Fig. 1b).

Overall, 68% of forest pests were fauna (*N* = 1384) with over 92% of records Insecta (*N* = 1285; Extended Data 1), covering 4.6% of global land forest area, in particular within epicenters in northwestern Europe and eastern USA (Supplementary Fig. 3b), plus occurrences in eastern Australia (Fig. 1c). Fauna pests within host native ranges were concentrated in southwestern Europe and southeastern USA, whereas pests outside host native ranges were mainly distributed across northwestern to central Europe (Fig. 1d). This dominance and geographical distribution likely reflects both environmental permissiveness and intensive anthropogenic dispersal^22^. Global trade networks disproportionately link Europe and North America, creating frequent opportunities for accidental transport and establishment of forest pests through introduction pathways including infested wood packaging, firewood, and the international trade in live plants and nursery stock^23,24^. The similarity of temperate broadleaf forest flora and extensive urban-forest interfaces in these regions also facilitates establishment after introduction^25^. Moreover, longer-term historical processes, such as colonial-era host plant exchanges and accidental introduction to the Americas and Europe associated with warfare^26,27^, have created persistent biogeographic linkages and dispersal routes that continue to shape present-day pest distributions. High richness of forest pests in Europe may also reflect historically developed surveillance infrastructure, as European countries have maintained comprehensive forest health monitoring networks for several decades, supported by dense scientific survey and cross-border collaboration^28,29^.

Only 9% of forest pests were flora (*N* = 179) with Magnoliopsida (*N* = 148; Extended Data 1) representing over 83% records, covering 1.2% of global forest area, in particular within epicenters in northwestern Europe (Supplementary Fig. 3c), plus occurrences in eastern USA (Fig. 1e). Flora pests within host native ranges were concentrated in central Europe and western USA, whereas pests outside host native ranges were mainly distributed across western Europe and eastern USA (Fig. 1f). Pathogenic forest pests comprised 23% of the dataset (*N* = 475) with Pucciniomycetes (*N* = 85), Sordariomycetes (*N* = 78), and Dothideomycetes (*N* = 72) together accounting for 51% of records, covering 1.9% of global forest area, especially within epicenters in northwestern and central Europe (Supplementary Fig. 3d), plus occurrences in eastern USA (Fig. 1g). Pathogenic pests within host native ranges were concentrated in northwestern Europe, whereas pests outside host native ranges were mainly distributed across northeastern Europe and eastern USA (Fig. 1h). More localized hotspots may reflect stronger host specificity and narrower climatic niches (and less climatic niche plasticity) of flora and pathogenic pests than generalist insect herbivores^30,31^.

Surveillance and reporting biases^32^ in occurrence datasets (taxonomic and geographic related) can exaggerate apparent concentration in well-sampled temperate regions relative to unevenly sampled tropical regions (e.g., South America, Africa). To assess the robustness of these patterns, we computed scaled standardized report rates (SRRs) following Fabre et al.^33^. Our SRR-scaled analysis revealed that several western European countries retained high pest richness after standardization, with Belgium, the UK, France and Germany showing positive scaled SRR values (Supplementary Fig. 4). This pattern was accompanied by high occurrence-record density, consistent with established forest health monitoring networks supported by dense scientific survey and cross-border collaboration in Europe^28,34^. In contrast, the high raw richness observed in eastern North America was less consistently retained after standardization. The USA showed only positive scaled SRR values for fauna (0.05) and pathogen pests (0.15), but a negative value for flora pests (−0.28) (Supplementary Fig. 4). These results indicate that the high raw richness in eastern North America is not consistently retained after the standardization of forest pest records, and is likely influenced by high reporting intensity for some pest groups, especially those with negative scaled SRR values.

To support the interpretation of the SRR analysis, we constructed a null model incorporating gross national income (GNI), forest area, and timber import volume (*R*^2^ = 0.718). After accounting for these predictors, northwestern European countries still exhibited positive residuals (Supplementary Fig. 5), indicating that observed pest richness in these regions remained higher than expected given these predictors. Together with high occurrence records and positive scaled SRR, this suggests that high raw richness in western Europe was robust after standardization. In tropical regions, low raw pest richness was often accompanied by low occurrence records or insufficient data for reliable SRR estimation, indicating that limited recording may constrain the assessment of forest pest richness. Although we focused on pest occurrence and retained unique points for each grid cell to reduce uneven observation effect^35^, biological characteristics of tropical systems may still limit detectability of pests. Many tropical herbivores exhibit relatively high host specificity compared with temperate species^36^, and in hyper-diverse forests where conspecific trees are spatially dispersed, host-associated insects occur at lower local densities, making detection through conventional surveys more challenging^37^.

### Ecosystem-service exposure to forest pests

**Fig. 2:**
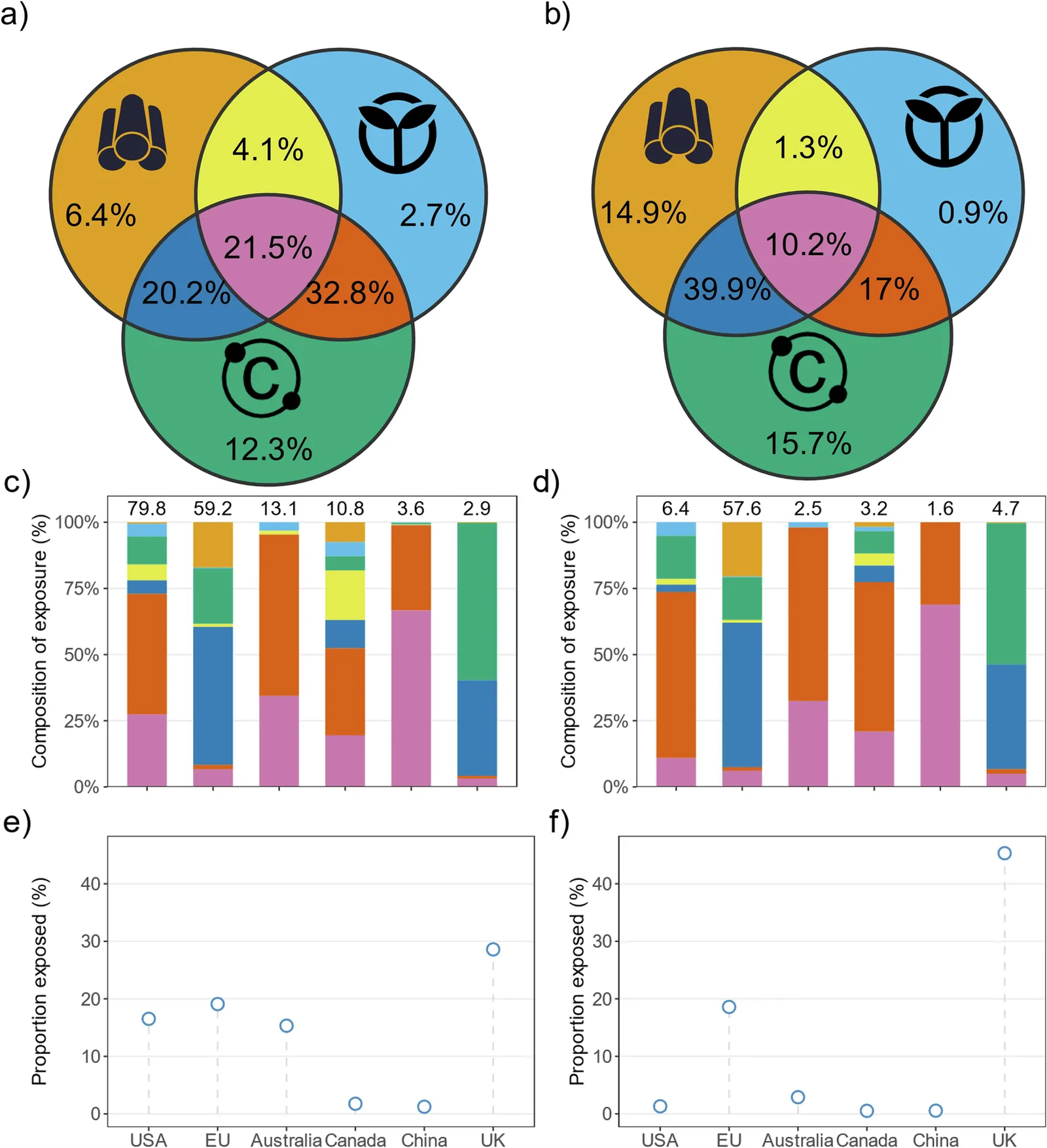
Global potential risk zones exposed to forest pests across different ecosystem services. Percentages of total exposed land area of timber production, forest tree diversity, and potential carbon stock across different types of potential risk zones exposed to forest pests **a** within and **b** outside host native ranges. The orange, blue, and green colors represent potential risk zones exposed to forest pests across a single ecosystem service; the yellow, dark orange, and deep blue represent potential risk zones exposed to forest pests across pairs of ecosystem services; and the pink represents the intersection of all three ecosystem services. The bar charts represent the composition of exposed area for the countries/regions with the largest exposed zones **c** within and **d** outside host native ranges, and the numbers above the bars indicate the total area exposed. The scatter point charts show the proportion of potential risk zones relative to total forest area across countries/regions **e** within and **f** outside host native ranges.

We overlaid pest richness maps with three critical forest ecosystem services—timber-producing area, forest tree diversity, and potential carbon stock—to identify potential risk zones (Fig. 2). We found potential risk zones exposed to forest pests within and outside host native ranges covered 3.5 and 1.6% of global forest area, respectively (Fig. 2a, b). For pests within host native ranges, these zones encompassed 102 million ha (Mha) of timber-producing areas, 124 Mha of forest areas characterized by high tree diversity (averaging 52 tree species per ha), and 71 Mha of high carbon stock forests (averaging 26 ton per ha), with co-occurring high tree diversity and carbon stock accounting for 32.8% of the potential risk zones (Fig. 2a). This pattern corresponds with previous studies that attribute increased pest richness to forests that offer a wider array of host resources^38^, higher levels of primary productivity supporting larger consumer populations^39^, and more complex forest structure that provides abundant niches and refuges for herbivore pests^40^.

For pests outside host native ranges, potential risk zones spanned 60.4 Mha of timber-producing areas, 30.1 Mha of forest areas characterized by high forest tree diversity (averaging 52 species per ha), and 27.7 Mha of potential carbon stock (averaging 26 ton per ha), with 39.9% of these potential risk showing co-occurrence of timber-producing and high carbon stock areas (Fig. 2b). This reflects the role of timber-producing areas as hubs of international wood trade and movement of infested packaging materials, which facilitate long-distance dispersal of pest introductions^41,42^. The prevalence of monoculture in these areas also provides dense, uniform host environments, further contributing to the establishment and spread of alien pests^43^.

Locations where high forest pest richness within host native ranges coincide with timber-producing areas, high tree diversity, and potential carbon stock were primarily distributed in northwestern Europe and eastern USA (Supplementary Fig. 6a, c, e), whereas the areas with high forest pest richness outside host native ranges were mainly distributed across Europe (Supplementary Fig. 6b, d, f).

Nationally, we identified the top six regions (hereafter, main exposure regions) with the largest potential risk zones, including the USA, European Union (EU), Australia, Canada, China, and United Kingdom (UK) (Fig. 2c, d). The USA contained the largest areas of potential risk zones (79.8 Mha) exposed to forest pests within host native ranges (Fig. 2c), with substantial potential carbon stocks (0.19 million tons) and nearly half of this area (Supplementary Fig. 7a; 36.4 Mha; 45%) comprising locations where high potential carbon stock and forest tree diversity co-occurred (Fig. 2c). This highlights critical vulnerability to USA forestry, particularly as climate change amplifies the impact of pest species within host native ranges. Warmer temperatures can accelerate insect metabolism and development, reduce winter mortality, and expand the geographical ranges of pests into previously inhospitable northern latitudes^44^.

For pests outside host native ranges, the EU contained the largest area of potential risk zones (57.6 Mha; Fig. 2d), most of which was characterized by timber production (47.4 Mha) and potential carbon stocks (0.17 million ton; Supplementary Fig. 7d, f), and more than half of which (31.5 Mha; 54%) comprised zones where timber production and high potential carbon stock co-occurred (Fig. 2d). The UK had the highest proportion of potential risk zones to national forest areas for pests within (28.6%; Fig. 2e) and outside (45.3%; Fig. 2d) host native ranges, respectively. Pronounced vulnerability of ecosystem services in European forests to pests^45^ reflects both centuries of alien species import via well-connected trade hubs^46^ combined with timber production characterized by high-density, intensive monocultures and structurally simplified plantations^14,47^.

**Fig. 3:**
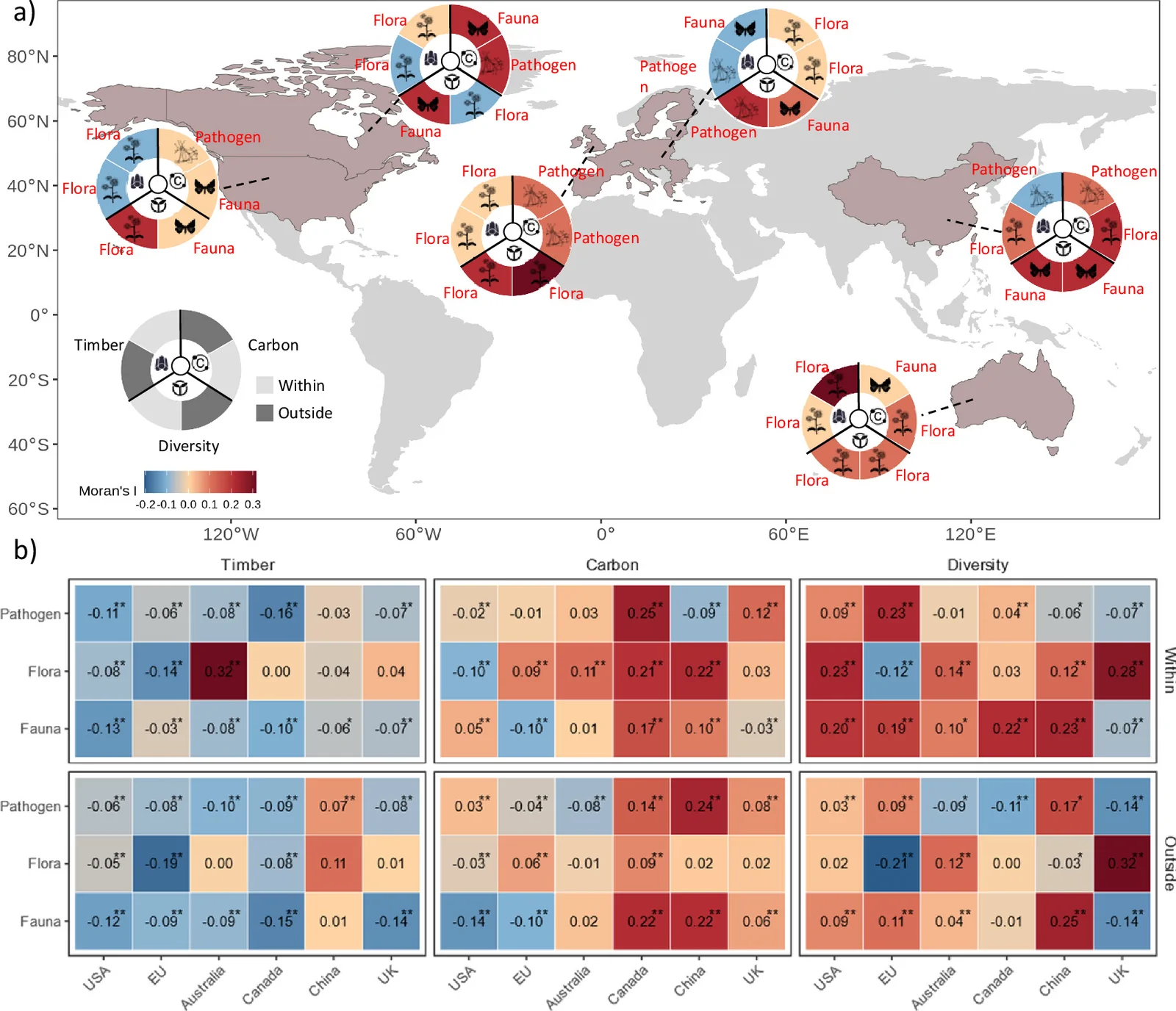
Spatial correlation between forest pest functional groups and ecosystem services across main exposure regions. **a** The most spatially correlated forest pest functional groups with ecosystem services across the main exposure regions. Circular diagrams indicate the pest-service combinations with the highest global bivariate Moran’s *I* values. The legend with gray color (light gray, pest within host native range; dark gray, pest outside host native range) indicates the three ecosystem services. **b** Heatmaps of global bivariate Moran’s *I* values for all combinations of pest functional groups and ecosystem services. A warmer color represents a clustered pattern for forest pests and ecosystem services, and a cooler color represents a dispersed pattern. A value tending to 0 represents a random pattern. Asterisks denote significance levels (*p* < 0.05*, *p* < 0.01**) based on two-sided permutation tests with 499 permutations. Full statistical results, including 95% confidence intervals and exact *P* values, are provided in Supplementary Table 1.

Taxonomic pest groups often display different host-selection tendencies and environmental tolerances^43^. Using global bivariate Moran’s *I*, we identify which groups (flora, fauna, and pathogenic pests) posed the highest exposure risk to ecosystem services within and outside host native ranges (Fig. 3). Flora forest pest richness showed the strongest spatial associations with exposure to the three ecosystem services (Fig. 3a). A negative spatial association between flora pest richness and timber-producing areas across most regions (Moran’s *I* < 0; Fig. 3b, Supplementary Table 1) likely reflects simplified habitat heterogeneity that cannot support a wide array of host-specific floral pests (e.g., parasitic plants)^48^ although specialist pests can spread rapidly and cause large-scale mortality even when pest richness is low^49^. For example, dwarf mistletoes (*Arceuthobium spp*.) are highly specialized obligate hemiparasites endemic to western North America that target commercially valuable conifer forests (e.g., lodgepole pine, *Pinus contorta*^50^), causing 1.1 million m^3^ timber loss per year^51^. In contrast to regions where timber-producing areas show negative spatial associations with flora pest richness, timber-producing areas in China (Moran’s *I* = 0.11, *p* = 0.14) and Australia (Moran’s *I* = 0.32, *p* < 0.01) exhibit a positive spatial correlation with higher flora pest richness. This pattern likely reflects mixed timber plantations, sometimes including native tree species, proximity to natural forests, plus exploitation in natural forests (in Australia)^52,53^.

Regions characterized by both high potential carbon stock and forest tree diversity also provided a favorable foundation for flora pests. Our spatial analysis revealed that flora pest richness had the strongest positive correlation with forest tree diversity in the USA (Moran’s *I* = 0.23 for within host native range; *p* < 0.01), Australia (Moran’s *I* = 0.14 and 0.12 for within and outside host native range, respectively; *p* < 0.01), and the UK (Moran’s *I* = 0.28 and 0.32 for within and outside host native range, respectively; *p* < 0.01). Correlations with potential carbon stock were strongest in the EU (Moran’s *I* = 0.09 and 0.06 for within and outside host native range, respectively; *p* < 0.01), Australia (Moran’s *I* = 0.11 for within host native range; *p* < 0.01) and China (Moran’s *I* = 0.22 for within host native range; *p* < 0.01) (Fig. 3; full statistical results, including 95% confidence intervals, are provided in Supplementary Table 1). These patterns can be explained by host-parasite interaction and plant defense theory. For example, parasitic species (e.g., Cuscuta spp.) need established permanent haustorial connections with host xylem and phloem^54^. Consequently, their occurrence is tightly coupled to areas with high host density and, where abundant, nutrient fluxes facilitate haustorium development and growth^55^. Moreover, according to the resource availability hypothesis, trees in resource‑rich environments tend to invest less in structural and chemical defenses and more in rapid growth^56^. If they lose leaves or other tissues to parasites, it takes them less time to recover. Therefore, fast-growing trees in high carbon stock forests may be more susceptible to parasite attachment and penetration^57,58^.

In addition to flora pests, regions with high forest tree diversity showed significant positive associations with fauna pest richness in most regions except for the UK (Moran’s *I* = −0.07 and −0.14 for pests within and outside host native range, respectively; *p* < 0.01). Regions with potential carbon stock had the strongest correlation with pathogenic pest richness in the UK (Moran’s *I* = 0.12 and 0.08 for pests within and outside host native range, respectively; *p* < 0.01) (Fig. 3). This pattern can be explained by both host‑specific and generalist dynamics. Specialist herbivores encounter their preferred hosts more frequently in diverse forests, enhancing colonization success^59^. At broader scales, a previous study reports a unimodal relationship between tree diversity and pest richness^38^. Using data from 2,098 U.S. counties spanning from near-monocultures to more than 60 species, Guo et al.^38^ showed that pest richness increases with tree diversity (both host and non-host) at low and moderate levels but declines at very high diversity. Our study spans a narrower diversity gradient (from 1 to 40 tree species), yielding a consistent positive spatial correlation between tree and fauna pest richness (Supplementary Table 1). Furthermore, we focus on the pests with confirmed host associations, which is fully consistent with pest richness increasing with host tree diversity only^38^.

While our macroecological analysis reveals a positive spatial association between tree diversity and pest species richness, this pattern supports the well-documented dilution effect, where increased host diversity reduces the transmission efficiency or outbreak intensity of specific pests^60,61^. The dilution effect is typically observed at local scales (e.g., plot-level experiments), where high tree diversity lowers the density of susceptible hosts, thereby inhibiting the outbreak intensity of individual pest species^61^. In contrast, our study focuses on regional species richness across broad spatial extents. At this macro scale, higher tree diversity provides a wider variety of niches and host resources, facilitating the accumulation of a larger regional pool of host-specific herbivores and pathogens^62^. Consequently, a diverse forest can harbor a high richness of pest species (many specialists at low density) while simultaneously exhibiting low damage severity due to the dilution effect. Furthermore, the observed pest richness in hyper-diverse tropical regions may be underestimated due to monitoring limitations and biological characteristics^36^, with many tropical herbivores exhibiting host specificity and low local abundance^36^. Such biological traits, combined with uneven surveillance efforts in tropical countries, make forest pests harder to detect, potentially generating observation bias^37^.

### Factors related to ecosystem-service exposure to forest pests

**Fig. 4:**
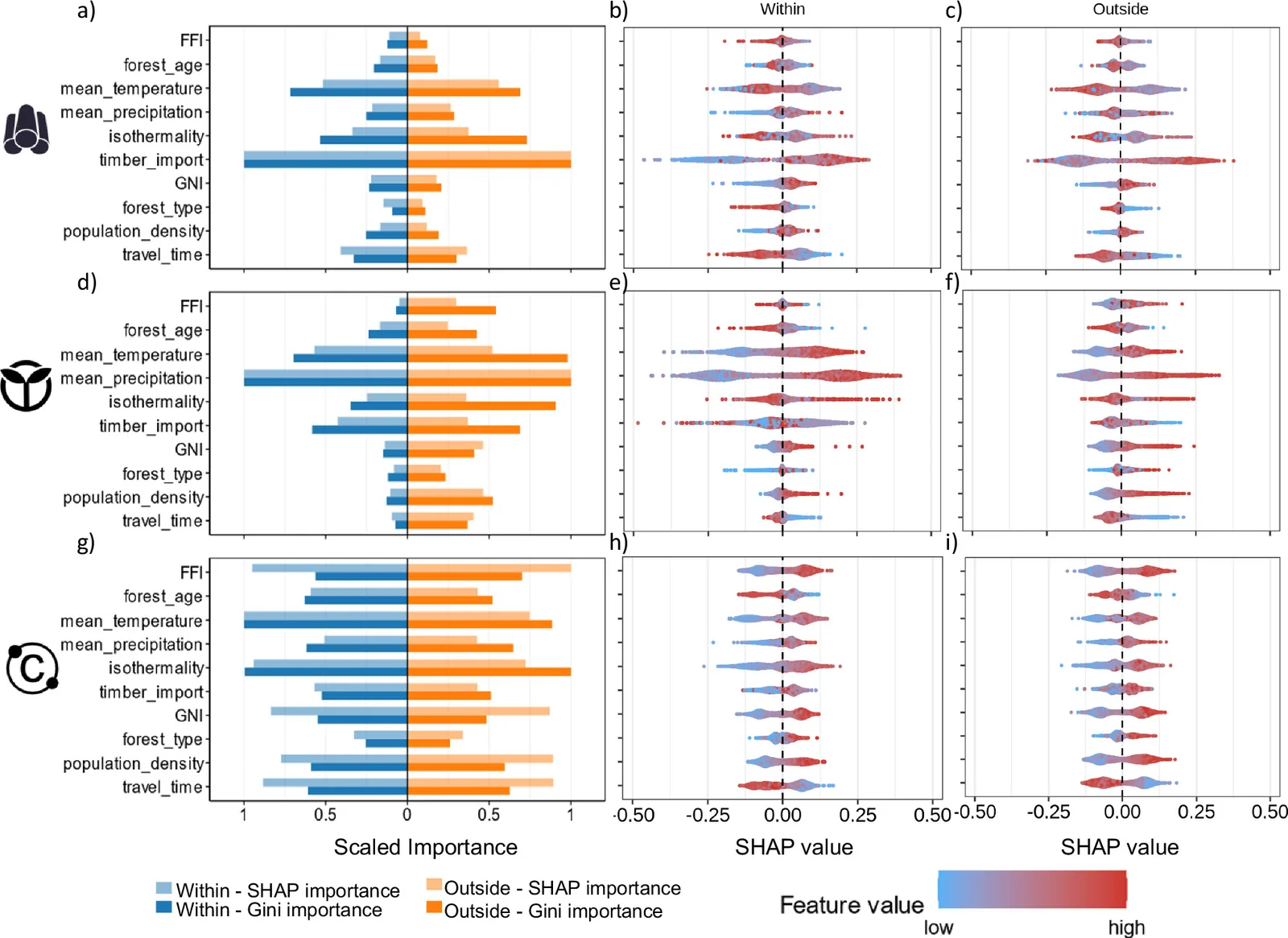
Factors influencing high-risk zones exposed to forest pests among different ecosystem services. The importance of each feature based on Gini coefficients and the mean absolute value of SHAP for potential risk zones of **a** timber-producing areas, **d** forest tree diversity, and **g** potential carbon stock exposed to forest pests within and outside host native ranges. Distribution of SHAP values based on the random forest model among **b**, **c** timber-producing area, **e**, **f** forest tree diversity, and **h**, **i** potential carbon stock within and outside host native ranges of forest pests, respectively. Each point represents the SHAP value for an individual observation, and the color gradient (blue to red) indicates low to high feature values.

To separately quantify the drivers of forest pest within and outside host native ranges, we used an interpretable machine-learning framework (random forest with SHapley Additive exPlanations, SHAP) to quantify the relative importance and impact direction of predictors for shaping potential risk zones for ecosystem service (Fig. 4). Using spatial block cross-validation to control for spatial autocorrelation of predictors, our model achieved mean overall Kappa scores of 0.93 ± 0.04 (sd) and 0.86 ± 0.08 (sd) across the three ecosystem services for forest pests within and outside host native ranges, respectively.

Our results demonstrate that timber import volume, climatic suitability, and forest fragmentation index (FFI) strongly shape the spatial patterns of potential risk zones exposed to forest pests, but that their relative importance differs between pest geographic ranges and ecosystem service type. For potential risk zones in timber-producing areas, both Gini importance and mean absolute SHAP importance indicate that timber import volume ranked the top predictor (Fig. 4a). This suggests that trade-driven propagule pressure is the primary determinant of exposure risk, as larger timber import volumes increase the probability of pest introduction and subsequent spread into timber-producing regions^63^. The strong influence of timber import volume further highlights the role of international wood trade in reshaping pest invasion risk, with areas receiving greater import flows facing substantially higher potential exposure^7,8^. In regions exposed to pests within host native ranges, SHAP values were predominantly positive, indicating that higher timber import volumes substantially increase exposure risk (Supplementary Fig. 8f). This pattern likely reflects the role of timber trade as a major pathway for pest transport, whereby larger import volumes increase the likelihood that infested wood or associated materials introduce pests into timber-producing regions and facilitate their subsequent spread through trade networks^8^. In regions exposed to pests outside host native ranges, SHAP values are largely negative. This suggests that ecological filtering^64^, particularly host limitation, is more important than trade-driven propagule pressure. Although international timber trade can transport pests across regions, the absence or low availability of suitable hosts constrains their establishment, thereby weakening the contribution of timber import volume to predicted exposure risk^65^.

Mean precipitation was the most important factor driving potential risk zones of forest tree diversity (Fig. 4d). Wetter conditions commonly support higher tree species richness and more complex stand structure, which in turn determines the local habitat heterogeneity and promotes phytopathogen transmission^66^. Furthermore, drought stress compromises tree defenses and often coincides with heatwaves, forming a compound drought-heatwave stress that greatly amplifies the severity of pest outbreaks^66^. These two opposing pathways both shape the distribution of potential risk zones for forest tree diversity, resulting in a nonlinear relationship between precipitation and pest exposure risk (Fig. 4e, f; Supplementary Fig. 9d).

For potential risk zones of carbon stock, isothermality, and FFI ranked as the top predictors (Fig. 4g). Temperature largely controls fauna pest physiology and phenology, such as survival rates and emergence timing, which affect voltinism and overwintering mortality^67^. A higher isothermality value usually indicates that an area has a relatively stable temperature, such as a maritime climate in Europe, contributing to phenological synchronization between pest emergence and the susceptible period of trees (e.g., bud burst and cambial activity)^68^. However, the distributions of SHAP values for isothermality are centered symmetrically around zero, indicating spatially heterogeneous and a nonlinear effect of temperature on potential risk zones (Fig. 4b, c; Supplementary Fig. 10e). Such patterns likely reflect species-specific thermal optima and adaptive capacities, with different species and local climate combinations producing opposite effects on exposure risk across locations^69,70^. Forest fragmentation leads to the creation of extensive forest-edge areas^71^, characterized by altered microclimates and stand structures^71^, which are highly favorable for the population development of many forest pests^72^, including non-native beetles in managed European forests^73^, while simultaneously imposing physiological stress on trees and reducing their resistance to attack^74^. Moreover, fragmented landscapes disrupt the habitats of natural pest predators that depend on large, contiguous forest areas^75^, exposing edges to elevated pest threats^76^.

## Discussion

Our study highlights that potential risk zones, where economically important forestry services coincide with high pest richness, should be prioritized for monitoring and rapid response efforts. While these zones are spatially extensive, they offer a valuable global blueprint for guiding resource allocation and regional-scale management. Policies to strengthen pest outbreak management should be prioritized for the main exposure regions identified in this study (e.g., the USA, Europe, and China). These regions are major trade hubs, reinforcing that phytosanitary measures are critical to preventing invasive pests^77^. This includes implementing stricter import controls, enhancing biosecurity at ports and logistics hubs, and mandating the treatment of wood packaging^78^.

Our separation of pests within and outside host native ranges underscores how exposure maps can inform distinct management priorities. For pests occurring within host native ranges, exposure risk is strongly associated with timber import volume, highlighting the role of trade pathways in facilitating pest transport and redistribution among forest regions. In these cases, management should prioritize pathway management, including strengthened inspection of traded wood products, improved biosecurity along trade networks, and enhanced surveillance in major import hubs^63,79^. For pests occurring outside host native ranges, exposure risk is more constrained by ecological filtering, particularly host availability^64^, suggesting that management strategies should focus on monitoring areas where suitable hosts are present or expanding, and on strengthening ecosystem resilience to reduce the likelihood of pest establishment^80^.

Our analysis quantifies pest richness as a measure of hazard, rather than pest abundance or outbreak intensity. While abundance and outbreak severity more directly determine realized damage, globally consistent data on these metrics remain unavailable across taxa and regions^81^. Richness-based patterns therefore provide a practical first-order indicator for prioritizing surveillance at broad spatial scales. In this context, areas with higher pest richness may require greater monitoring attention because they contain a larger pool of taxa from which outbreaks may emerge, particularly where ecological regulation is weakened by host introduction, fragmentation, or climatic stress.

Management strategies should also be tailored to the risk of specific ecosystem services and corresponding key environmental drivers^73^. For instance, the FFI was the most important factor for shaping potential risk zones of potential carbon stocks (Fig. 4g). Between 51 and 67% of global forests became more fragmented from 2000 to 2020^71^, suggesting that the vulnerability of forest carbon storage to pest outbreaks is escalating. To ensure that increasing demand for forest services (e.g., timber, carbon storage) is met under a warming climate^82^, reducing forest fragmentation and restoring forests will be essential. This can be achieved through measures such as establishing ecological corridors, limiting clear-cut size or adopting selective logging to reduce edge creation, protecting existing core forests, and restoring degraded forest lands^83^. Such actions would decrease the susceptibility of high-carbon stock patches, especially when combined with predator-friendly management for pest detection and control^76^. Furthermore, while previous studies have focused on fauna pests (e.g., insects)^14,59,67^, surveillance should also specifically prioritize flora pests, which showed the strongest positive spatial correlation with high-value forest services in the main exposure regions.

Given the multi-scale and heterogeneous nature of pest emergence and spread, a coordinated combination of traditional and advanced tools is essential. Practical approaches include targeted field surveys, the use of unmanned aerial vehicles or hyperspectral remote sensing for broad-scale monitoring, and molecular surveillance techniques like eDNA to facilitate early detection^84^. Building robust information systems and predictive models also necessitates widespread international cooperation. For effective identification and response, surveillance data that can be integrated with predictive niche models to forecast risks must follow common data standards (e.g., consistent field definitions, taxonomic nomenclature, and metadata reporting) and be shared on open-access platforms (e.g., Global Biodiversity Information Facility (GBIF), national pest databases). Such standardization is critical because predictive models require harmonized inputs across regions and databases, and early-warning systems can only aggregate data effectively when all contributors use compatible formats (e.g., Darwin Core standards)^85^. Incorporating artificial intelligence into pest-detection workflows can further automate and improve taxonomic identification and outbreak detection^86^, increasing the speed and accuracy of responses.

## Methods

### Forest pest data collection

We compiled pest species data from the Global Invasive and Alien Species and their Traits (GIATAR) database, which synthesizes global records on alien taxa^87^. The GIATAR integrates multiple authoritative sources, including the European and Mediterranean Plant Protection Organization (EPPO)^34^, the Centre for Agriculture and Bioscience International (CABI)^88^, Standardising and Integrating Alien Species (SInAS)^89^, and Delivering Alien Invasive Species Inventories for Europe (DAISIE)^90^. The GIATAR dataset encompasses 46,666 alien taxa across 249 countries, spanning seven biological kingdoms: Animalia, Bacteria, Chromista, Fungi, Plantae, Protista and Viruses, and provides metadata on biological characteristics (for example, environmental impacts, habitat and host species), spread pathways and geographic ranges.

We identified forest pests using four complementary steps (Supplementary Fig. 1), following the Food and Agriculture Organization (FAO) definition of organisms that cause harm to woody plants, forest ecosystems or derived timber products^3^. This definition includes arthropods (for example, insects and mites), microbial pathogens (for example, fungi and bacteria), parasitic plants (for example, mistletoe), nematodes and vertebrates (for example, rodents). Firstly, we extracted GIATAR records with documented ecological impacts (more than 40 impact categories, including forest productivity, biodiversity and cultural impacts) and retained taxa with unclear forest-related descriptors (for example, “forest production,” “native flora”). Secondly, we selected taxa whose habitat fields explicitly contained forest descriptors. Thirdly, to mitigate data limitations in impact or habitat documentation, we queried the taxonomic information of these hosts of pest species through their scientific names on the GBIF, then retained only those records where the host organism was exclusively a tree species. Fourthly, to account for potential ecological benefits, we excluded pest species with documented positive impacts on forest ecosystems through their impact summary information in GIATAR. Overall, we collected 2038 forest pests, such as Animalia, Plantae, Fungi, Bacteria, Chromista, Viruses, Protozoa (see Extended Data 1), and classified them into three functional groups including fauna, flora and pathogenic pests.

### Geographic ranges of forest pests

To characterize pest distributions, we followed the framework of Gougherty & Davies^91^ and distinguished between pests occurring within and outside their host native ranges. This host-centered classification reflects the ecological dependence of forest pests on their hosts, as pest establishment and spread are strongly mediated by host availability and host-pest interactions^92^. We first compiled host associations for 2038 forest pests and standardized host names using the “taxize” package^93^. Host geographic ranges were then compiled from Global Tree Search^94^, the Global Naturalized Alien Flora database^95^, CABI^88^, and the World Checklist of Vascular Plants^96^. We integrated pest occurrence records with host geographic ranges to determine whether the pest occurred within or outside the native range of its host tree(s). Overall, we obtained usable geographic range information for 1875 forest pests.

### Forest ecosystem services data

To better capture spatial precise distribution of timber production, we employed the data from Curtis et al.^97^, which provides a global analysis of forest loss drivers from 2001 to 2015 using satellite imagery and machine learning classification at 10 km resolution. Using 4985 high-resolution training samples from Google Earth imagery, the dataset used a decision-tree model to categorize forest disturbances into five primary drivers, such as commodity-driven deforestation, shifting agriculture, forestry, wildfires, and urbanization. We only included grid cells associated with forestry activities as areas of timber production. To further capture the influence of wood production trade on forest pest exposure, we included timber import volume data, which were obtained from the Food and Agriculture Organization (FAO) FAOSTAT database^98^ in the driver analysis.

Global tree diversity data were obtained from the high-resolution (0.025° × 0.025°) forest inventory database published by Liang et al.^99^. This dataset integrates standardized measurements from 1.3 million sample plots across 110 countries, covering 97.5% of global forest ecoregions, encompassing about 55 million individual trees representing more than 32,000 species. Plot-level species richness (α-diversity) was normalized to a 1-ha area using rarefaction-based random forest models, accounting for sampling design heterogeneity.

The carbon stock data were derived from Mo et al.^100^, providing an integrated global assessment of global potential carbon stock. The data combined 1,089,026 ground-sourced forest inventory plots data from the Global Forest Biodiversity Initiative^101^ with three satellite-derived aboveground biomass products, including the European Space Agency Climate Change Initiative biomass map^102^, the harmonized global biomass carbon density map^103^ and the global potential biomass map^104^.

### Other data

To characterize the climate in terms of temperature and precipitation, 19 bioclimatic variables (Bioclim: BIO1-19) were downloaded from WorldClim V2.1 at 5-arc resolution^105^. These variables characterize both annual averages and seasonal extremes, providing a comprehensive profile of the climatic environment. Anthropogenic covariates included global travel time to urban centers^106^, GNI^107^, and human population density^108^. For travel time, we used raster pixels that estimate minutes to the nearest urban center. Urban centers were selected as settlements with populations of at least 5000 and no more than 110,000,000.

To quantify biotic drivers of forest vulnerability to pests, we incorporated FFI^74^, forest type^109^, and forest age distributions^110^ as landscape variables. These metrics capture biomechanical pathways through which forest configuration mediates outbreak risks. FFI changes local microclimate and dispersal corridors, facilitating pest colonization^13^, while forest type and age influence host composition and susceptibility to pest outbreaks^111,112^. Forest type was derived from the Moderate-resolution Imaging Spectroradiometer (MODIS) land cover product (MCD12Q1), which provides 500-m resolution land cover classifications. We filtered the forest grid cells following the global vegetation classification scheme of the International Geosphere Biosphere Programme, including evergreen needleleaf, evergreen broadleaf, deciduous needleleaf, deciduous broadleaf, and mixed forest. To calculate the proportion of potential risk zones relative to total forest area for each country, we divided the total area of potential risk zones by the total forest cover within that country^113^.

### Statistical analysis

Based on the scientific names of forest pests (Supplementary Fig. 1), we downloaded more than seven million occurrence data of each pest from the GBIF dataset spanning 1970–2022. The occurrence records included the coordinates (the geographic latitude and longitude records), taxonomic information (e.g., the genus name matched to this occurrence), countryCode (the 2-letter country code of the country, following ISO-3166-1 standards), eventDate (the date-time at which the occurrence was recorded), and coordinateUncertaintyInMeters (the horizontal distance from the given coordinate of the occurrence record as intentional obfuscation to protect sensitive species or private lands). To reduce spatial sampling bias and avoid multiple records representing the same local occurrence, we retained only one unique observation point for each species within each 10-km grid cell^114^. Correspondingly, we removed records with observation distances greater than 10 km via the “coordinateUncertaintyInMeters” field to reduce observation uncertainty. We categorized all forest pests into three groups based on their biological characteristics: fauna (animals), flora (plants), and pathogen pests (disease-causing organisms, such as fungi and bacteria). To compare relative richness within regions, we quantified the standardized difference in pest richness between pest within and outside host native ranges scaled to range from −1 (relatively higher richness outside host ranges) to +1 (relatively higher richness within host ranges), calculated as:

1 $${{\mathrm{Scaled}}}\,{{\mathrm{richness}}}=\frac{{R}_{{\mathrm{within}}}-{R}_{{\mathrm{outside}}}}{{R}_{{\mathrm{within}}}+{R}_{{\mathrm{outside}}}}$$

To evaluate whether raw pest-richness patterns were robust to uneven recording intensity, we calculated an SRR following the framework of Fabre et al.^33^. For each pest type (fauna, flora, and pathogen) and country, the observation value was defined as the species richness of that type. Reference value was defined as the summed species richness of the remaining two forest pest groups in the same country. The SRR was calculated as the ratio of observation value to reference value and weighted by the global expected frequency of each pest type (i.e., the proportion of global richness belonging to that pest type relative to other pest groups). The SRR values were log10-transformed, so that positive values indicate that a given pest type accounts for a higher richness in a country than expected from the global average, whereas negative values indicate that this pest group accounts for a lower richness than expected (Supplementary Fig. 4). Because SRR values can be unstable in countries with very few records, we retained SRR values only for countries with at least 10 records for the pest type being assessed before mapping the results.

To support the interpretation of the SRR analysis, we further constructed a linear model relating pest richness to economic capacity, trade pressure, and host resource availability following the approach of Scott et al.^115^. Pest richness was modeled as a function of GNI, timber import volume, and forest area as below:

2 $${\log }_{10}({Richness}) \sim {\log }_{10}({GNI})+{\log }_{10}({Timber\; import})+{\log }_{10}({Forest\; area})$$

Where GNI was used as a proxy for national monitoring and reporting capacity, forest area represented host resource availability, and timber import volume represented potential introduction pressure through trade. Standardized residuals from this model were used to identify regions where observed pest richness deviates from expectations based on these drivers (Supplementary Fig. 5).

To identify potential risk zones, we overlapped the richness pattern of pests (within and outside host native ranges) and three critical forest ecosystem services, including timber-producing areas, forest diversity, and potential carbon stock, respectively. Potential risk zones were defined as grid cells where both pest richness and ecosystem service values fall within the top 50% of their respective distributions (Supplementary Fig. 6). To account for co-occurrence between the three ecosystem services, we quantified spatial overlaps among services using Venn diagram classification (Fig. 2a, b), categorizing potential risk zones into single-service zones, dual-service overlap zones, and triple-service overlap zones. We identified main exposure regions for forest pests within and outside host native ranges based on exposure areas across countries (Fig. 3c, d). We further computed the total timber area, forest diversity, and potential carbon stock within exposed zones and national potential risk zones proportions relative to total forest coverage using country boundaries from the “rnaturalearth” package in R^116^.

To explore the spatial relationship between different taxonomic forest pests and ecosystem services, we applied the global bivariate Moran’s *I* using the “spdep” package^117^ to assess the spatial association between their distributions in main exposure regions (Supplementary Table 1). The Moran’s *I* helps determine if the two variables exhibit a tendency to spatially cluster together (positive) or disperse (negative). We determined the optimal neighborhood size by iteratively constructing *k*-nearest-neighbor graphs with *k* ranging from 1 to 20 and selecting the value of *k* that maximized the global bivariate Moran’s *I* statistic. Using the optimal neighborhood *k*, we rebuilt the standardized weights matrix to ensure each grid cell’s influence sums to one and to mitigate edge effects via a zero policy. We then applied the global bivariate Moran’s *I* test with 499 random permutations to assess statistical significance by comparing the observed Moran’s *I* with a permutation-based null distribution. Finally, we generated bootstrap confidence intervals at the 95% level to quantify uncertainty. Statistical significance was assessed based on whether the 95% confidence interval excluded zero for the spatial association between pest richness and ecosystem services.

We further used the random forest (RF) machine learning algorithm using the “ranger” package^118^, along with SHAP using the “fastshap” package^119^ to quantify the relative importance of the different factors on high-risk zones. The response variable was the presence of potential risk zones identified from the spatial overlap between pest richness and ecosystem services. Predictor variables included environmental, landscape, and socioeconomic factors. Although Random Forests are inherently robust to multicollinearity, we removed several highly correlated climate predictors (*r* > 0.9) to improve computational efficiency and model interpretability. Nine variables were retained for final model construction, including mean temperature (BIO1), isothermality (BIO3), mean precipitation (BIO12), forest age, forest type, GNI, timber import volume, population density, and travel time. The random forest algorithm is an ensemble-method built-on decision tree to capture complex nonlinear interactions among predictors, and widely used to process large-scale data with highly-dimensional features. While the random forest algorithm offers the Gini importance which reflects the predictive power of each variable in the model, but it does not indicate whether that variable has a positive or negative effect on the predictions. To address this limitation, we employed a robust interpretability framework based on Shapley values from game theory. SHAP quantifies the contribution of each variable by fairly allocating the prediction impact across all possible variable combinations (Fig. 4).

For each forest ecosystem service, we adopted spatial cross-validation with three spatial fold partitions in the data to control spatial autocorrelation and avoid overoptimistic performance estimates. The block size was determined using the “blockCV” package^120^ to estimate the spatial autocorrelation range: the distance beyond which observations can be considered independent. Within each fold, we trained the RFs with 300 trees using the “ranger” package, applying adjusted weights to avoid class imbalance^118^. We then selected the hyperparameter combination that maximized mean accuracy across folds as our optimal setting, retrained the final model on the full dataset, and extracted permutation‑based variable importance scores. Given the imbalanced dataset between potential risk and non-risk cells, the majority class was downsampled within each spatial cross-validation fold to reduce potential bias during model training. The final model was retrained on the full dataset, and permutation-based variable importance scores were extracted. To evaluate model performance, we used Cohen’s Kappa statistic, which measures the agreement between predicted and observed classifications. Finally, to capture both magnitude and direction of variable effects, we computed SHAP values using 500 Monte Carlo simulations, and summarized mean absolute contributions of each variable. SHAP values were calculated for each predictor and each observation, meaning that a single predictor can have multiple SHAP values across the dataset. In the SHAP summary plots, each point represents the SHAP value for an individual observation, while the color gradient (blue to red) indicates low to high values of the corresponding predictor.

All statistical analysis was conducted in R^121^.

### Reporting summary

Further information on research design is available in the Nature Portfolio Reporting Summary linked to this article.

## Supplementary information

Supplementary Information

Reporting Summary

Peer Review file

## Data Availability

All data originally used in this study are publicly available online: global Invasive and Alien Species and their Traits database (https://doi.org/10.5281/zenodo.13138496), global map of forest loss drivers (https://data.globalforestwatch.org/documents/ tree-cover-loss-by-dominant-driver/about),global map of forest management types (https://zenodo.org/record/5879022#.ZCa9IXbMKUk), global forest diversity (https://figshare.com/articles/dataset/The_global_map_of_tree_species_richness/17232491), global potential carbon stock (https://doi.org/10.5281/zenodo.10021968.), forest cover 2000 (https://earthenginepartners.appspot.com/science-2013-global-forest/download_v1.7.html), travel time to urban center (https://figshare.com/articles/dataset/Travel_time_to_cities_and_ports_in_the_year_2015/7638134/3), forest fragmentation index (https://figshare.com/s/21dbf1f50250aeb7f5a0), forest age (https://doi.org/10.17871/ForestAgeBGI.2021), GDP, global population density (https://developers.google.com/earth-engine/datasets/catalog/CIESIN_GPWv411_GPW_Population_Density). The processed data generated in this study are publicly available in Zenodo (https://doi.org/10.5281/zenodo.21951970).

## Code availability

The code used in the present study is available at https://github.com/bobilong/forest_pests [https://doi.org/10.5281/zenodo.21951970].
