# Supplementary material for: Potential exposure risk of global timber production, carbon stocking, and biodiversity to forest pests
Source: Nat Commun. 2026 Oct 15;17:11643. doi: 10.1038/s41467-026-78728-x (PMC13759459; doi:10.1038/s41467-026-78728-x)
Supplement: Supplementary file 1 [file 41467_2026_78728_MOESM1_ESM.pdf]

Supplementary Information for:

Potential exposure risk of global timber production, carbon stocking, and biodiversity to forest pests

Yuxin Qiao[1,2*], Jianquan Liu[1*], David P. Edwards[2,3*]

[1]State Key Laboratory of Herbage Innovation and Grassland Agro-Ecosystem, College of Ecology, Lanzhou University, Lanzhou, China

[2]Department of Plant Sciences and Centre for Global Wood Security, University of Cambridge, Cambridge, UK.

[3]Conservation Research Institute, University of Cambridge, Cambridge, UK

*Correspondence: yq303@cam.ac.uk; liujq@nwipb.ac.cn; dpe29@cam.ac.uk

Supplementary Figures

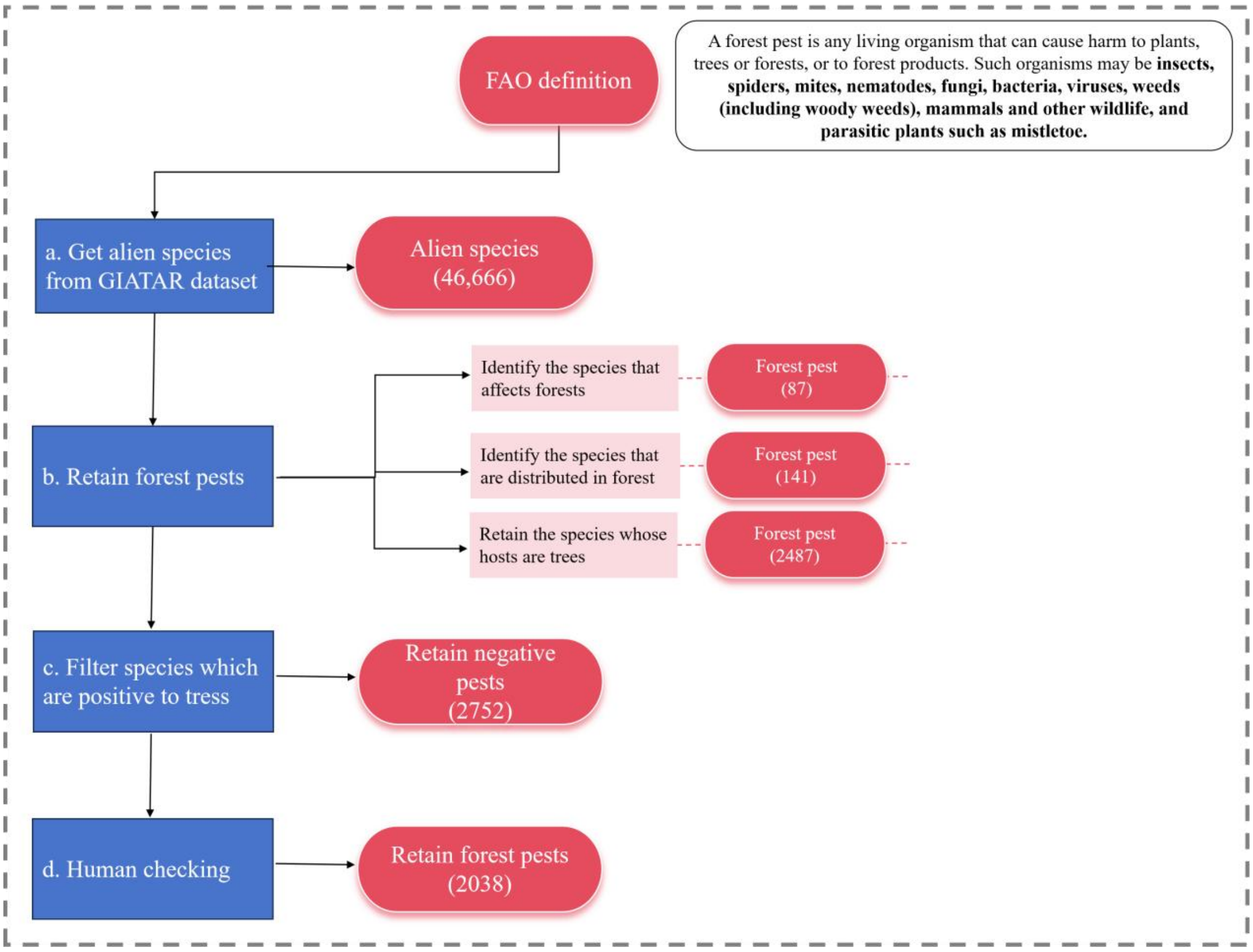

Supplementary Fig. 1 Workflow for the identification and filtering of the global forest pest. The flowchart shows the stepwise procedure used to compile the final forest pest dataset based on the FAO definition of forest pest. Forest pests were determined using three criteria including species reported to affect forests, species distributed in forest habitats, and species whose hosts are trees. Species with negative impacts on trees or forests were retained, and the resulting records were further checked manually to remove uncertain or misclassified cases, yielding a final dataset of 2,038 forest pests.

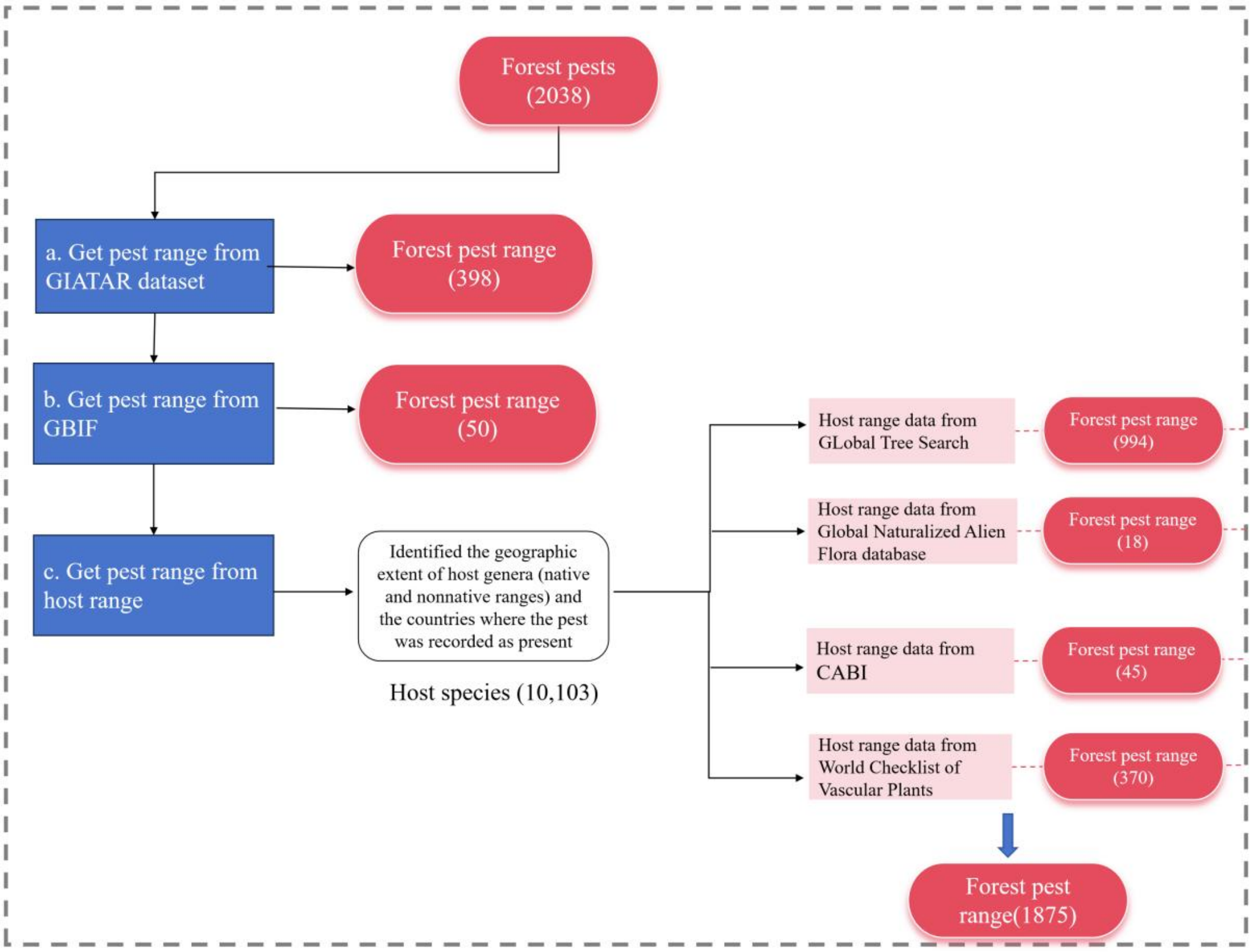

Supplementary Fig. 2 Workflow for determining geographic range of forest pests. The final analyses in the main text use 1,875 pest species within and outside host native ranges.

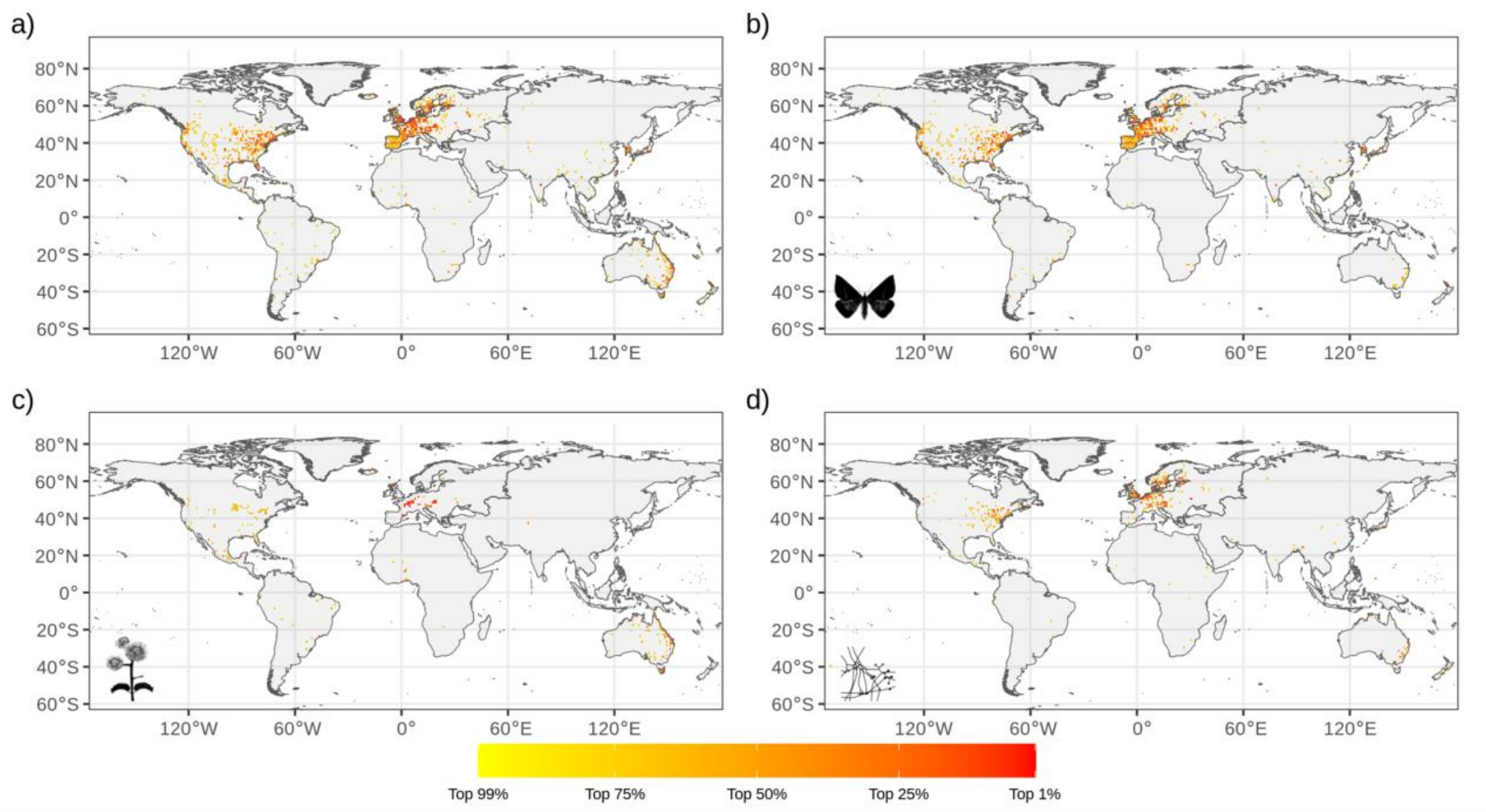

Supplementary Fig. 3 Global spatial pattern of forest pest richness. Spatial hotspots across **(a)** all, **(b)** fauna,**(c)** flora, and **(d)** pathogen forest pests. Colours represent the relative ranking of pest richness across grid cells expressed as percentiles, where higher values (top 1%) indicate greater richness and lower values (top 99%) indicate lower richness, displayed using a continuous yellow-to-red gradient. To improve display, all maps have been resampled to 1° resolution.

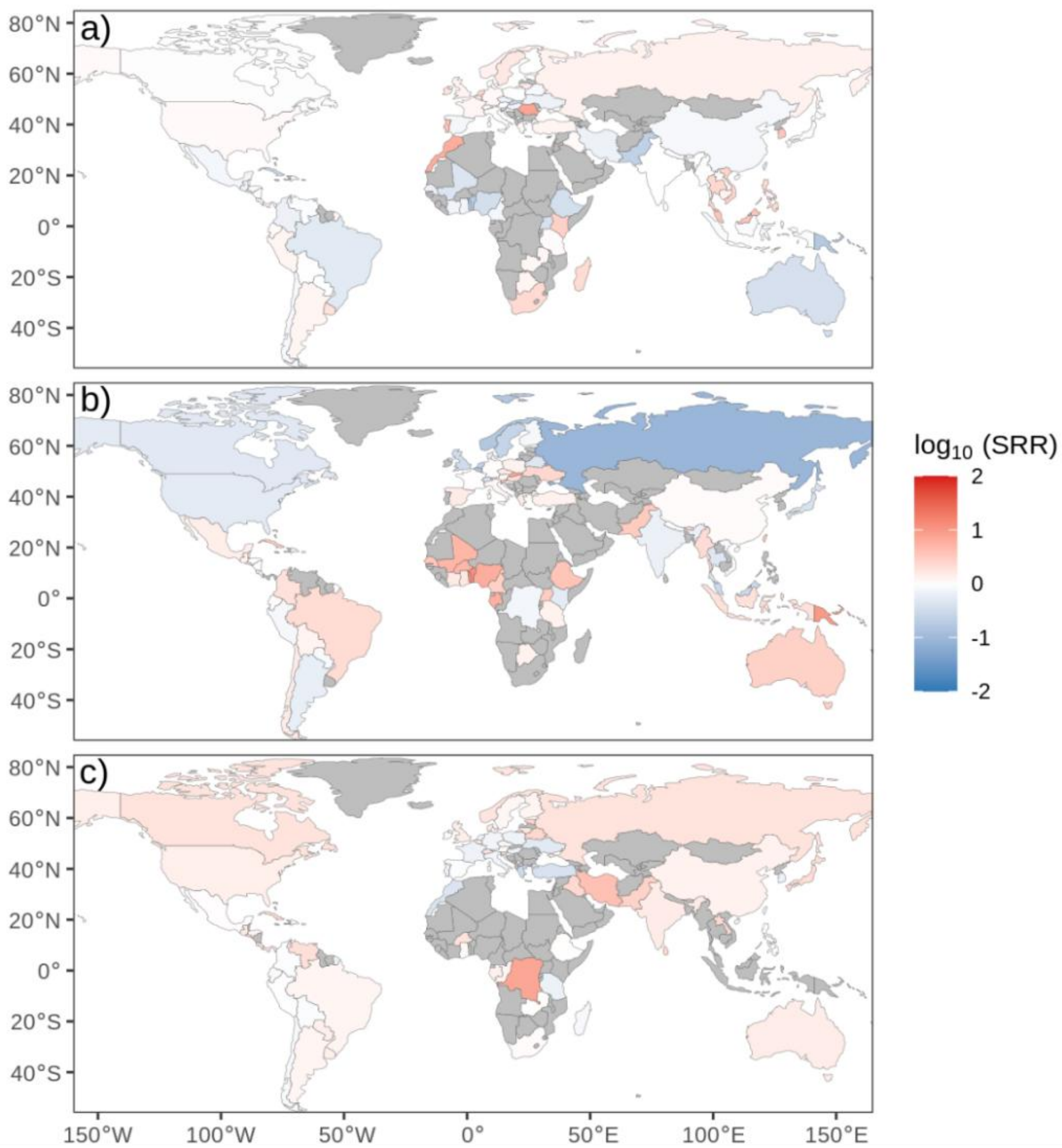

Supplementary Fig. 4 Standardized report rate (SRR) in forest pest richness.

Global distribution of SRR for (a) fauna, (b) flora, and (c) pathogen forest pest at the country level. Positive values (red) indicate that a given pest group accounts for a higher forest pests richness in a country than expected from the global average, whereas negative values (blue) indicate lower than expected. Countries with fewer than 10 records for the pest group being assessed were treated as insufficiently sampled and are shown in grey..

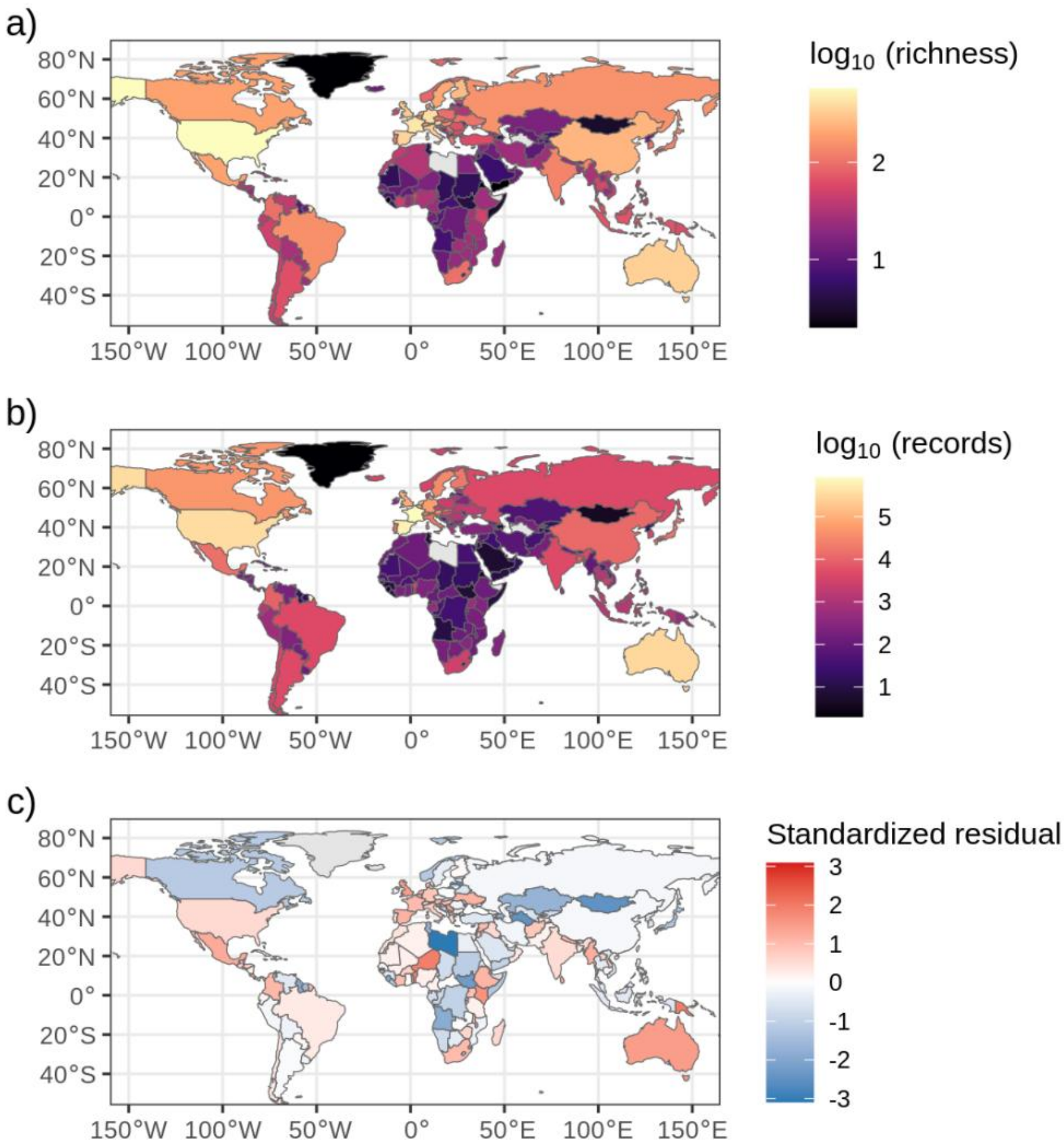

Supplementary Fig.5 Country-level forest-pest richness, observation density and residual richness patterns. (a) Total forest pests species richness per country, used as the response variable in the residual model. **(b)** Total number of forest pests occurrence records per country, used as an indicator of country-level recording intensity. In **a** and **b**, colours represent log10-transformed richness and records, respectively, with darker colours indicating lower values and lighter yellow colours indicating higher values. **(c)** Standardized residuals from the model relating country-level forest pests richness to gross national income, timber import volume and forest area. Positive (red) value indicates countries where observed richness was higher than predicted by the model, whereas negative (blue) value indicates countries where observed richness was lower than predicted. Grey indicates countries with

missing covariate data or insufficient information that were not included in the residual model.

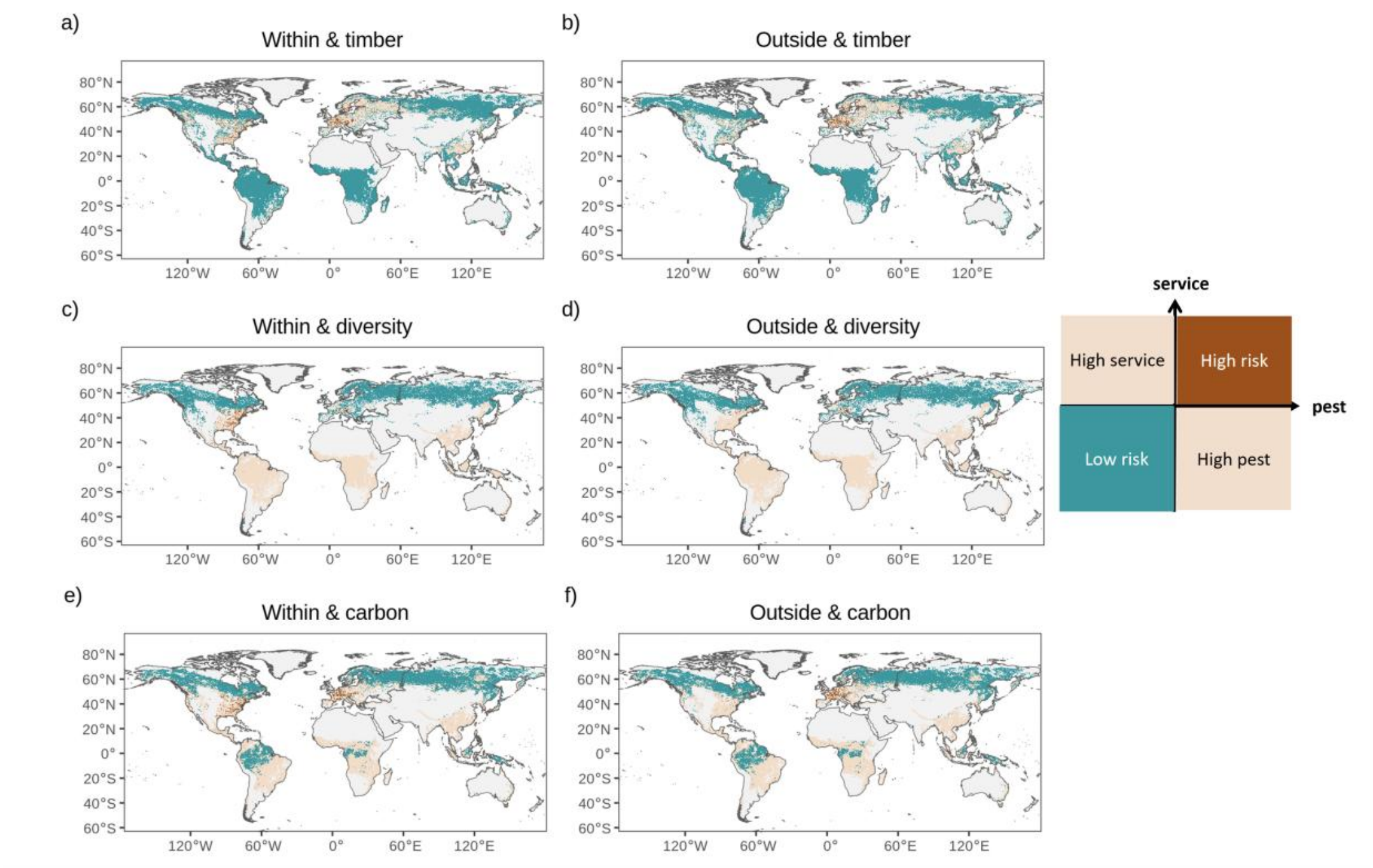

Supplementary Fig. 6 Spatial distribution overlap analysis between different geographic range of forest pests and ecosystem services. Spatial pattern of forests pests within host native ranges between **(a)** timber-producing area, **(c)** forest diversity and **(e)** potential carbon stock. Spatial pattern of forests pests outside host native ranges between **(b)** timber-producing area, **(d)** forest diversity and **(f)** potential carbon stock. The brown color indicated the grid cell where has both high forest pests and ecosystem services, whereas blue indicated the grid cell where has both low forest pests and ecosystem services.

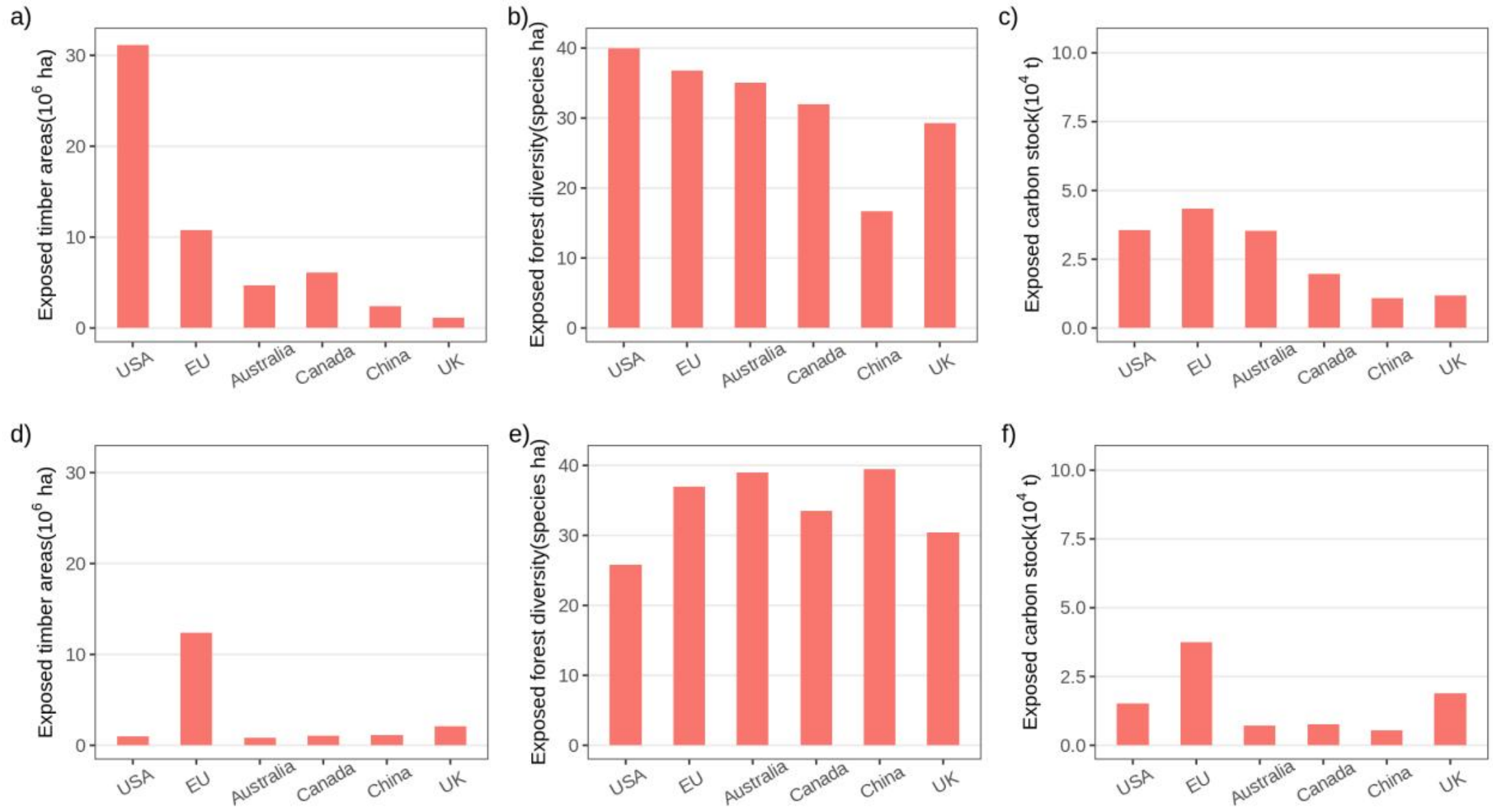

Supplementary Fig. 7 Statistics of exposed ecosystem services to forest pests in main exposure zones. Statistics of **(a, d)** timber-producing areas, **(b, e)** forest diversity, and **(c, f)** potential carbon stock exposed to pests within (**a, b, c**) and outside (**d, e, f**) host native range, respectively.

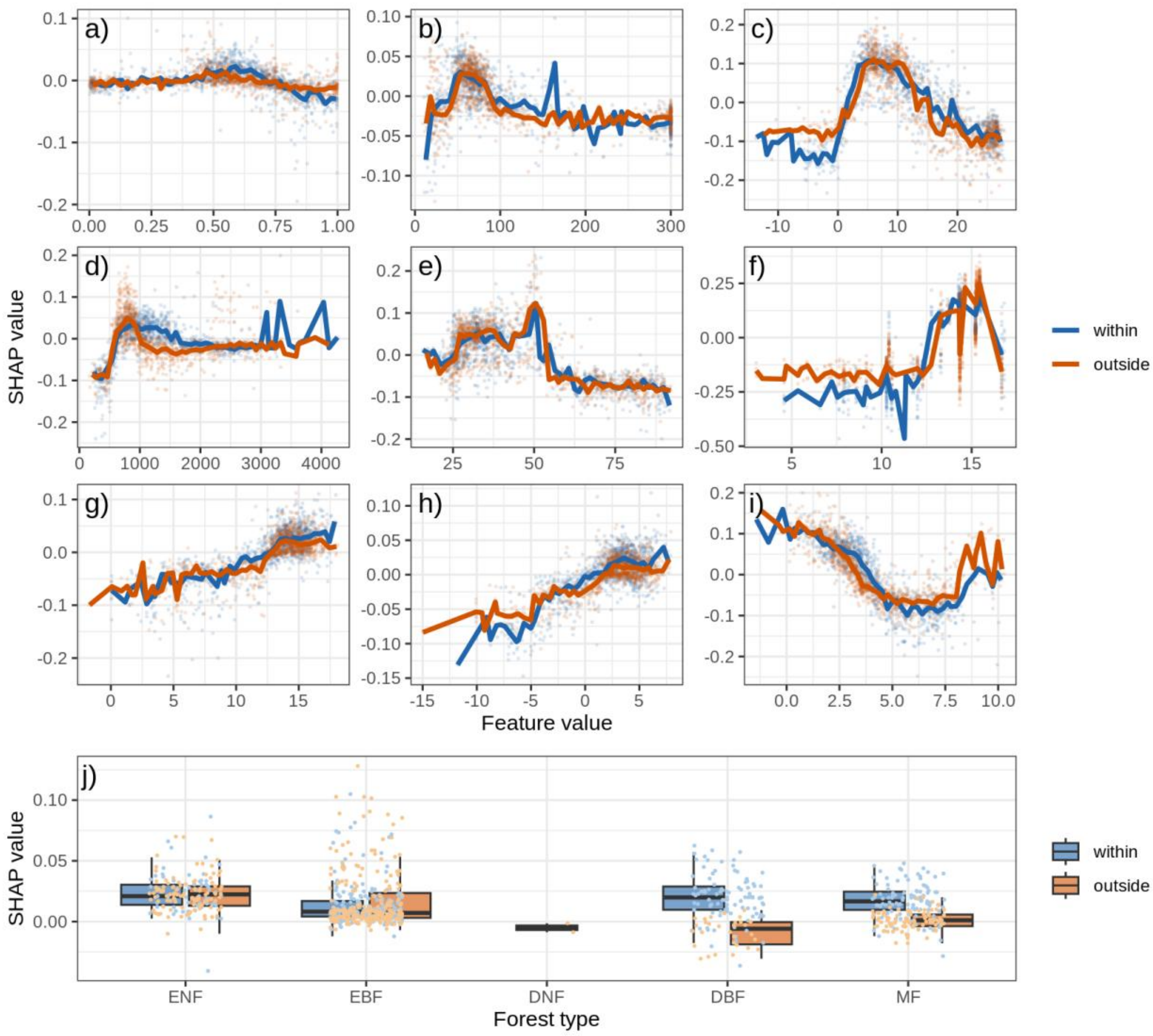

Supplementary Fig. 8 SHAP dependence plots of environmental and socioeconomic variables associated with forest pest risk in timber-producing areas. Panels show the relationships between feature values and SHAP values for **(a)** forest fragment index (FFI), **(b)** forest_age, **(c)** mean temperature, (d) mean precipitation, **(e)** isothermality, **(f)** timber import, **(g)** Gross National Income (GNI), **(h)** population density, **(i)** travel_time, and **(j)** forest type. The y-axis represents the SHAP value and the x-axis represents the corresponding feature value. Blue and orange lines indicate the median SHAP values across 50 bins of the feature values for locations within and outside host native range, respectively. Points represent individual observations. Panel **(j)** shows the distribution of SHAP values among

different forest types including ENF (evergreen needleleaf forest), EBF (evergreen broadleaf forest), DNF (deciduous needleleaf forest), DBF (deciduous broadleaf forest), and MF (mixed forest).

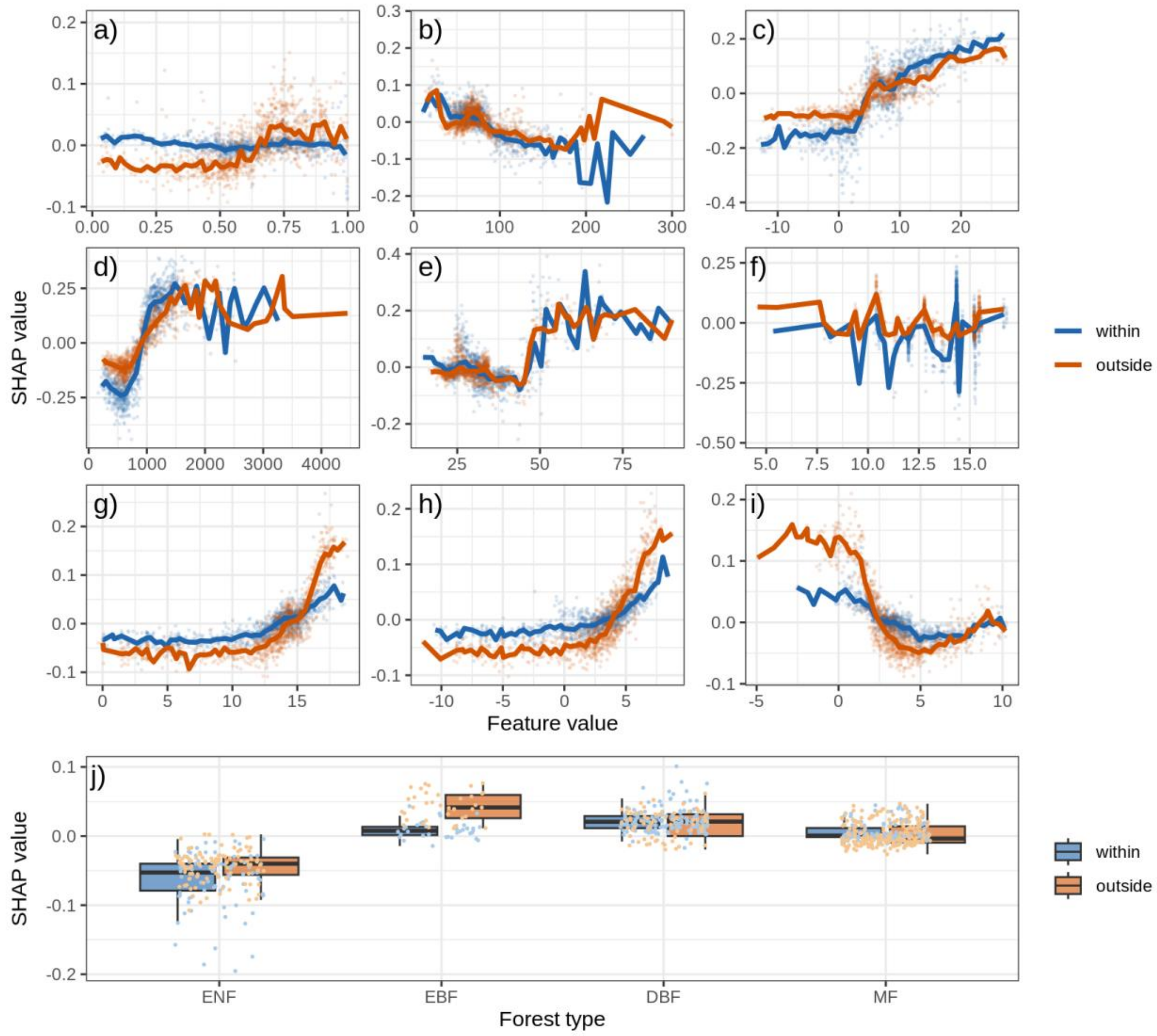

Supplementary Fig. 9 SHAP dependence plots of environmental and socioeconomic variables associated with forest pest risk in forest diversity. Panels show the relationships between feature values and SHAP values for **(a)** forest fragment index (FFI), **(b)** forest_age, **(c)** mean temperature, **(d)** mean precipitation, **(e)** isothermality, **(f)** timber import, **(g)** Gross National Income (GNI), **(h)** population density, **(i)** travel_time, and **(j)** forest type. The y-axis represents the SHAP value and the x-axis represents the corresponding feature value. Blue and orange lines indicate the median SHAP values across 50 bins of the feature values for locations within and outside host native range, respectively. Points represent individual observations. Panel **(j)** shows the distribution of SHAP values among different forest types including ENF

(evergreen needleleaf forest), EBF (evergreen broadleaf forest), DNF (deciduous needleleaf forest), DBF (deciduous broadleaf forest), and MF (mixed forest).

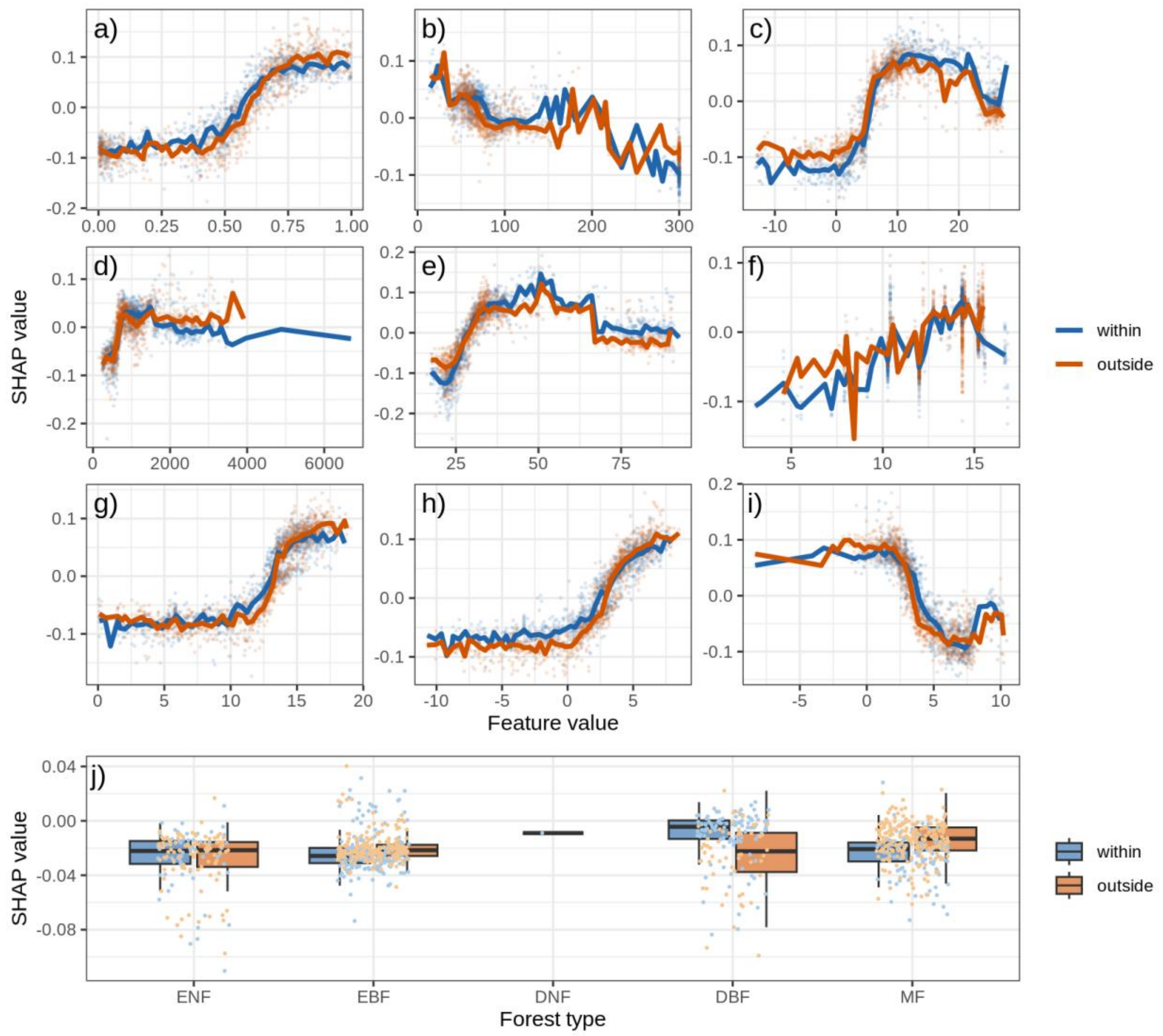

Supplementary Fig. 10 SHAP dependence plots of environmental and socioeconomic variables associated with forest pest risk in potential carbon stock. Panels show the relationships between feature values and SHAP values for **(a)** forest fragment index (FFI), **(b)** forest_age, **(c)** mean temperature, (d) mean precipitation, **(e)** isothermality, **(f)** timber import, **(g)** Gross National Income (GNI), **(h)** population density, **(i)** travel_time, and **(j)** forest type. The y-axis represents the SHAP value and the x-axis represents the corresponding feature value. Blue and orange lines indicate the median SHAP values across 50 bins of the feature values for locations within and outside host native range, respectively. Points represent individual observations. Panel **(j)** shows the distribution of SHAP values among different forest types including ENF

(evergreen needleleaf forest), EBF (evergreen broadleaf forest), DNF (deciduous needleleaf forest), DBF (deciduous broadleaf forest), and MF (mixed forest).

Supplementary Table 1 Spatial relationship between different functional groups forest pests and ecosystem services in main exposure zones.

Regions	Groups	Types	Timber areas		Forest diversity		Potential Carbon stock	
			Moran's I (95% CI)	P	Moran's I(95% CI)	P	Moran's I(95% CI)	P
USA	Fauna	Within	-0.13 (-0.15, -0.11)	*P=0.001*	0.20(0.16, 0.25)	*P=0.001*	0.04(0.02, 005)	*p=0.001*
		Outside	-0.12(-0.11, -0.13)	*p=0.001*	0.09(0.04, 0.15)	*p=0.001*	-0.14(-0.16, -0.11)	*p=0.001*
	Flora	Within	-0.08(-0.10, -0.05)	*p=0.001*	0.23(0.19, 0.26)	*p=0.001*	-0.10(-0.13, -0.07)	*p=0.001*
		Outside	-0.05(-0.08, -0.02)	*p=0.001*	0.02(0.01, 0.03)	*p=0.001*	-0.03(-0.05, -0.01)	*p=0.001*
	Pathogenic	Within	-0.11(-0.15, -0.07)	*p=0.001*	0.09(0.06, 0.13)	*p=0.001*	-0.02(-0.03, -0.01)	*p=0.001*
		Outside	-0.06(-0.10, -0.02)	*p=0.001*	0.03(0.01, 0.05)	*p=0.001*	0.03(0.02, 0.05)	*p=0.001*
Canada	Fauna	Within	-0.13(-0.14, -0.11)	*p=0.001*	0.22(0.19, 0.25)	*p=0.001*	0.17(0.11, 0.20)	*p=0.001*
		Outside	-0.15(-0.17, -0.11)	*p=0.001*	-0.01(-0.04,0.03)	*p=0.23*	0.22(0.17, 0.26)	*p=0.001*
	Flora	Within	0.00(-0.01, 0.01)	*p=0.96*	0.03(0.01, 0.05)	*p=0.43*	0.21(0.18, 0.24)	*p=0.001*
		Outside	-0.08(-0.01, 0.02)	*p=0.001*	0.00(-0.02, 0.02)	*p=0.98*	0.09(0.05, 0.13)	*p=0.001*
	Pathogenic	Within	-0.16(-0.19, -0.11)	*p=0.001*	0.04(0.01, 0.10)	*p=0.001*	0.25(0.20, 0.29)	*p=0.001*
		Outside	-0.09(-0.11, 0.01)	*p=0.001*	-0.11(-0.13,-0.07)	*p=0.001*	0.14(0.12, 0.15)	*p=0.001*
EU	Fauna	Within	-0.03(-0.08, 0.03)	*p=0.001*	0.19(0.11, 0.25)	*p=0.001*	-0.10(-0.16, -0.05)	*p=0.001*
		Outside	-0.09(-0.11, -0.15)	*p=0.001*	0.11(0.06, 0.15)	*p=0.001*	-0.10(-0.15, -0.05)	*p=0.001*
	Flora	Within	-0.14(-0.17, -0.11)	*p=0.001*	-0.12(-0.15,-0.08)	*p=0.001*	0.09(0.05, 0.13)	*p=0.001*
		Outside	-0.19(-0.21, -0.15)	*p=0.001*	0.21(0.20, 0.23)	*p=0.001*	0.06(0.02, 0.11)	*p=0.001*
	Pathogenic	Within	-0.05(-0.10, 0.03)	*p=0.001*	0.23(0.18, 0.26)	*p=0.001*	-0.01(-0.05, 0.02)	*p=0.56*
		Outside	-0.08(-0.11, -0.03)	*p=0.001*	0.09(0.03, 0.11)	*p=0.001*	-0.04(-0.09, -0.02)	*p=0.001*
UK	Fauna	Within	-0.06(-0.13, -0.04)	*p=0.001*	-0.07(-0.09,-0.04)	*p=0.001*	-0.03(-0.08, 0.01)	*p=0.001*
		Outside	-0.14(-0.19, -0.11)	*p=0.001*	-0.14(-0.19,-0.12)	*p=0.001*	0.06(0.02, 0.11)	*p=0.001*
	Flora	Within	0.04(0.01, 0.06)	*p=0.60*	0.28(0.25, 0.31)	*p=0.001*	0.03(0.01, 0.06)	*p=0.36*
		Outside	0.01(-0.09, 0.05)	*p=0.96*	0.32(0.28, 0.35)	*p=0.001*	0.02(0.01, 0.04)	*P=0.78*
	Pathogenic	Within	-0.07(-0.11, -0.03)	*p=0.001*	-0.07(-0.09,-0.04)	*p=0.001*	0.12(0.10, 0.15)	*p=0.001*
		Outside	-0.08(-0.12, -0.05)	*p=0.001*	-0.14(-0.17,-0.11)	*p=0.001*	0.08(0.06, 0.12)	*p=0.001*
China	Fauna	Within	-0.06(-0.04, -0.11)	*p=0.03*	0.23(0.21, 0.26)	*p=0.001*	0.10(0.06, 0.12)	*p=0.001*
		Outside	0.01(-0.08, 0.06)	*p=0.74*	0.25(0.20, 0.28)	*p=0.001*	0.22(0.19, 0.25)	*p=0.001*
	Flora	Within	-0.04(-0.10, -0.01)	*p=0.12*	0.12(0.11, 0.13)	*p=0.001*	0.22(0.18, 0.25)	*p=0.001*
		Outside	0.11(0.08, 0.15)	*p=0.13*	-0.03(-0.05,-0.01)	*p=0.01*	0.02(0.01, 0.05)	*p=0.51*
	Pathogenic	Within	-0.03(-0.09, -0.01)	*p=0.17*	-0.06(-0.12,-0.02)	*p=0.03*	-0.09(-0.11, -0.07)	*p=0.001*
		Outside	0.07(0.04, 0.11)	*p=0.25*	0.17(0.15, -0.20)	*p=0.02*	0.24(0.21, 0.27)	*p=0.001*
Australia	Fauna	Within	-0.08(-0.10, -0.06)	*p=0.001*	0.10(0.06, 0.12)	*p=0.04*	0.01(-0.04, 0.08)	*p=0.001*
		Outside	-0.09(-0.13, -0.02)	*p=0.001*	0.04(0.01, 0.08)	*p=0.001*	0.02(0.01, 0.05)	*p=0.28*
	Flora	Within	0.32(0.29, 0.37)	*p=0.001*	0.14(0.11, 0.16)	*p=0.001*	-0.10(-0.14, -0.07)	*p=0.001*
		Outside	0.00(-0.05, 0.05)	*p=0.93*	0.12(0.09, 0.15)	*p=0.001*	-0.01(-0.05, 0.03)	*p=0.56*
	Pathogenic	Within	0.31(0.27, 0.35)	*p=0.001*	0.14(0.11, 0.16)	*p=0.001*	0.11(0.08, 0.13)	*p=0.001*
		Outside	-0.10(-0.16, -0.05)	*p=0.001*	-0.09(-0.11,-0.07)	*p=0.01*	-0.08(-0.12, -0.05)	*p=0.001*
