# Supplementary material for: Potential exposure risk of global timber production, carbon stocking, and biodiversity to forest pests
Source: Nat Commun. 2026 Oct 15;17:11643. doi: 10.1038/s41467-026-78728-x (PMC13759459; doi:10.1038/s41467-026-78728-x)
Supplement: Supplementary file 2 [file 41467_2026_78728_MOESM2_ESM.pdf]

nature portfolio

Corresponding author(s): Yuxin Qiao; Jianquan Liu; David P. Edwards

Last updated by author(s): Jun 3, 2026

Reporting Summary

Nature Portfolio wishes to improve the reproducibility of the work that we publish. This form provides structure for consistency and transparency in reporting. For further information on Nature Portfolio policies, see our Editorial Policies and the Editorial Policy Checklist.

Statistics

For all statistical analyses, confirm that the following items are present in the figure legend, table legend, main text, or Methods section.

n/a	Confirmed	
☐	☒	The exact sample size (*n*) for each experimental group/condition, given as a discrete number and unit of measurement
☒	☐	A statement on whether measurements were taken from distinct samples or whether the same sample was measured repeatedly
☐	☒	The statistical test(s) used AND whether they are one- or two-sided<br>*Only common tests should be described solely by name; describe more complex techniques in the Methods section.*
☐	☒	A description of all covariates tested
☒	☐	A description of any assumptions or corrections, such as tests of normality and adjustment for multiple comparisons
☐	☒	A full description of the statistical parameters including central tendency (e.g. means) or other basic estimates (e.g. regression coefficient) AND variation (e.g. standard deviation) or associated estimates of uncertainty (e.g. confidence intervals)
☐	☒	For null hypothesis testing, the test statistic (e.g. *F*, *t*, *r*) with confidence intervals, effect sizes, degrees of freedom and *P* value noted<br>*Give P values as exact values whenever suitable.*
☒	☐	For Bayesian analysis, information on the choice of priors and Markov chain Monte Carlo settings
☒	☐	For hierarchical and complex designs, identification of the appropriate level for tests and full reporting of outcomes
☐	☒	Estimates of effect sizes (e.g. Cohen's *d*, Pearson's *r*), indicating how they were calculated

Our web collection on statistics for biologists contains articles on many of the points above.

Software and code

Policy information about availability of computer code

Data collection	All data were obtained from existing publicly available datasets.
Data analysis	All data processing, statistical and spatial analyses were conducted in R (version 4.3.2), using taxize (version 0.10.1), rnaturalearth (version 1.2.0), spdep (version 1.4-2), ranger (version 0.18.0), fastshap (version 0.1.1), and blockCV (version 3.2-0). Analyses included linear regression, global bivariate Moran’s I, random forest, SHAP analysis and spatial block cross-validation. The code was available on Github:https://github.com/bobilong/forest_pests.

For manuscripts utilizing custom algorithms or software that are central to the research but not yet described in published literature, software must be made available to editors and reviewers. We strongly encourage code deposition in a community repository (e.g. GitHub). See the Nature Portfolio guidelines for submitting code & software for further information.

Data

Policy information about availability of data

All manuscripts must include a data availability statement. This statement should provide the following information, where applicable:

- Accession codes, unique identifiers, or web links for publicly available datasets
- A description of any restrictions on data availability
- For clinical datasets or third party data, please ensure that the statement adheres to our policy

All data originally used in this study are publicly available online: global Invasive and Alien Species and their Traits database (https://doi.org/10.5281/zenodo.13138496), global map of forest loss drivers (https://data.globalforestwatch.org/documents/

tree-cover-loss-by-dominant-driver/about), global map of forest management types (https://zenodo.org/record/5879022#.ZCa9IXbMKUk), global forest diversity (https://figshare.com/articles/dataset/The_global_map_of_tree_species_richness/17232491), global potential carbon stock (https://doi.org/10.5281/zenodo.10021968.), forest cover 2000 (https://earthenginepartners.appspot.com/science-2013-global-forest/download_v1.7.html), travel time to urban center (https://figshare.com/articles/dataset/Travel_
time_to_cities_and_ports_in_the_year_2015/7638134/3), forest fragmentation index (https://figshare.com/s/21dbf1f50250aeb7f5a0), forest age (https://doi.org/10.17871/ForestAgeBGI.2021), GDP (https://data.worldbank.org/indicator/NY.GDP.MKTP.CD?end=2015&start=1960), global population density (https://developers.google.com/earth-engine/datasets/catalog/CIESIN_GPWv411_GPW_Population_Density) .

Research involving human participants, their data, or biological material

Policy information about studies with human participants or human data. See also policy information about sex, gender (identity/presentation), and sexual orientation and race, ethnicity and racism.

Reporting on sex and gender	*Use the terms sex (biological attribute) and gender (shaped by social and cultural circumstances) carefully in order to avoid confusing both terms. Indicate if findings apply to only one sex or gender; describe whether sex and gender were considered in study design; whether sex and/or gender was determined based on self-reporting or assigned and methods used. Provide in the source data disaggregated sex and gender data, where this information has been collected, and if consent has been obtained for sharing of individual-level data; provide overall numbers in this Reporting Summary. Please state if this information has not been collected. Report sex- and gender-based analyses where performed, justify reasons for lack of sex- and gender-based analysis.*
Reporting on race, ethnicity, or other socially relevant groupings	*Please specify the socially constructed or socially relevant categorization variable(s) used in your manuscript and explain why they were used. Please note that such variables should not be used as proxies for other socially constructed/relevant variables (for example, race or ethnicity should not be used as a proxy for socioeconomic status). Provide clear definitions of the relevant terms used, how they were provided (by the participants/respondents, the researchers, or third parties), and the method(s) used to classify people into the different categories (e.g. self-report, census or administrative data, social media data, etc.) Please provide details about how you controlled for confounding variables in your analyses.*
Population characteristics	*Describe the covariate-relevant population characteristics of the human research participants (e.g. age, genotypic information, past and current diagnosis and treatment categories). If you filled out the behavioural & social sciences study design questions and have nothing to add here, write "See above."*
Recruitment	*Describe how participants were recruited. Outline any potential self-selection bias or other biases that may be present and how these are likely to impact results.*
Ethics oversight	*Identify the organization(s) that approved the study protocol.*

Note that full information on the approval of the study protocol must also be provided in the manuscript.

Field-specific reporting

Please select the one below that is the best fit for your research. If you are not sure, read the appropriate sections before making your selection.

☐ Life sciences ☐ Behavioural & social sciences ☒ Ecological, evolutionary & environmental sciences

For a reference copy of the document with all sections, see nature.com/documents/nr-reporting-summary-flat.pdf

Ecological, evolutionary & environmental sciences study design

All studies must disclose on these points even when the disclosure is negative.

Study description	This study used a global observational spatial design based entirely on existing datasets. Forest-pest occurrence and host-range data were integrated with spatial datasets of timber production, tree diversity and potential carbon stock to quantify geographic patterns and potential exposure zones. Analyses were conducted primarily at the 10-km grid-cell scale, with some results summarized at the country level. No experimental treatments, factorial interactions, experimental units or biological replicates were involved.
Research sample	The study sample comprised 2,038 forest-pest taxa, including fauna, flora and pathogenic pests, identified from existing global databases following the FAO definition of forest pests. Of these, 1,875 taxa with sufficient occurrence and host-range information were used for the spatial analyses, supported by over 7 million GBIF occurrence records. Host and geographic-range information was obtained from GIATAR, GlobalTreeSearch, GloNAF, CABI and the World Checklist of Vascular Plants. This sample was selected to provide broad global coverage of forest pests relevant to forest ecosystem services. It represents forest-pest taxa with sufficient occurrence and host-distribution information available in existing global databases. Sex and age were not applicable because the study used species-level occurrence and distribution records rather than individual organisms, and no organisms were manipulated.
Sampling strategy	We used existing global datasets and applied predefined inclusion criteria to identify forest-pest taxa and occurrence records. Forest pests were selected following the FAO definition and filtered according to documented impacts, habitats and host associations. No a priori sample-size calculation was performed because the study did not involve experimental sampling; sample size was determined by the availability of records meeting the predefined criteria. The resulting dataset comprised 2,038 forest-pest taxa, of which 1,875 had sufficient occurrence and host-range information for spatial analyses, supported by over 7 million occurrence records, providing broad global taxonomic and geographic coverage.

Data collection	This study used existing global datasets and involved no primary field data collection by the authors. Occurrence, trait and distribution data were originally recorded by the contributing researchers and institutions of the respective databases, including GBIF, GIATAR, GlobalTreeSearch, GloNAF, CABI and the World Checklist of Vascular Plants, following their own data-collection protocols. We retrieved and harmonized these published records electronically for analysis; therefore, no field instruments were used by the authors.
Timing and spatial scale	Forest-pest occurrence records used in the spatial analyses were obtained from GBIF and spanned 1970–2022. Because these records were compiled from existing databases rather than collected under a standardized sampling programme, there was no fixed sampling frequency or predefined sampling cohort. The data had global geographic coverage, and occurrence records were standardized to 10-km grid cells for spatial analyses. Other environmental and ecosystem-service datasets were global spatial datasets obtained from the sources described in the manuscript.
Data exclusions	To ensure the accuracy of the spatial analysis, several exclusion criteria were applied to the raw dataset. We excluded records with high coordinate uncertainty (greater than 10 km) and those lacking specific geographic coordinates. Duplicate records and fossil remains were also removed. Furthermore, we excluded pest species that are primarily associated with agricultural crops rather than forest ecosystems. For the spatial risk assessment, any grid cells with less than 1% forest cover were excluded from the analysis to focus specifically on established forest biomes and reduce noise from non-forest land use.
Reproducibility	To ensure the reproducibility of this study, all analytical workflows were conducted using standardized R scripts. The primary datasets, including the cleaned pest occurrence records and spatial risk layers, are maintained in accessible formats (e.g., CSV, GeoTIFF). Detailed descriptions of the data filtering protocols, environmental variable selection, and Random Forest modeling parameters are provided in the Methods section. Furthermore, the code for data processing and spatial mapping will be made available on public repository (https://github.com/bobilong/forest_pests), and all underlying raw data sources are publicly accessible through platforms like GBIF and the CABI Compendium.
Randomization	Randomization was implemented at multiple stages of the statistical analysis to ensure model robustness. During the risk factor analysis, the dataset was randomly partitioned into a training set (80%) and a testing set (20%) for the Random Forest models. Furthermore, the Random Forest algorithm itself utilized bootstrap aggregating (bagging), where each decision tree was grown using a randomized subset of the data. To account for spatial sampling bias, we also employed a spatial thinning approach and generated pseudo-absence points by randomly sampling from areas outside the known distribution of specific pests, ensuring that the spatial models were not biased toward high-reporting regions.
Blinding	Blinding was not applicable to this study as it does not involve clinical trials or human/animal subjects that could be influenced by knowledge of the treatment. The research is a macroecological analysis based on objective observational data (pest occurrence records and satellite-derived environmental layers). Data processing and statistical modeling were conducted using standardized automated scripts to ensure that the investigator's expectations did not influence the quantitative results.

Did the study involve field work? ☐ Yes ☒ No

Reporting for specific materials, systems and methods

We require information from authors about some types of materials, experimental systems and methods used in many studies. Here, indicate whether each material, system or method listed is relevant to your study. If you are not sure if a list item applies to your research, read the appropriate section before selecting a response.

Materials & experimental systems

n/a	Involved in the study	
☒	☐	Antibodies
☒	☐	Eukaryotic cell lines
☒	☐	Palaeontology and archaeology
☐	☒	Animals and other organisms
☒	☐	Clinical data
☒	☐	Dual use research of concern
☐	☒	Plants

Methods

n/a	Involved in the study	
☒	☐	ChIP-seq
☒	☐	Flow cytometry
☒	☐	MRI-based neuroimaging

Animals and other research organisms

Policy information about studies involving animals; ARRIVE guidelines recommended for reporting animal research, and Sex and Gender in Research

Laboratory animals	*For laboratory animals, report species, strain and age OR state that the study did not involve laboratory animals.*
Wild animals	*Provide details on animals observed in or captured in the field; report species and age where possible. Describe how animals were caught and transported and what happened to captive animals after the study (if killed, explain why and describe method; if released, say where and when) OR state that the study did not involve wild animals.*
Reporting on sex	*Indicate if findings apply to only one sex; describe whether sex was considered in study design, methods used for assigning sex. Provide*

	data disaggregated for sex where this information has been collected in the source data as appropriate; provide overall numbers in this Reporting Summary. Please state if this information has not been collected. Report sex-based analyses where performed, justify reasons for lack of sex-based analysis.
Field-collected samples	*For laboratory work with field-collected samples, describe all relevant parameters such as housing, maintenance, temperature, photoperiod and end-of-experiment protocol OR state that the study did not involve samples collected from the field.*
Ethics oversight	*Identify the organization(s) that approved or provided guidance on the study protocol, OR state that no ethical approval or guidance was required and explain why not.*

Note that full information on the approval of the study protocol must also be provided in the manuscript.

Plants

Seed stocks	*Report on the source of all seed stocks or other plant material used. If applicable, state the seed stock centre and catalogue number. If plant specimens were collected from the field, describe the collection location, date and sampling procedures.*
Novel plant genotypes	*Describe the methods by which all novel plant genotypes were produced. This includes those generated by transgenic approaches, gene editing, chemical/radiation-based mutagenesis and hybridization. For transgenic lines, describe the transformation method, the number of independent lines analyzed and the generation upon which experiments were performed. For gene-edited lines, describe the editor used, the endogenous sequence targeted for editing, the targeting guide RNA sequence (if applicable) and how the editor was applied.*
Authentication	*Describe any authentication procedures for each seed stock used or novel genotype generated. Describe any experiments used to assess the effect of a mutation and, where applicable, how potential secondary effects (e.g. second site T-DNA insertions, mosiacism, off-target gene editing) were examined.*
