# Supplementary material for: Potential exposure risk of global timber production, carbon stocking, and biodiversity to forest pests
Source: Nat Commun. 2026 Oct 15;17:11643. doi: 10.1038/s41467-026-78728-x (PMC13759459; doi:10.1038/s41467-026-78728-x)
Supplement: Supplementary file 3 [file 41467_2026_78728_MOESM3_ESM.pdf]

nature portfolio

Peer Review File

Potential exposure risk of global timber production, carbon stocking, and biodiversity to forest pests

Corresponding Author: Dr Yuxin Qiao

This file contains all reviewer reports in order by version, followed by all author rebuttals in order by version.

Version 0:

Reviewer comments:

Reviewer #1

(Remarks to the Author)
The work by Qiao et al. is an original approach that attempts to map forest risk considering three major forest ecosystem services: timber production, diversity, and carbon stocking. The main hazard considered are pests, either fauna, flora or microbes. They found the main risk zones to be northwestern Europe and eastern North America. Additionally, they identify climatic factors, such as temperature and precipitation, and forest fragmentation as drivers of forest pest richness. The relationship between some services, such as diversity, and forest pest is interesting, and authors succeed to explain potential underlying causes. However, I found a few major concerns that may jeopardize the results and conclusions as stated in the paper.
First of all, the major diversity of forest pests and associated risks is detected in northwestern Europe and eastern North America. The authors attribute that to the historical plant movement and trade through past colonization processes. However, those regions are also mainly regions with a level of resources that allow remarkable forest monitoring and hence potentially higher reports of forest pests. Even though the authors try to reduce the bias due to observational pressure by retaining unique points par grid cell, that does not entirely solve the problem (lines 96-103). I wonder whether the authors could standardize the data by computing standardized report rates par forest pest type (fauna, flora, microbes) and country in the way is used for epidemiological data (e.g., Fabre et al. 2011. Can the emergence of pine Diplodia shoot blight in France be explained by changes in pathogen pressure linked to climate change? Global Change Biology). These data could be aligned with the gross national income, or import flow, par country (e.g., Scott et al 2019. Global biogeography and invasion risk of the plant pathogen genus Phytophthora. Environmental Science & Policy). This analysis could be presented along with the results to ponder out the impact of this bias. These data could even potentially be included in the random forest analyses. This bias is thus my main concern to the validity of the conclusions.
Another remark has to do with the relationship between forest pest richness and forest diversity. The authors report a positive correlation between tree and fauna pest richness. However, the study does not highlight high report rates of pests in the tropics, where the host tree diversity is high. How can the authors explain that? In addition, how can the authors reconcile this positive relationship with proved reductions of resistance to pests in diverse forest systems (Field et al. 2025. Tree diversity reduces pathogen damage in temperate forests: a systematic review and meta-analysis. Forest ecology and management; Civitello et al. 2015. Biodiversity inhibits parasites. PNAS)?
The classification of pests in native and non-native is important. Native pests cause usually chronic problems, if any, in contrast to non-native pests, that cause more rapid and dramatic impacts on forest ecosystems. The damage level is not commented on or considered clearly in the manuscript. Are there any data in this sense to be considered in the interpretation of the analyses? I am not sure the same risk level can be considered for native and non-native pests. How do authors consider this different risk level according to the associated damage?
Regarding the data used in the paper, timber producing zones are defined based on satellite imagery, but not all wood production can be easily captured by those methods. I wonder why the authors did not use forest industry data with timber volumes or/and even imports/exports data. Data availability and methodology are available and could be reproduced.
Analyses seem appropriate, except for the concerns I exposed above.
In terms of significance of the study, I find the most original contribution to be the risk map (three ecosystem services x vulnerability to forest pests; but see my concern for bias). This map could help plan for monitoring and adaptative programs. I think the results are of interest in my discipline.
The text is clear, except for a few comments that the authors can find in the text. However, I have a few suggestions to improve the clarity:
- I would suggest to address the components of risk consistently throughout the manuscript (hazard, vulnerability and

exposure; ICPP. 2020. The concept of risk in the IPCC Sixth Assessment Report: a summary of cross-Working Group discussions). It would even be advisable to declare each risk component at the beginning of the manuscript.
- The classification into native and non-native geographic ranges can be confusing, as native pests can affect native and non-native tree species, and vice versa. And sometimes the text leads to misunderstanding.
- Increase the size/font of the figures.
As a concluding remark, I think the conclusions should be modulated by the concerns I stated above, even remade after reanalyzing some parts of this work.

(Remarks on code availability)

Reviewer #2

(Remarks to the Author)
The authors present a comprehensive study on the concentration of destructive pests in forests worldwide, aggregated by forest service, and identify the areas that are the most vulnerable using statistical techniques such as Cohen's Kappa scores, global bivariate Moran's I, and a random forest with Shapley additive explanations. The authors describe the negative impact, spatial pattern, and transmission of forest pests and present some novel and existing tips for forestry surveillance, the importance of which is highlighted by the pressing issue of climate change. We recognise that a lot of time and effort went into this text and the accompanying figures. Both convey lots of complex aspects in a comprehensible way. In particular, the data wrangling process is noteworthy as many databases were scraped to achieve the granular level. Our main point of contention here is with respect to the use of pest richness rather than abundance (or a BD index that takes both into account). While it is indeed important to look at the number of pest species (since exposure to multiple taxa drives the probability of disease spreading upwards, yields a greater diversity of damage mechanisms, as well as increased challenges for simultaneous control), the principal issue is pest abundance as a more direct driver of damage risk and economic loss. We concede, however, that this study and its conclusions are important; we would think it would be even more applicable if abundance could have been considered as well. Also, the provided code does not allow for reproducibility; for instance, the lines of code accessing folder "root/autodl-tmp/" cannot be accessed unless a reader downloads the required files and place them in folders with the same name. The authors did provide a csv file, but no accompanying R script that actually uses the csv file provided.

General comments:

1. Forest uses include timber, diversity, and carbon stock, etc. but these can co-occur? Did you address such in-between cases?
2. Of the data obtained, 1,884 of the 2,038 records were used so we would state 1,884 in the Abstract instead of 2,038
3. Please justify splitting native and non-native pests in your analyses

Specific comments:

L163: "specialist pests can spread rapidly and cause large-scale mortality even when pest richness is low". So, pest richness alone is not a good enough proxy for forest mortality. Is this something that could also be focussed on?

L167: Please clarify the contrast.

L168 (and elsewhere): please report the actual p-value (either up to 2 or 3 decimals, your choice), instead of using "< 0.05", or "not significant".

L175: same Moran's I as the previous values – is this a mistake?

L188: why are there two Moran's I values? These are repeated throughout the section – mistake?

L198: rephrase as 'pest richness increases with host tree diversity only'

L212 (and elsewhere): there is no need to use predictor codes such as "bio_1". It would be best practice to use their actual names in the text and in Fig. 4 (see comment below on Fig. 4).

L243:The locations that require "high-resolution monitoring and rapid response strategies to prevent pest spillover threats" are large and hence, difficult to pinpoint exactly. However, we think this is beneficial information for forestry management regardless.

L252: How much 'more fragmented'?

L255: maybe offer advice on how to reduce fragmentation

L268: surveillance data must be "standardized" – what does this mean? And why?

L275: define author name abbreviations

L281: should J.L also appear in line 275 instead of J.Q.L?

L289: Is (a,b) all pests? Put letters before their description.

L301: Is the reddish-orange colour for diversity and carbon combined? Other descriptions do not match the colour legend

L343: The GBIF abbreviation is not defined.

L373-374: which ML models?

L384: How did you choose the four ‘Bioclim’ variables from the 19 options? What were the criteria?

L401: Could you please clarify what exactly is being used to calculate the proportion?

L410: “To focus the species occurrence, we retained only one unique observation point for each species within each 10 km grid cell”. Here, it seems that you have reduced species occurrence from the beginning, which may impact your results. Can you say that your risk zone percentages are precise and correct?

L433: k is referred to as the “neighbourhood” and the “spatial weight”. Please clarify.

L434: which statistic?

L436: why did you use 499 permutations?

L438: which confidence level of the three listed did you use to assess significance?

L441-453: what are the predictors you included in your RF model? What was the response variable?

L465: is this an imbalanced classification setting?

L488: the hyperlinked page is not the same as the text

Comments about figures:

Fig. 1: There are a few problems with this display.
- First, the current caption renders this figure completely unintelligible. It does not explain the differences between the left and right panels.
- Also, the caption states yellow and red highlights, which are not present in the figure. The resolution makes it very difficult to see the actual patterns being displayed.
- What are the circles at the bottom-left of panels c, e and g?
- The divergent colour palette chosen includes gray/white in the centre; this makes it impossible to distinguish from the actual gray/white background of the world map.
- What does the scale non-native to native, going from -1 to 1, represent? How is it calculated?
- use a different colour scale for (b), (d), (f), and (g)
- how do you interpret panels (b), (d), (f), and (g)?

Fig. 2: panels a and b could use the Venn diagram-style displays similarly to Suppl. Fig. 4. Currently it is difficult to distinguish all the colours quickly. Panels c and d should have a standardised y-axis range to allow for comparisons (both the left and right y-axis ranges), and preferably the same ordering in the x-axis as well. Moreover, in panels c and d, you are connecting the dots as if the continents/countries are a continuous variable, which is not appropriate for a categorical variable. Also, the y-axes are not comparable.

Fig. 3: A few issues here as well.
- The caption needs proofreading.
- Why present only the group with the highest spatial correlation? What if there is another group with a very similar spatial correlation, just slightly smaller numerically? That would be of interest too. It would be preferable to show all Moran indices using a heatmap, for instance.
- How do you interpret the grey wheel? What do wheel positions tell you? Some of the pest group diagrams are in a bolder font than others – is this intentional? So in the USA, native flora are clustered in carbon stock forests? What does this actually mean? Provide a sample interpretation in the figure caption

Fig. 4: please use the actual predictor names instead of “bio_1”, “bio_12” etc. Change the ‘Outside’ label to ‘Non-native’. How are there multiple SHAP values per biological variable? What do low and high feature values mean?

Suppl. Fig. 1: typo: “defination" should be “definition” and “postive" should be “positive”. Also, the caption is incomplete, please include a more descriptive caption.

Suppl. Fig. 3: why not use a gradient of colours throughout all plots, from top 1% to 99%, for instance? Currently the top 5% and 15% represent a very small portion of the maps and it is very difficult to see.

Suppl. Fig. 4: this display is worse than Suppl. Fig. 3 in terms of visualisation of the actual patterns. I would recommend perhaps decreasing the resolution? This way the squares would be slightly larger and could improve visualisation. Currently, there are 7 colours in the key, but it is very difficult to distinguish them.

Suppl. Fig. 6: the y-axis ranges should be standardises, at least across (a-d), (b-e) and (c-f).

Suppl. Figs. 8, 9 and 10: captions need English revision. It also states a red curve, but the curve in the figure is yellow, not red. Panel (f) displays forest type as a continuous predictor, but that is a categorical variable. Also, having the curves the same colour as the points makes visualisation more difficult. You could use darker shades for the curves, instead.

Suppl. Table 1: please inform the actual p-values rather than stating "< 0.05" or "not significant". The p-values stated as "< 0.01" are fine.

Typos/punctuation/recommended corrections:

Line 93: 'of records'
Line 142: 'impact of'
Line 173: change 'was' to 'had the', 'EU' to 'the EU'
Line 177: 'established' instead of 'establish'
Line 179: comma after 'where abundant'
Line 180: 'the' before 'resource'
Line 186: 'respectively' after '0.26'
Line 187: remove 'While', change 'were most strongest' to 'had the strongest correlation'
Line 191: 'a' before 'previous'
Line 205: 'control for'
Line 219: 'a' before 'non-linear effect'
Line 263: 'unmanned aerial vehicles'?
Line 276: change 'works were' to 'work was'
Line 290: 'pixel' instead of 'pixels', 'values' instead of 'value'
Line 301: 'intersection of'?
Line 303: 'the' before 'top'
Lines 308-310: change ' The warmer color represents the pattern of forest pests and ecosystem services is clustered, and colder color represents that is dispersed' to 'A warmer color represents a clustered pattern for forest pests and ecosystem services and a cooler colour represents a dispersed pattern'.
Line 310: change 'Where the value tends to 0 represents random pattern' to 'a value tending to 0 represents a random pattern'
Line 315: change ',' to 'for', 'area' to 'areas'
Line 317: change 'SHAP values distribution' to the 'distribution of SHAP values', 'area' to 'areas'
Line 327: 'and' after ','
Line 328: change 'encompassing' to 'encompasses'
Line 338: change 'first' to 'firstly'
Line 340: change 'fuzzy' to 'unclear'
Line 341: change 'second' to 'secondly'
Line 342: change 'third' to 'thirdly'
Line 344: change 'fourth' to 'fourthly'
Line 345: change 'pests' to 'pest'
Line 365: developed – used?
Line 366: change 'categorizing' to 'to categorize'
Line 378-380: put 'the' in front of each named map
Line 386: 'divided by' instead of 'divide'
Line 389: '.' instead of ','. 'as' instead of 'to'
Line 390: 'of' before 'at'
Line 391: 'pests' instead of 'pest'
Line 394: '.' instead of ','
Line 395: 'identify' instead of 'identifies'
Line 396: MODIS abbreviation not defined. 'the' before 'MODIS'
Line 397: 'following; instead of 'follows'
Line 404: 'the' before GBIF
Line 407: 'standards' instead of 'standard'
Line 408: 'at' before 'which'
Line 409: 'the' before 'occurrence', 'as' instead of 'because'
Line 411: 'Correspondingly' instead of 'Corresponding'
Line 412: 'removed' instead of 'filtered out'
Line 418: 'combine' instead of 'overlap'? 'the' before 'richness'
Line 420: 'areas' instead of 'area'
Line 424: 'ranges' instead of 'range'

Line 426: ‘to’ before ‘total’, ‘coverage’ instead of ‘cover’?
Line 432: ‘disperse’ instead of ‘dispersed’
Line 435: ‘one’ instead of ‘unity’
Line 441: ‘the’ before ‘”ranger”’
Line 442: ‘the’ before ‘”fastshap”’
Line 444: ‘an’ before ‘ensemble’
Line 446: ‘offers’ instead of ‘offer’, change ‘reflects importance of variable in predicting power within the model’ to ‘reflects the predictive power of a variable in the model’
Line 451: change ‘Random Forests’ to ‘RFs’?
Line 455: ‘with partitions the data into three spatial folds’ to ‘with three spatial fold partitions in the data’
Line 457: ‘the’ before ‘”blockCV”’, change comma to colon (:)
Line 459: change ‘the adjust’ to ‘adjusted’
Line 466: ‘simulations’ instead of ‘repeats’?

(Remarks on code availability)
see comments to author

Reviewer #3

(Remarks to the Author)
I co-reviewed this manuscript with one of the reviewers who provided the listed reports. This is part of the Nature Communications initiative to facilitate training in peer review and to provide appropriate recognition for Early Career Researchers who co-review manuscripts.

(Remarks on code availability)

Version 1:

Reviewer comments:

Reviewer #1

(Remarks to the Author)
I have read the manuscript and the modifications introduced by the authors, and I acknowledge the great effort they have made to include the suggested changes. The manuscript has remarkably improved in clarity and robustness. However, I still have some concerns in terms of the interpretation of the additional analysis regarding sampling bias.
My first concern is the way of calculating SRR values. The authors take reference values to be the total cases in GBIF, but reference values should be the raw number of records for a set of problems selected to be typical of the host population of interest (Fabre et al 2011), which is usually taken to be the raw number of records of all the problems detected in the country except for the case study (in that case either fauna or flora or pathogens). That means that the SRR for plants would take as reference cases all the other records except for plants. That leads to the interpretation that SRR > 1 (positive values in log scale) means that the richness is higher than the average, and SRR < 1 (negative values in log scale) means that the richness is lower than the average. I believe that this should not change enormously the computation of SRR made by the authors, but as they cite Fabre et al 2011, it should be either done that way, or explained why it is done otherwise.
Second, the SRR or scaled SRR -which I presume is the log-transformed SRR- is exactly the unbiased value of richness itself (of fauna, flora or pathogen forest pests) that the authors aimed at evaluating. The scaled SRR itself does not indicate that observation effort varies substantially across regions relative to background sampling effort for forest pest surveillance (cited from rebuttal letter); it is the comparison of SRR with the raw richness data that would indicate this (the dissimilarity with this data). While western Europe it is confirmed to host highly rich fauna, flora and pathogen forest pests, eastern North America is not, because the scaled SRR is too low (the pattern does not hold after correcting for the sampling bias). That means that the high raw richness values observed in eastern North America are due to a higher observation pressure. Western Europe presents both high unbiased richness (approximated through SRR) and high observation pressure. That means that the high observed richness is genuinely high, and not due to a high observation pressure. Therefore, this sentence in the rebuttal letter “United States shows scaled SRR values close to zero (fauna = -0.03, pathogen = -0.02, overall = -0.12), suggesting that pest occurrence records in eastern USA reflect background-level sampling effort rather than strong oversampling” is incorrect. The below-zero log-transformed SRR values (unbiased richness) indicate that actual richness is lower than expected, and the high raw values are explained by high observation pressure. All in all, SRR is an estimation of unbiased richness to be able to be compared to other values across the globe, and should be presented as such. Authors interpretation is then not accurate and can lead to wrong conclusions.
Finally, the Sahel areas as well as Greenland support remarkably high values of SRR (as guessed by the scaled values). Those are most likely an artefact of the SRR computation. This standardization works well when there is a minimal density of observations. There is no established threshold, but I would say under 10 observed values (as a very minimal value) it would not be correct to use it. Observations are almost absent from those areas in Supplementary Figure 3. One solution would be to group a few countries together into a bigger region for SRR calculation. Otherwise they should be excluded from the analyses, along with the explanation of very low observation density.
With regard to the second approach (GNI, imports and forest area), I see it as a complementary analysis to validate SRR data, the latter being the main output of the study. However, while residuals are informative, it would be even more informative to visualize the sampling effort of the countries itself showing all GBIF records per country (or whatever has been

used to calculate SRR values), along with those residuals. Otherwise, it is difficult to imagine whether the residuals are due to socio-economic or ecological drivers. I find the interpretation difficult to reconcile and assign to an underlying cause.
Figure 5 should state what grey color corresponds to.
Regarding the relationship between forest pest richness and forest diversity, I agree with the authors justification. However, it is not supported by the SRR data, which shows that tropical regions in America have not a high pest diversity, while a few countries in tropical Africa do. But this inconsistency can be understood by the fact that the characteristics of hyper-diverse forests may need an even higher sampling effort to detect the real pest diversity level in tropical areas. I suggest that the authors adapt their statements to the right interpretation of the SRR.
The other remarks have been carefully and correctly addressed, and I congratulate the authors for that effort.

(Remarks on code availability)

Reviewer #2

(Remarks to the Author)
We thank the authors for their careful revision of their manuscript and for ensuring their results are fully reproducible. We are satisfied with the authors' responses to our queries and their revised version of the text. We would recommend acceptance of the paper at this stage.

Minor comments:
Should line 22 of fig1.R refer to "./data" instead of "pests/forest_pests/data"?
The ReadMe file was a great addition to GitHub. However, some packages used in the scripts are missing from the installation section, e.g. data.table.

(Remarks on code availability)
We thank the authors for ensuring their work is fully reproducible.

Reviewer #3

(Remarks to the Author)
I co-reviewed this manuscript with one of the reviewers who provided the listed reports. This is part of the Nature Communications initiative to facilitate training in peer review and to provide appropriate recognition for Early Career Researchers who co-review manuscripts.

(Remarks on code availability)

Version 2:

Reviewer comments:

Reviewer #1

(Remarks to the Author)
I have carefully revised the changes introduced in the last version of the manuscript following previous comments and recommendations. I am now fully assured of the robustness of the analyses and the conclusions derived from it. I congratulate the authors for this effort and for the remarkable contribution that this paper does in terms of identification of global risk areas to forest pests. The only very minor suggestion I have to this version is that the authors change "SRR standardization" by " the standardization of forest pest records", as SRR contains already the notion of standardization and is thus a repetition.

(Remarks on code availability)

Open Access This Peer Review File is licensed under a Creative Commons Attribution 4.0 International License, which permits use, sharing, adaptation, distribution and reproduction in any medium or format, as long as you give appropriate credit to the original author(s) and the source, provide a link to the Creative Commons license, and indicate if changes were made.
In cases where reviewers are anonymous, credit should be given to 'Anonymous Referee' and the source.
The images or other third party material in this Peer Review File are included in the article's Creative Commons license, unless indicated otherwise in a credit line to the material. If material is not included in the article's Creative Commons license and your intended use is not permitted by statutory regulation or exceeds the permitted use, you will need to obtain permission directly from the copyright holder.
To view a copy of this license, visit https://creativecommons.org/licenses/by/4.0/

########################Point-by-point response letter ######################

########################Part A: Response to Comments######################

Reviewer #1 (Remarks to the Author):

The work by Qiao et al. is an original approach that attempts to map forest risk considering three major forest ecosystem services: timber production, diversity, and carbon stocking. The main hazard considered are pests, either fauna, flora or microbes. They found the main risk zones to be northwestern Europe and eastern North America. Additionally, they identify climatic factors, such as temperature and precipitation, and forest fragmentation as drivers of forest pest richness. The relationship between some services, such as diversity, and forest pest is interesting, and authors succeed to explain potential underlying causes. However, I found a few major concerns that may jeopardize the results and conclusions as stated in the paper.

Response: We thank the Reviewer for their constructive comments. In summary, we have now added standardized report rate analyses and residual models incorporating socio-economic and ecological covariates, which confirm that the main spatial patterns are broadly robust while also revealing clear regional variation in reporting effort. On this basis, we revised the Introduction to more explicitly frame our maps as a first-order assessment of potential hazard–exposure overlap, rather than a direct estimate of realized damage. We also now state more clearly that uneven surveillance capacity likely contributes to under-detection in some regions, especially in the tropics, and that this uncertainty should be considered when interpreting the global hotspots.

First of all, the major diversity of forest pests and associated risks is detected in northwestern Europe and eastern North America. The authors attribute that to the historical plant movement and trade through past colonization processes. However, those regions are also mainly regions with a level of resources that allow remarkable forest monitoring and hence potentially higher reports of forest pests. Even though the authors try to reduce the bias due to observational pressure by retaining unique points par grid cell, that does not entirely solve the problem (lines 96-103). I wonder whether

the authors could standardize the data by computing standardized report rates par forest pest type (fauna, flora, microbes) and country in the way is used for epidemiological data (e.g., Fabre et al. 2011. Can the emergence of pine Diplodia shoot blight in France be explained by changes in pathogen pressure linked to climate change? Global Change Biology). These data could be aligned with the gross national income, or import flow, par country (e.g., Scott et al 2019. Global biogeography and invasion risk of the plant pathogen genus Phytophthora. Environmental Science & Policy). This analysis could be presented along with the results to ponder out the impact of this bias. These data could even potentially be included in the random forest analyses. This bias is thus my main concern to the validity of the conclusions.

Response: We agree that spatial bias in biodiversity recording is a major challenge in global analyses. To address this and ensure the validity of our conclusions, we have performed several additional analyses following the Reviewer's suggested frameworks:

(1) We adopted a ratio-based standardization approach based on Fabre et al.[1]. In their framework, the Standardized Report Rate (SRR) is defined as the density of target disease reports divided by a reference density, which is weighted by a host-specific mean report rate to account for variations in reporting propensity. Following this approach, we defined observation reports as the number of records per pest type and country, and reference reports as the country-level total of all GBIF records. Furthermore, we applied a weighting factor based on the global expected frequency, defined as the proportion of global records for a given pest type relative to the total global GBIF records. This adjustment ensures that the reference reports reflect both the local sampling effort and global prevalence of the pest, allowing for comparisons across countries and pest types. We then calculated density of the observation and reference report using nonparametric kernel estimation method to estimate spatially smoothed reporting density followed Fabre et al.[1]. The SRR was computed as the ratio of observation density to reference density multiply global expected frequency. To better understand the observation deviation from background sampling effort, we scaled the SRR values using a base-10 logarithmic transformation (Supplementary Fig. 4). This

scaled result indicates whether the observation effort of a given pest group is disproportionately high (positive) or low (negative) relative to the background sampling effort for forest pest surveillance.

As shown in the updated Supplementary Fig. 4, the spatial patterns derived from the scaled SRR better account for observation bias, thereby unveiling the underlying pattern of species richness discussed in the main text. The scaled SRR results indicate that observation effort varies substantially across regions relative to background sampling effort for forest pest surveillance. Western European countries, particularly Spain (scaled SRR = 1.23 for fauna), France (0.59), Belgium (0.89), and the Netherlands (0.83), show consistently positive scaled SRR values, indicating high observation effort compared to the global background. This elevated surveillance intensity in Europe aligns with well-documented patterns in the literature, as European countries have established comprehensive forest health monitoring networks dating back several decades, including the International Co-operative Programme on Assessment and Monitoring of Air Pollution Effects on Forests (ICP Forests) and European & Mediterranean Plant Protection Organization (EPPO)[2,3]. The historical emphasis on forest protection in Europe, combined with dense scientific infrastructure and cross-border collaboration frameworks, has contributed to pest detection and reporting capacity.

In contrast, the United States shows scaled SRR values close to zero (fauna = -0.03, pathogen = -0.02, overall = -0.12), suggesting that pest occurrence records in eastern USA reflect background-level sampling effort rather than strong oversampling. Similarly, Mexico (fauna = -1.13, flora = -1.06, pathogen = -1.64) and southern South American countries also displayed negative scaled SRR values, indicating lower observation effort than expected. This pattern is consistent with known surveillance biases in occurrence datasets, where tropical and southern hemisphere regions are systematically under-represented compared to well-sampled temperate regions[4]. Australia shows positive scaled SRR values (fauna = 0.24, flora = 0.87, pathogen = 0.30), indicating adequate to above-average sampling effort. This supports the validity of pest occurrences reported in this region as mentioned in our original text. The

relatively strong surveillance capacity in Australia can be attributed to the country's strict biosecurity protocols and investment in early detection systems, driven by concerns about invasive species impacts on unique native ecosystems[5].

Overall, the SRR-scaled analysis confirms the pest hotspots in northwestern Europe and the eastern USA as robust patterns, while revealing that high surveillance effort in Europe partially inflates observed richness. Critically, it highlights that regions such as Africa and southern South America are likely under-sampled, underscoring the importance of accounting for observation bias when interpreting global pest distributions. This standardization approach strengthens our conclusions, while highlighting regions where future surveillance efforts should be prioritized to improve global forest pest monitoring coverage.

(2) Although SRR quantifies observation effort relative to global background sampling, we further quantified how socio-economic and ecological drivers affected observation bias following the Reviewer's suggestion (Supplementary Fig. 5). We constructed a null model including gross national income (GNI, as a proxy for detection capacity), forest area (available habitat), and timber import volume (introduction pressure) following Scott et al.[6]. We then analyzed the model residuals to distinguish between regions with likely underreporting (negative residuals, where observed richness is lower than expected) and hotspots of high risk (positive residuals, where observed richness exceeds predictions). Specifically, we modeled $\log_{10}$-transformed pest richness as a function of $\log_{10}$-transformed Gross national income, $\log_{10}$-transformed timber import volume, and $\log_{10}$-transformed forest area thus:

$$log_{10}(Richness) \sim log_{10}(GNI) + log_{10}(Timber\ import) + log_{10}(Forest\ area)$$

GNI was incorporated as a continuous indicator of national economic capacity potentially influencing forest pest detection and reporting, forest area was included to control for differences in host resource availability, and timber import was included to indicate pest introduction pressure through trade. This model explained 71.8% of the global variance in pest richness ($R^2 = 0.718$, $p<0.001$), indicating that socio-economic and ecological factors collectively account for a substantial proportion of the observed

geographical patterns.

Several countries show positive standardized residuals (Supplementary Fig. 5), indicating that observed pest richness exceeds predictions based on GNI, trade, and forest area. Australia also has positive SRR values (fauna = 0.24, flora = 0.87), suggesting adequate surveillance effort. However, its large positive residual (1.47) indicates that high pest richness cannot be explained by monitoring alone and likely reflects genuinely high pest diversity. Mexico shows negative SRR values (fauna = -1.13, pathogens = -1.64), indicating lower observation effort than the global background. However, its positive residual (1.42) suggests that pest richness still exceeds expectations based on its economic profile, pointing to potentially higher underlying diversity. Several Western European countries (e.g., Spain, France, Belgium, Netherlands) show positive SRR values, suggesting that while high GNI levels and extensive trade networks partially explain the elevated pest records in Europe, observed richness exceeds what these factors alone would predict. The convergence of high SRR values (Spain = 1.23, Belgium = 0.89, Netherlands = 0.83) and positive residuals for Europe is consistent with the interpretation that northwestern Europe represents both a pest-rich region and an area with intensive surveillance effort.

Notably, we observe both SRR and residuals of some tropic countries are negative. For example, Brazil shows negative SRR values (fauna = -1.52, flora = -1.90, pathogen = -1.68) and a negative standardized residual (-0.93), while Indonesia displays negative SRR (fauna = -0.43, flora = -1.38, pathogen = -1.71) and a negative standardized residual (-0.20). This pattern of convergent negative SRR and residuals for these tropical countries points to their low pest richness being due to monitoring deficiency. Following the Reviewer's suggestion, we have also integrated GNI and timber import volume into our Random Forest (RF) model as covariates.

Together, these two analyses reveal four distinct patterns that strengthen our interpretation: (1) regions with high SRR and positive residuals (e.g., Spain, Netherlands, UK) represent high pest richness with sufficient surveillance; (2) regions with near-zero SRR and small residuals (e.g., China) reflect reliable patterns, with no

strong evidence of oversampling; (3) regions with negative SRR but positive residuals (e.g., Mexico, Papua New Guinea, Kenya) indicate higher pest richness than expected but low surveillance effort; and (4) regions with both negative SRR and negative residuals (e.g., Brazil, Indonesia) represent both low surveillance effort and observed richness. These patterns support our conclusion that northwestern Europe and the eastern USA are forest pest hotspots, while also identifying regions where current records likely underestimate forest pest diversity. We have added these results into the main text and as Supplementary Fig. 4 and 5, with detailed discussion in the revised manuscript in Lines 103-107, 124-138, 541-560.

L103-107: "High richness of forest pests in Europe may also reflect historically developed surveillance infrastructure, as European countries have maintained comprehensive forest health monitoring networks for several decades, supported by dense scientific survey and cross-border collaboration[27,28]."

L124-138: "To account for these biases, we computed scaled standardized report rates (SRR) following Fabre et al.[32]. Our SRR-scaled analysis revealed that western European countries (Supplementary Fig. 4) show high observation effort, likely reflecting established forest health monitoring networks supported by dense scientific survey and cross-border collaboration[27,33]. In contrast, some Africa and South America countries show negative scaled SRR values, indicating under-sampling for forest pest observation (Supplementary Fig. 4). To further validate our findings against potential sampling bias, we constructed a null model incorporating gross national income (GNI), forest area, and timber import volume ($R^2 = 0.718$, $p < 0.01$). Critically, after controlling for these factors, northwestern European countries still exhibited positive residuals (Supplementary Fig. 5), indicating that observed pest richness in these regions exceeds model predictions and cannot be fully explained by economic capacity or introduction pressure alone. Conversely, our residual analysis revealed a distinct pattern in tropical regions, pointing to limited surveillance effort as a key factor contributing to low observed pest richness."

L541-560: "To account for potential observation bias in pest recording, we calculated a standardized report rate (SRR) following the framework of Fabre et al.[32]. For each pest type (fauna, flora, and pathogen) and country, observation reports were defined as the number of GBIF occurrence records. Reference reports were defined as the total number of GBIF records per country, representing background sampling effort. Spatial reporting densities for observation and reference records were estimated using a non-parametric kernel density estimation approach. The SRR was calculated as the ratio of observation density to reference density and weighted by the global expected frequency of each pest type (i.e., the proportion of global records belonging to that pest type relative to all GBIF records). To facilitate interpretation of deviations from background sampling effort, SRR values were log10-transformed (Supplementary Fig. 4).

To further assess the influence of socio-economic and ecological factors on reporting bias, we constructed a linear model relating pest richness to economic capacity, trade pressure, and host resource availability following the approach of Scott et al.[114]. Pest richness was modeled as a function of GNI, timber import volume, and forest area as below:

$$log_{10}(Richness) \sim log_{10}(GNI)+log_{10}(Timber\ import)+log_{10}(Forest\ area)$$

Where GNI was used as a proxy for national monitoring and reporting capacity, forest area represented host resource availability, and timber import volume represented potential introduction pressure through trade. Standardized residuals from this model were used to identify regions where observed pest richness deviates from expectations based on these drivers (Supplementary Fig. 5)."

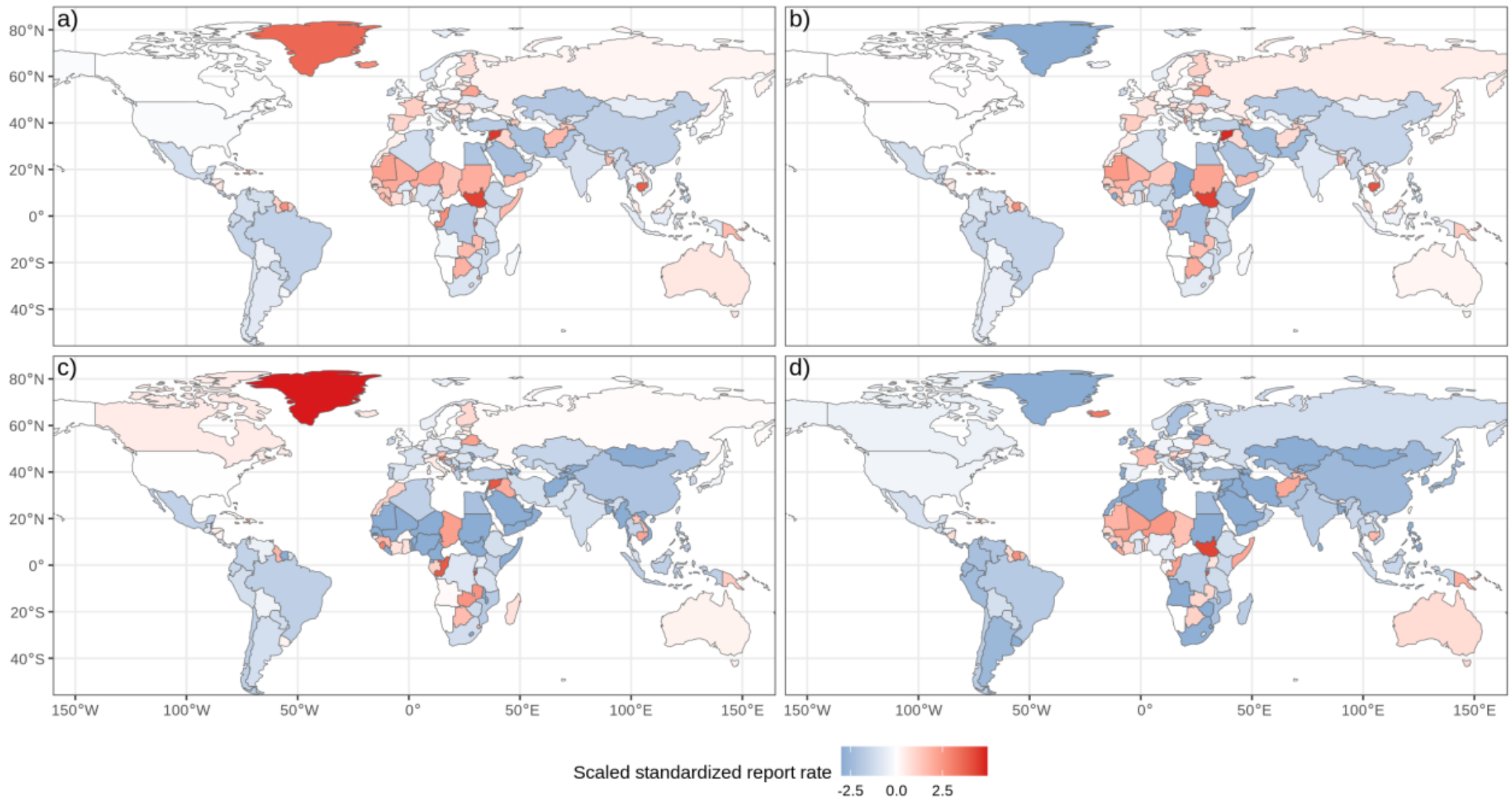

Supplementary Fig. 4 Global assessment of sampling bias in forest pest richness. Global distribution of standardized report rate (SRR) for **(a)** total , **(b)** fauna, **(c)** flora, and **(d)** Pathogen forest pest at the country level. Positive values (red) indicate regions where pest reports are disproportionately high relative to general sampling intensity, whereas negative values (blue) indicate lower than expected reporting relative to background sampling effort.

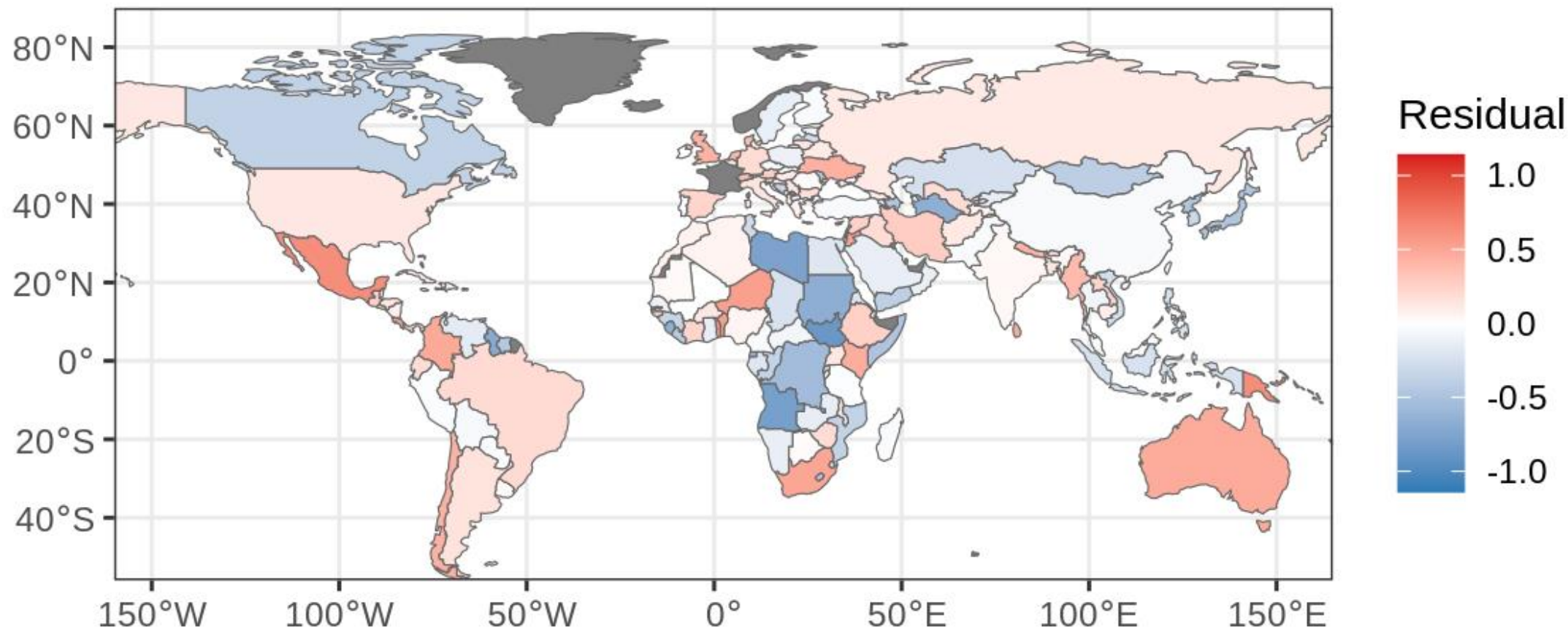

Supplementary Fig.5 Residual analysis of forest pest richness after accounting for sampling effort and socio-economic drivers. Residuals from a generalized linear

model where observed richness was regressed against Gross National Income, forest area, and timber import volume. Positive residuals (in red) highlight regions where pest richness is higher than what would be expected based solely on economic development and monitoring effort. Negative residuals (in blue) indicate regions with lower richness than predicted.

Another remark has to do with the relationship between forest pest richness and forest diversity. The authors report a positive correlation between tree and fauna pest richness. However, the study does not highlight high report rates of pests in the tropics, where the host tree diversity is high. How can the authors explain that? In addition, how can the authors reconcile this positive relationship with proved reductions of resistance to pests in diverse forest systems (Field et al. 2025. Tree diversity reduces pathogen damage in temperate forests: a systematic review and meta-analysis. Forest ecology and management; Civitello et al. 2015. Biodiversity inhibits parasites. PNAS)?

Response: We agree that the relationship between host diversity and pest dynamics is complex and strongly scale-dependent. In our analysis, we detected a positive spatial association between tree diversity and fauna pest richness. This pattern is consistent with previous studies showing that higher plant diversity can support a greater number of specialized herbivores and pathogens because a wider range of host resources and ecological niches becomes available[7]. In addition, insects in tropical regions often exhibit higher host specificity than those in temperate forests[8], a pattern that may promote diversification and contribute to the high insect diversity observed in tropical regions.

Despite the high tree diversity in tropical regions, our primary maps show lower reported richness of pests. However, our additional analyses based on the standardized report rate (SRR) and residuals (see above response, Supplementary Fig.4 and 5) suggest that this pattern is largely attributable to uneven monitoring capacity and reporting effort across regions. Specifically, many tropical countries show negative

residuals and low SRR values, indicating that their recorded pest richness falls below the levels expected. This pattern suggests that pest diversity in tropical regions is likely underestimated due to limited surveillance capacity, uneven research investment, and weaker international reporting networks[9].

Biological characteristics of tropical systems may further exemplify the effects of this monitoring deficiency. Many tropical herbivores are host-specific[8], and their host trees are often spatially dispersed within hyper-diverse forests. Consequently, local population densities of host-associated insects tend to be low. In addition, strong regulation by natural enemies can further suppress population sizes. As noted in previous studies[8], tropical pests are harder to detect because they are specialized on host species and often occur at low local densities, posing substantial challenges for systematic inventory and monitoring.

We would like to clarify our finding of a positive correlation between forest diversity and pest richness does not contradict the "dilution effect" reported by studies such as Civitello et al.[10] and Field et al.[11]. Rather, these patterns address different ecological responses operating at different levels of organization. These studies primarily focus on ecological responses such as infection prevalence, pathogen damage, or outbreak intensity. They demonstrate that increasing host diversity can reduce transmission efficiency or the severity of outbreaks for individual pest or pathogen species, often because higher diversity reduces the density of susceptible hosts within a community.

In contrast, our study quantifies pest species richness, that is, the total number of recorded pest species within a region. At macroecological scales, more diverse tree communities provide a wider range of host resources and ecological niches, which can support a larger overall pool of pest species. Therefore, a diverse forest can simultaneously host a larger number of pest species (high richness) while limiting the population growth or outbreak intensity of single species (low damage severity).

Furthermore, the role of diversity is scale-dependent. The dilution effect is commonly observed in plot-level experiments or stand-level comparisons between mixed forests and monocultures, where host density and transmission dynamics

dominate ecological process. In contrast, our analysis operates at a macroecological scale (0.1° grid cells), where spatial turnover and landscape-level host heterogeneity become increasingly important drivers of species richness patterns. Our dataset is also based on species occurrence records rather than outbreak reports or economic loss data. In this context, the classification of a species as a "pest" reflects its ecological presence in a region rather than indicating that it consistently causes damaging outbreaks across all locations.

In summary, high tree diversity, particularly in tropical regions, may support a large assemblage of pest species, while low detection probability and uneven monitoring effort can lead to underestimation of their recorded richness. These processes help explain both the positive diversity-richness relationship observed in our analysis and the apparently lower reported pest richness in parts of the tropics. We have revised the Main (Lines 103-107, Lines 138-143, Lines 245-260) to clarify these scale-dependent relationships and the context of pest detectability in macroecological analyses.

L103-107: "High richness of forest pests in Europe may also reflect historically developed surveillance infrastructure, as European countries have maintained comprehensive forest health monitoring networks for several decades, supported by dense scientific survey and cross-border collaboration[27,28]."

L138-143: "Although we focused on pest occurrence and retained unique points for each grid cell to reduce uneven observation effect[34], biological characteristics of tropical systems may still limit detectability of pest. Many tropical herbivores exhibit relatively high host specificity compared with temperate species[35], and in hyper-diverse forests where conspecific trees are spatially dispersed, host-associated insects occur at lower local densities, making detection through conventional surveys more challenging[36]."

L245-260: "While our macroecological analysis reveals a positive spatial association between tree diversity and pest species richness, this pattern supports the well-

documented dilution effect, where increased host diversity reduces the transmission efficiency or outbreak intensity of specific pests[59,60]. The dilution effect is typically observed at local scales (e.g., plot-level experiments), where high tree diversity lowers the density of susceptible hosts, thereby inhibiting the outbreak intensity of individual pest species[60]. In contrast, our study focuses on regional species richness across broad spatial extents. At this macro scale, higher tree diversity provides a wider variety of niches and host resources, facilitating the accumulation of a larger regional pool of host-specific herbivores and pathogens[61]. Consequently, a diverse forest can harbor a high richness of pest species (many specialists at low density) while simultaneously exhibiting low damage severity due to the dilution effect. Furthermore, the observed pest richness in hyper-diverse tropical regions may be underestimated due to monitoring limitations and biological characteristics[35], with many tropical herbivores exhibiting host specificity and low local abundance[35]. Such biological traits, combined with uneven surveillance efforts in tropical countries, make forest pests harder to detect, potentially generating observation bias[36]."

The classification of pests in native and non-native is important. Native pests cause usually chronic problems, if any, in contrast to non-native pests, that cause more rapid and dramatic impacts on forest ecosystems. The damage level is not commented on or considered clearly in the manuscript. Are there any data in this sense to be considered in the interpretation of the analyses? I am not sure the same risk level can be considered for native and non-native pests. How do authors consider this different risk level according to the associated damage?

Response: We agree that different pest groups can cause different levels of ecological or economic damage, and that the magnitude of damage is an important component when interpreting forest pest risks. In this study, we used the IPCC risk framework and explicitly distinguish among the components of hazard, exposure, and vulnerability following Reviewer's suggestion. Pest species richness represents the hazard component, indicating the potential presence and diversity of pest agents. The spatial

distributions of timber production, carbon storage, and tree biodiversity represent the exposure component, reflecting the presence of valuable forest assets that may be affected. Our analysis therefore focuses on identifying regions where high pest hazard overlaps with high exposure of ecosystem services.

The Reviewer notes that the realized level of damage caused by pests can vary substantially. While this is true, damage severity is primarily related to vulnerability and impact, which depend on multiple factors including host susceptibility, forest management practices, climatic suitability, and pest population dynamics. At present, globally consistent datasets quantifying pest damage severity (e.g., tree mortality rates, economic losses, or carbon reductions) are highly incomplete and unevenly reported across regions and pest taxa. As a result, incorporating a standardized damage-severity component into a global analysis remains challenging.

Following the Reviewer's earlier suggestion regarding terminology, we also revised the manuscript to distinguish between pests occurring within the native ranges of their host trees ("within host range pests") and occurring outside host native ranges ("outside host range pests") in Lines 468-470. These categories capture differences in pest-host associations and potential invasion dynamics. Nevertheless, the magnitude of damage caused by individual species within either category can vary widely, and our current hazard-based approach does not assume equivalent impact levels among pest species. To clarify this scope, we have revised the manuscript (Lines 72-79) as follows:

L72-79: "To provide a structured framework for this assessment, we adopt the IPCC Sixth Assessment Report (AR6) risk framework, which defines risk as hazard, exposure, and vulnerability[20]. Here, we conceptualize pest richness as the hazard component (i.e., the potential of pests to cause harm) and the spatial distributions of timber production, carbon storage, and tree biodiversity as the exposure component (i.e., the presence of valuable forest assets that may be affected). Accordingly, our analysis identifies potential risk zones (hazard-exposure overlap zones), characterized by the co-occurrence of high pest richness and three critical forest ecosystem services."

Regarding the data used in the paper, timber producing zones are defined based on satellite imagery, but not all wood production can be easily captured by those methods. I wonder why the authors did not use forest industry data with timber volumes or/and even imports/exports data. Data availability and methodology are available and could be reproduced. Analyses seem appropriate, except for the concerns I exposed above.

Response: We agree that forest industry statistics and trade data (e.g., timber production volumes) provide valuable information about forestry activity. However, these datasets are typically reported as aggregated statistics at national or regional administrative levels and therefore do not provide spatially explicit information on where timber harvesting occurs within countries.

Because our exposure analysis requires spatially explicit overlays between pest distributions and timber-producing areas at the grid level (0.1° resolution), national production statistics cannot reliably identify the precise locations of logging activities. For this reason, we used the remote-sensing-based dataset from Curtis et al.[12], which provides a globally consistent classification of forest disturbance drivers derived from satellite imagery. In our analysis, grid cells classified as forestry-related disturbances were used as a spatial proxy for areas with active timber harvesting, enabling spatially explicit global mapping of timber production zones.

Following the Reviewer's suggestion, we have also incorporated timber import data into our Random Forest driver analysis (Fig. 4) to evaluate the role of international wood demand in shaping global pest distribution patterns. Although timber import data cannot be spatially allocated to specific grid cells, they provide an important socioeconomic indicator of forestry activity and global trade pressure. Including this variable allowed us to examine whether international timber trade contributes to the large-scale drivers of pest occurrence. The results indicate that timber import contributes to the major explanatory power of potential risk zones of timber-producing areas.

We have revised the manuscript (Lines 479-488) thus:

"To better capture spatial precise distribution of timber production, we employed the data from Curtis et al.[96], which provides a global analysis of forest loss drivers from

2001 to 2015 using satellite imagery and machine learning classification at 10 km resolution. Using 4,985 high-resolution training samples from Google Earth imagery, the dataset used a decision-tree model categorize forest disturbances into five primary drivers, such as commodity-driven deforestation, shifting agriculture, forestry, wildfires, and urbanization. We only included grid cells associated with forestry activities as areas of timber production. To further capture the influence of wood production trade on forest pest exposure, we include timber import volume data which obtained from the Food and Agriculture Organization (FAO) FAOSTAT database[97] in driver analysis."

In terms of significance of the study, I find the most original contribution to be the risk map (three ecosystem services x vulnerability to forest pests; but see my concern for bias). This map could help plan for monitoring and adaptative programs. I think the results are of interest in my discipline.

Response: We agree that integrating timber production, carbon storage, and tree biodiversity into a spatially explicit risk map provides a more comprehensive basis for forest protection. As noted, these maps are intended as a practical tool for monitoring and adaptive management. By identifying areas where high ecosystem service value overlaps with elevated pest richness, they can help prioritize surveillance in key hotspots and guide targeted interventions in particularly vulnerable forests. Regarding the concern about potential bias, we have addressed this through additional standardization and residual analyses (see response above; now included in the Supplementary Fig. 4 and 5). The main spatial patterns remain robust after accounting for socioeconomic and sampling heterogeneity. We hope that these high-resolution risk maps will serve as a useful reference for conservation planning under accelerating biological invasions.

The text is clear, except for a few comments that the authors can find in the text. However, I have a few suggestions to improve the clarity:

- I would suggest to address the components of risk consistently throughout the manuscript (hazard, vulnerability and exposure; ICPP. 2020. The concept of risk in the

IPCC Sixth Assessment Report: a summary of cross-Working Group discussions). It would even be advisable to declare each risk component at the beginning of the manuscript.

Response: We agree that adopting a consistent framework for risk components improves conceptual clarity. Following the IPCC Sixth Assessment Report[13], we have revised the manuscript to explicitly define the three components of risk—hazard, exposure, and vulnerability—in the Introduction (Lines 65–72). In our study, pest species richness represents the hazard component, reflecting the potential presence and diversity of pest that may cause harm to forest ecosystems. The spatial distributions of timber production, carbon storage, and tree biodiversity represent the exposure component, indicating the presence of valuable forest assets that could be affected by pest pressures.

Although the IPCC risk framework includes hazard, exposure, and vulnerability, our analysis focuses specifically on the spatial overlap between hazard and exposure. Globally consistent indicators of forest vulnerability to pests (e.g., host susceptibility, forest management practices, or local ecological resilience) remain limited and are highly context dependent. Therefore, our study aims to identify hotspots of hazard-exposure overlap as a first-order risk screening approach, which highlights regions where forest ecosystem services may be particularly exposed to pest pressure.

- The classification into native and non-native geographic ranges can be confusing, as native pests can affect native and non-native tree species, and vice versa. And sometimes the text leads to misunderstanding.

Response: We agree that the original terminology could lead to misunderstanding, as a pest's biogeographic origin (native and non-native) does not directly indicate whether it occurs within or outside the native range of its host trees. To address this, we have revised our terminology and classification following the framework of Gougherty & Davies[14] (Lines 470-479). We now classify pest occurrences according to their relationship with the native ranges of their host trees. Specifically, we distinguish between pests occurring within the native ranges of their host trees ("within host native

range") and pests occurring outside the native ranges of their host trees ("outside host native range"). This classification focuses on pest-host associations and the occurrence of novel interactions resulting from host introductions, which better reflects the ecological processes relevant to forest pest dynamics. Interactions occurring outside host native ranges may represent novel pest-host encounters that can have distinct ecological consequences and management implications[15].

We have updated terminology throughout the manuscript to consistently use "pests within host native ranges" and "pests outside host native ranges".

- Increase the size/font of the figures.

Response: We have increased the font size and overall figure dimensions to improve readability in all main and supplementary figures. The revised figures now use larger axis labels, legends, and panel annotations to ensure clarity both in print and digital formats.

As a concluding remark, I think the conclusions should be modulated by the concerns I stated above, even remade after reanalyzing some parts of this work.

Response: Following the Reviewer's concerns raised above, we have conducted additional analyses and clarifications to address potential sources of bias and improve the robustness of our results. In particular, we incorporated additional tests to evaluate the influence of reporting bias and variable selection, and clarified the modelling workflow and interpretation of the Random Forest–SHAP framework. These revisions ensure that the conclusions are aligned with the updated analyses and provide a more balanced interpretation of the results.

Reviewer #2 (Remarks to the Author):

The authors present a comprehensive study on the concentration of destructive pests in forests worldwide, aggregated by forest service, and identify the areas that are the most vulnerable using statistical techniques such as Cohen's Kappa scores, global bivariate

Moran's I, and a random forest with Shapley additive explanations. The authors describe the negative impact, spatial pattern, and transmission of forest pests and present some novel and existing tips for forestry surveillance, the importance of which is highlighted by the pressing issue of climate change. We recognise that a lot of time and effort went into this text and the accompanying figures. Both convey lots of complex aspects in a comprehensible way. In particular, the data wrangling process is noteworthy as many databases were scraped to achieve the granular level.

Response: We sincerely thank the Reviewers for their thorough and highly encouraging evaluation of our manuscript. We greatly appreciate the kind acknowledgement of the time and effort invested in the text, and especially the data wrangling process.

Our main point of contention here is with respect to the use of pest richness rather than abundance (or a BD index that takes both into account). While it is indeed important to look at the number of pest species (since exposure to multiple taxa drives the probability of disease spreading upwards, yields a greater diversity of damage mechanisms, as well as increased challenges for simultaneous control), the principal issue is pest abundance as a more direct driver of damage risk and economic loss. We concede, however, that this study and its conclusions are important; we would think it would be even more applicable if abundance could have been considered as well.

Response: We agree that pest abundance is a more direct driver of realized damage and economic loss than species richness alone. Within the risk framework adopted in our study, following the approach of the IPCC Sixth Assessment Report[13], pest richness represents the hazard component, reflecting the diversity of potential pest that may affect forest ecosystems. In contrast, pest abundance or outbreak intensity would more directly capture the magnitude of realized impacts, which relates to the intensity of the hazard and to the vulnerability of affected forests.

Our use of pest richness rather than abundance was primarily driven by data availability at the global scale. To our knowledge, no globally consistent, high-resolution dataset currently exists that quantifies pest abundance, population density, or outbreak severity for thousands of forest pest species across multiple continents. In

contrast, occurrence-based data allow robust estimation of pest richness and have been widely used in macroecological studies to characterize spatial patterns of pest pressure and invasion risk.

Within the hazard-exposure framework used in this study, we therefore quantify hazard using pest richness and identify regions where this hazard overlaps with exposure, represented by high levels of timber production, carbon storage, or tree biodiversity. This approach enables a global first-order screening of areas where forests providing important ecosystem services may be exposed to multiple pest taxa. While richness does not directly measure damage severity, higher pest richness increases the likelihood that at least one species reaches outbreak levels, expands the diversity of potential damage mechanisms, and increases the complexity of pest management.

To address the Reviewers' concerns explicitly, we have added a statement in Lines 338–345 explicitly noting this limitation as follows:

L338-345: "Our analysis quantifies pest richness as a measure of hazard, rather than pest abundance or outbreak intensity. While abundance and outbreak severity more directly determine realized damage, globally consistent data on these metrics remain unavailable across taxa and regions[80]. Richness-based patterns therefore provide a practical first-order indicator for prioritizing surveillance at broad spatial scales. In this context, areas with higher pest richness may require greater monitoring attention because they contain a larger pool of taxa from which outbreaks may emerge, particularly where ecological regulation is weakened by host introduction, fragmentation, or climatic stress."

Also, the provided code does not allow for reproducibility; for instance, the lines of code accessing folder "root/autodl-tmp/" cannot be accessed unless a reader downloads the required files and place them in folders with the same name. The authors did provide a csv file, but no accompanying R script that actually uses the csv file provided.

Response: We apologise that the provided code was not fully reproducible and for the lack of a complete R script. We have now thoroughly revised the code to ensure full

transparency and reproducibility. Specifically, we have provided a clear README file, explaining the required folder structure and how to run the scripts step by step. We now include a fully documented R script, performs all analyses, and generates the main figures and tables. The complete code, along with full data and a detailed README, is now available at https://github.com/bobilong/forest_pests (the same link as in the manuscript). We thank the Reviewer again for holding us to a high standard of reproducibility, and we believe the revised code now meets that standard.

General comments:

1. Forest uses include timber, diversity, and carbon stock, etc. but these can co-occur? Did you address such in-between cases?

Response: Indeed, different forest ecosystem services-timber production, tree diversity, and carbon stock—can co-occur spatially, and we explicitly accounted for such cases in our analysis. Following the hazard-exposure framework of risk assessment[13], we treat pest richness as the hazard, while the three ecosystem services represent distinct dimensions of exposure. Potential risk zones are therefore defined as locations where high pest richness coincides with high exposure for a given service.

Importantly, these exposure dimensions are not mutually exclusive and can overlap within the same geographic area. To capture such situations, we quantified the spatial intersections among potential risk zones associated with each service (e.g., timber only, timber-carbon, timber-diversity, or all three simultaneously). The relative proportions of these co-occurrence categories are presented in Fig. 2a and b, which explicitly illustrate how multiple ecosystem services overlap within the same high-risk regions. To further clarify this point, we have added a brief explanation in the Methods section (Lines 566-571) as follows:

L566–571: “To account for co-occurrence between three ecosystem services, we quantified spatial overlaps among services using Venn diagram classification (Fig. 2a and b), categorizing potential risk zones into single-service zones, dual-service overlap zones, and triple-service overlap zones. We identified main exposure regions for forest

pests within and outside host native ranges based on exposure areas across countries (Fig.3c, d). ”

2. Of the data obtained, 1,884 of the 2,038 records were used so we would state 1,884 in the Abstract instead of 2,038

Response: Initially we collected occurrence records for 2,038 forest pest taxa. However, as noted by another Reviewers, the binary classification of pests into “native” and “non-native” ranges can be conceptually problematic and potentially misleading. To address this, we have thoroughly revised our workflow for assigning geographic range status. Specifically, we now classify each occurrence record as being within the native ranges of their host trees versus outside the native ranges of their host trees in the Methods (Lines 470-479). As a result, the number of pest species for which we could obtain reliable range information and thus assign a clear within or outside status is 1,875. Accordingly, we have updated the Abstract to state “1,875 pest species” instead of 2,038, and we have made the same correction in the manuscript.

3. Please justify splitting native and non-native pests in your analyses

Response: We revised our terminology and now distinguish between pests occurring within and outside the native ranges of their host trees. This host-centred classification is more ecologically meaningful than a simple pest-centred native versus non-native distinction. This separation reflects fundamental differences in ecological interactions. Within the native ranges of their host trees, pests and hosts have often undergone long-term co-evolution, which can result in host resistance and regulation by natural enemies. Although outbreaks can still occur under environmental stress (e.g., drought or habitat disturbance), pest impacts in these systems are often moderated by existing ecological controls. In contrast, when pests occur outside the native ranges of their host trees, host populations may lack evolved defenses and local natural enemies may not effectively regulate pest populations, increasing the likelihood of severe impacts. Distinguishing these two contexts is also relevant from a management perspective. Within host native ranges, management often focuses on mitigating environmental stressors and

maintaining ecosystem resilience, whereas outside host native ranges, prevention through biosecurity and phytosanitary measures becomes particularly important. In the revised manuscript, we have explicitly defined pests occurring within and outside their host native ranges (Lines 327-337) and added further explanation in Lines 468-476.

L327-337: “Our separation of pests within and outside host native ranges underscores how exposure maps can inform distinct management priorities. For pests occurring within host native ranges, exposure risk is strongly associated with timber import volume, highlighting the role of trade pathways in facilitating pest transport and redistribution among forest regions. In these cases, management should prioritize pathway management, including strengthened inspection of traded wood products, improved biosecurity along trade networks, and enhanced surveillance in major import hubs[62,78]. For pests occurring outside host native ranges, exposure risk is more constrained by ecological filtering, particularly host availability[63], suggesting that management strategies should focus on monitoring areas where suitable hosts are present or expanding, and on strengthening ecosystem resilience to reduce the likelihood of pest establishment[79].”

L468-476: “To characterize pest distributions, we followed the framework of Gougherty & Davies[90] and distinguished between pests occurring within and outside their host native ranges. This host-centred classification reflects the ecological dependence of forest pests on their hosts, as pest establishment and spread are strongly mediated by host availability and host-pest interactions[91]. We first compiled host associations for 2,038 forest pests and standardized host names using the “taxize” package[92]. Host geographic ranges were then compiled from Global Tree Search[93], the Global Naturalized Alien Flora database[94], CABI[87] and the World Checklist of Vascular Plants[95]. We integrated pest occurrence records with host geographic ranges to determine whether the pest occurred within or outside the native range of its host tree(s).”

Specific comments:

L163: "specialist pests can spread rapidly and cause large-scale mortality even when pest richness is low". So, pest richness alone is not a good enough proxy for forest mortality. Is this something that could also be focussed on?

Response: We agree that severe forest mortality can occur even when pest richness is low, particularly when outbreaks are driven by a small number of highly specialized or aggressive pest species. Our intention in the original sentence was to highlight this possibility rather than to imply that pest richness alone predicts forest mortality. Importantly, our study does not aim to estimate forest mortality directly. Instead, following the IPCC risk framework adopted in the revised manuscript, we use pest richness to represent the hazard component—that is, the diversity of potential pest threats present in a region. Forest ecosystem services (timber production, tree diversity, and carbon storage) represent exposure, and our objective is to identify areas where high hazard and high exposure spatially coincide. In this context, pest richness reflects the diversity of potential attack pathways rather than the expected severity of damage.

We fully acknowledge that the magnitude of forest mortality ultimately depends on additional factors such as pest abundance, outbreak dynamics, host susceptibility, and environmental stressors. As the Reviewers note, incorporating abundance or severity-weighted metrics would provide a more direct estimate of mortality risk. However, globally consistent datasets describing pest abundance or outbreak intensity across thousands of species are currently unavailable at the spatial scale required for this study.

To clarify this point, we have revised the text in Lines 72-79 as follows:

L72-79: "To provide a structured framework for this assessment, we adopt the IPCC Sixth Assessment Report (AR6) risk framework, which defines risk as hazard, exposure, and vulnerability[20]. Here, we conceptualize pest richness as the hazard component (i.e., the potential of pests to cause harm) and the spatial distributions of timber production, carbon storage, and tree biodiversity as the exposure component (i.e., the presence of valuable forest assets that may be affected). Accordingly, our analysis identifies potential risk zones (hazard-exposure overlap zones), characterized by the co-

occurrence of high pest richness and three critical forest ecosystem services."

We have also added a limitation statement in Lines 338-345:

L338-345: "Our analysis quantifies pest richness as a measure of hazard, rather than pest abundance or outbreak intensity. While abundance and outbreak severity more directly determine realized damage, globally consistent data on these metrics remain unavailable across taxa and regions[80]. Richness-based patterns therefore provide a practical first-order indicator for prioritizing surveillance at broad spatial scales. In this context, areas with higher pest richness may require greater monitoring attention because they contain a larger pool of taxa from which outbreaks may emerge, particularly where ecological regulation is weakened by host introduction, fragmentation, or climatic stress."

L167: Please clarify the contrast.

Response: The contrast is between the spatial association pattern observed in most regions (USA, Europe, etc.) versus that in China and Australia. Specifically, in most regions, timber-producing areas show a negative spatial correlation with flora pest richness (Moran's I < 0), indicating that simplified habitat heterogeneity in timber plantations does not support a wide array of host-specific flora pests. In contrast, China (Moran's I = 0.11, $p = 0.14$) and Australia (Moran's I = 0.32, $p < 0.01$) exhibit a positive spatial correlation. To make this contrast explicit, we have revised the sentence in the manuscript (Lines 205-208) as follows:

L205-208: "In contrast to regions where timber-producing areas show negative spatial associations with flora pest richness, timber-producing areas in China (Moran's I = 0.11, p = 0.14) and Australia (Moran's I = 0.32, p < 0.01) exhibit a positive spatial correlation with higher flora pest richness."

L168 (and elsewhere): please report the actual p-value (either up to 2 or 3 decimals, your choice), instead of using "< 0.05", or "not significant".

Response: We agree that reporting exact p-value enhances statistical transparency. Following the Reviewers' suggestion, we have revised the manuscript to provide exact p-value to two decimal places. For instances where p is less than 0.01, we have reported it as $p < 0.01$ in accordance with standard reporting conventions.

L175: same Moran's I as the previous values – is this a mistake?

Response: To avoid confusion, we have revised the relevant sentences to clearly differentiate the Moran's I values for each analysis in Lines 212-219 as follows:

L212-219: "Our spatial analysis revealed that flora pest richness had the strongest positive correlation with forest tree diversity in the USA (Moran's I = 0.23 for within host native range; $p < 0.01$), Australia (Moran's I = 0.14 and 0.12 for within and outside host native range, respectively; $p < 0.01$), and UK (Moran's I = 0.28 and 0.32 for within and outside host native range, respectively; $p < 0.01$). Correlations with potential carbon stock were strongest in the EU (Moran's I = 0.09 and 0.06 for within and outside host native range, respectively; $p < 0.01$), Australia (Moran's I = 0.11 for within host native range; $p < 0.01$) and China (Moran's I = 0.22 for within host native range; $p < 0.01$) (Fig. 3)."

L188: why are there two Moran's I values? These are repeated throughout the section – mistake?

Response: To avoid confusion, we have revised the relevant sentences to clearly differentiate the Moran's I values for each analysis in Lines 229-234 as below:

L229-234: "In addition to flora pests, regions with high forest tree diversity showed significant positive associations with fauna pest richness in most regions except for the UK (Moran's I = -0.07 and -0.14 for pests within and outside host native range, respectively; $p < 0.01$). Regions with potential carbon stock had the strongest

correlation with pathogenic pest richness in the UK (Moran's I = 0.12 and 0.08 for pests within and outside host native range, respectively; $p < 0.01$) (Fig. 3)."

L198: rephrase as 'pest richness increases with host tree diversity only'

Response: Done.

L212 (and elsewhere): there is no need to use predictor codes such as "bio_1". It would be best practice to use their actual names in the text and in Fig. 4 (see comment below on Fig. 4).

Response: We have replaced all predictor codes (e.g., "bio_1") with their corresponding full descriptive names (e.g., "mean temperature") throughout the main text, figures, and supplementary materials.

L243:The locations that require "high-resolution monitoring and rapid response strategies to prevent pest spillover threats" are large and hence, difficult to pinpoint exactly. However, we think this is beneficial information for forestry management regardless.

Response: We agree that the potential risk zones identified in our study are spatially extensive (covering millions of hectares at the global scale) and therefore cannot be directly translated into precise monitoring points. To address the Reviewer's concern, we have revised the sentence to avoid the implication that our maps alone can pinpoint exact locations for high-resolution monitoring. Instead, we now state that these potential risk zones represent priority areas for focusing monitoring and rapid response efforts, while also acknowledging that finer-scale, local data are needed for operational implementation. We have revised the text as follows (Lines 318-321):

L318-321: "Our study highlights that potential risk zones, where economically important forestry services coincide with high pest richness, should be prioritised for monitoring and rapid response efforts. While these zones are spatially extensive, they offer a valuable global blueprint for guiding resource allocation and regional-scale

management.”

L252: How much ‘more fragmented’?

Response: Based on the cited reference[16], we have revised the text to provide the exact percentage range reported as follows (Lines 348-350):

L348-350: “Between 51% and 67% of global forests became more fragmented from 2000 to 2020, suggesting that the vulnerability of forest carbon storage to pest outbreaks is escalating.”

L255: maybe offer advice on how to reduce fragmentation

Response: We have expanded the sentence to include specific measures for reducing fragmentation. The revised text now reads (Lines 350-355):

L350-355: “To ensure that increasing demand for forest services (e.g., timber, carbon storage) is met under a warming climate[81], reducing forest fragmentation and restoring forests will be essential. This can be achieved through measures such as establishing ecological corridors, limiting clear-cut size or adopting selective logging to reduce edge creation, protecting existing core forests, and restoring degraded forest lands[82].”

L268: surveillance data must be “standardized” – what does this mean? And why?

Response: By “standardized”, we mean that surveillance data should follow common data formatting, field definitions, taxonomic nomenclature, and metadata reporting protocols. This is essential for two reasons. First, predictive niche models require consistent input variables (e.g., occurrence coordinates, sampling dates, detection methods, host associations) across different databases and regions to avoid model biases. Second, early-warning platforms such as GBIF or national pest databases can only effectively aggregate and share data if all contributors use compatible data structures (e.g., Darwin Core standards). Without standardization, integrating heterogeneous datasets becomes error-prone, leading to large uncertainty in risk

forecasts. To make this clear, we have revised the sentence in Lines 364-371 as follows:

L364-371: "Building robust information systems and predictive models also necessitates widespread international cooperation. For effective identification and response, surveillance data that can be integrated with predictive niche models to forecast risks must follow common data standards (e.g., consistent field definitions, taxonomic nomenclature, and metadata reporting) and be shared on open-access platforms (e.g., GBIF, national pest databases). Such standardization is critical because predictive models require harmonized inputs across regions and databases, and early-warning systems can only aggregate data effectively when all contributors use compatible formats (e.g., Darwin Core standards)[84]."

L275: define author name abbreviations

Response: We have replaced all abbreviations with full author names for the first time.

L281: should J.L also appear in line 275 instead of J.Q.L?

Response: We have standardized the abbreviation for Jianquan Liu (J.Q.L.) in the manuscript.

L289: Is (a,b) all pests? Put letters before their description.

Response: Sub-figures (a) and (b) both refer to all forest pests (overall richness and scaled richness comparing within and outside the native ranges of host trees, respectively). We have now restructured the legend to place the letter labels directly before their corresponding descriptions, as shown below:

"**Fig. 1 Global spatial pattern of forest pest richness. (a, b)** All forest pests with **(a)** overall richness and **(b)** scaled richness ranging from -1 to +1 comparing the relative difference in pest richness within and outside the native ranges of host trees (positive values indicate higher pest richness within host native ranges). **(c, d)** Fauna**.**

(e, f) Flora**. (g, h)** Pathogenic forest pests. Circle plots in the bottom-left indicate the proportion of each pest group among all forest pests. The color bar indicates the species richness of each pixel, with warmer colors representing higher values. To improve display, all maps have been resampled to 1° resolution."

L301: Is the reddish-orange colour for diversity and carbon combined? Other descriptions do not match the colour legend

Response: In the revised manuscript, we have completely rewritten the color legend as follows:

"**Fig. 2 Global potential risk zones exposed to forest pests across different ecosystem services.** Percentages of total exposed land area of timber production, forest tree diversity, and potential carbon stock across different types of potential risk zones exposed to forest pests **(a)** within and **(b)** outside host native ranges. The orange, blue, and green colors represent potential risk zones exposed to forest pests across a single ecosystem service; the yellow, dark orange, and deep blue represent potential risk zones exposed to forest pests across pairs of ecosystem services; and the pink represents the intersection of all three ecosystem services. The bar charts represent the composition of exposed area for the countries/regions with the largest exposed zones **(c)** within and **(d)** outside host native ranges, and the numbers above the bars indicate the total area exposed. The scatter point charts show the proportion of potential risk zones relative to total forest area across countries/regions **(e)** within and **(f)** outside host native ranges."

L343: The GBIF abbreviation is not defined.

Response: We have ensured that GBIF is defined upon its first use in the manuscript (Line 462).

L373-374: which ML models?

Response: In the original study by Liang et al.[17], the rarefaction-based normalization

was implemented using random forest models. We have revised the text to state this explicitly, as shown (Lines 494-495):

L494-495: "Plot-level species richness (α-diversity) was normalized to 1-ha area using rarefaction-based random forests models, accounting for sampling design heterogeneity."

L384: How did you choose the four 'Bioclim' variables from the 19 options? What were the criteria?

Response: We calculated pairwise Pearson correlation coefficients among all 19 Bioclim variables across global forest grid cells. To reduce collinearity, we removed variables with $|r| > 0.9$, retaining those with stronger ecological interpretability for pest distribution. This process yielded mean temperature (BIO1), isothermality (BIO3), and mean precipitation (BIO12). We acknowledge that random forest models are generally robust to multicollinearity and do not strictly require variable selection. However, we chose to reduce the variables for two additional reasons: (1) a smaller set of ecologically meaningful predictors improves interpretability and reduces the risk of overfitting to noise, especially given the global scale and spatial autocorrelation in our data; (2) reducing the number of climate variables speeds up the repeated cross-validation steps without materially affecting predictive performance. To improve the clarity of the Methods section, we have restructured the text to separate data sources from analytical procedures. Specifically, we moved the rationale and process for variable selection to the "Statistical Analysis" subsection (Lines 593-601) and as below, while the "Other data" subsection now focuses exclusively on the characteristics and sources of the datasets used.

L593-601: "The response variable was the presence of potential risk zones identified from the spatial overlap between pest richness and ecosystem services. Predictor variables included environmental, landscape, and socioeconomic factors. Although Random Forests are inherently robust to multicollinearity, we removed several highly

correlated climate predictors (*r* > *0.9*) to improve computational efficiency and model interpretability. Nine variables were retained for final model construction, including mean temperature (BIO1), isothermality (BIO3), mean precipitation (BIO12), forest age, forest type, GNI, timber import volume, population density, and travel time."

L401: Could you please clarify what exactly is being used to calculate the proportion?

Response: The proportion is defined as the total area of potential risk zones within a country divided by the total forest area of that country. We have revised the sentence in the Methods section (Lines 521-523) to state this explicitly:

L521-523: "To calculate the proportion of potential risk zones relative to total forest area for each country, we divided the total area of potential risk zones by the total forest cover within that country[112]."

L410: "To focus the species occurrence, we retained only one unique observation point for each species within each 10 km grid cell". Here, it seems that you have reduced species occurrence from the beginning, which may impact your results. Can you say that your risk zone percentages are precise and correct?

Response: The step of retaining only one occurrence record per species within each 10 km grid cell was implemented to reduce spatial sampling bias and avoid duplicate records representing the same local presence. Large global biodiversity databases often contain multiple observations of the same species within a single grid cell due to repeated surveys, multiple reporting sources, or duplicated database entries. Without filtering, such clustering of records can inflate sampling intensity without providing additional information on species presence.

Importantly, our analysis is based on species presence within each grid cell rather than the number of occurrence records. Therefore, retaining a single record per species per grid cell removes redundant observations while preserving the presence of that species in the cell. As a result, this filtering step does not change the calculated species richness for any grid cell, but simply standardizes the occurrence dataset used to derive

the richness map. This type of spatial deduplication is a common preprocessing step in large-scale biodiversity analyses to reduce sampling bias while maintaining species distribution patterns. Because pest richness is calculated from unique species presences per grid cell, this procedure does not influence the estimation of pest richness or the resulting proportions of identified risk zones. To clarify this methodological step, we have revised the description in the Methods section (Lines 532–535):

L532-535: "To reduce spatial sampling bias and avoid multiple records representing the same local occurrence, we retained only one unique observation point for each species within each 10-km grid cell[113]."

L433: k is referred to as the "neighbourhood" and the "spatial weight". Please clarify.

Response: In the original text, the term "spatial weight" was used to refer to the neighbourhood size parameter (k) in the k-nearest-neighbour graph. In spatial autocorrelation analyses, k defines the neighbourhood structure from which the spatial weights matrix is constructed, rather than representing the spatial weights themselves. We have revised the sentence in the Methods section (Lines 580-583) to clarify this distinction as below:

L580-583: "We determined the optimal neighbourhood size by iteratively constructing k-nearest-neighbour graphs with k ranging from 1 to 20 and selecting the value of k that maximized the global bivariate Moran's I statistic."

L434: which statistic?

Response: The "observed statistic" refers to the global bivariate Moran's I value. The revised sentence now states this in the Method section (Lines 580-583):

L580-583: "We determined the optimal neighbourhood size by iteratively constructing k-nearest-neighbour graphs with k ranging from 1 to 20 and selecting the value of k that maximized the global bivariate Moran's I statistic."

L436: why did you use 499 permutations?

Response: The use of 499 permutations follows common practice in spatial autocorrelation analyses based on Monte Carlo permutation tests. This number of permutations provides a stable approximation of the null distribution while maintaining reasonable computational efficiency. With 499 permutations, the smallest attainable pseudo p-value is 1/(499+1) = 0.002, which is sufficient for detecting statistically significant spatial autocorrelation at significance levels ($\alpha = 0.05$). Similar permutation numbers have been widely used in spatial ecological studies employing Moran's I. We have clarified this in the Methods section (Lines 585-587):

L585-587: "We then applied the global bivariate Moran's I test with 499 random permutations to assess statistical significance by comparing the observed Moran's I with a permutation-based null distribution."

L438: which confidence level of the three listed did you use to assess significance?

Response: In our analysis, statistical significance was assessed based on the 95% bootstrap confidence interval. We have revised the Methods section (Lines 587-590) to clarify this:

L587-590: "Finally, we generated bootstrap confidence intervals at the 95% level to quantify uncertainty. Statistical significance was assessed based on whether the 95% confidence interval excluded zero for the spatial association between pest richness and ecosystem services."

L441-453: what are the predictors you included in your RF model? What was the response variable?

Response: In the revised manuscript, we have explicitly described the model structure. The response variable in the RF model was the presence of potential risk zones identified from the spatial overlap between pest richness and ecosystem services.

Predictor variables included environmental, landscape, and socioeconomic factors. We have revised the Methods section (Lines 593-601) to clarify both the response variable and the predictors used in the model:

L593-601: "The response variable was the presence of potential risk zones identified from the spatial overlap between pest richness and ecosystem services. Predictor variables included environmental, landscape, and socioeconomic factors. Although Random Forests are inherently robust to multicollinearity, we removed several highly correlated climate predictors ($r > 0.9$) to improve computational efficiency and model interpretability. Nine variables were retained for final model construction, including mean temperature (BIO1), isothermality (BIO3), mean precipitation (BIO12), forest age, forest type, GNI, timber import volume, population density, and travel time."

L465: is this an imbalanced classification setting?

Response: The classification of potential risk zones resulted in an imbalanced dataset, with substantially fewer potential risk grid cells than non-risk cells. To reduce potential bias during model training, the majority class was downsampled within each spatial cross-validation fold. Model performance was evaluated using Cohen's Kappa statistic, which measures the agreement between predicted and observed classifications. We have clarified this in the Methods section (Lines 617-622) as follows:

L617-622: "Given the imbalanced dataset between potential risk and non-risk cells, the majority class was downsampled within each spatial cross-validation fold to reduce potential bias during model training. The final model was retrained on the full dataset, and permutation-based variable importance scores were extracted. To evaluate model performance, we used Cohen's Kappa statistic, which measures the agreement between predicted and observed classifications."

L488: the hyperlinked page is not the same as the text

Response: The GitHub repository linked in the manuscript was temporarily out of date

because the original code files were accidentally overwritten during an update. We have now completely restored and reorganised the repository with detailed README explaining how to run the analysis step by step. The repository URL remains the same: https://github.com/bobilong/forest_pests.

Comments about figures:

Fig. 1: There are a few problems with this display.

- First, the current caption renders this figure completely unintelligible. It does not explain the differences between the left and right panels.

Response: In the revised manuscript, we have substantially rewritten the caption to explicitly explain the differences between the left and right panels. The revised caption now reads as follows:

"**Fig. 1 Global spatial pattern of forest pest richness. (a, b)** All forest pests with **(a)** overall richness and **(b)** scaled richness ranging from -1 to +1 comparing the relative difference in pest richness within and outside the native ranges of host trees (positive values indicate higher pest richness within host native ranges). **(c, d)** Fauna**. (e, f)** Flora**. (g, h)** Pathogenic forest pests. Circle plots in the bottom-left indicate the proportion of each pest group among all forest pests. The color bar indicates the species richness of each pixel, with warmer colors representing higher values. To improve display, all maps have been resampled to 1° resolution."

- Also, the caption states yellow and red highlights, which are not present in the figure. The resolution makes it very difficult to see the actual patterns being displayed.

Response: We have now removed this misleading phrase from the caption. To improve visual clarity while preserving global patterns, we have resampled all maps to 1° resolution for display purposes, as already noted in the revised caption. We have updated the caption accordingly and replaced the figure with the resampled version.

- What are the circles at the bottom-left of panels c, e and g?

Response: The circles at the bottom-left of panels (c), (e), and (g) indicate the relative proportion of each pest functional group (fauna, flora, and pathogenic pests) among all forest pests in our dataset. In the revised caption, we have now explicitly described the meaning of these circles. The meaning of these circles has been added to the Figure 1 caption, as shown in our response above.

- The divergent colour palette chosen includes gray/white in the centre; this makes it impossible to distinguish from the actual gray/white background of the world map.

Response: We have revised the colour palette to improve contrast between the data layer and the map background.

- What does the scale non-native to native, going from -1 to 1, represent? How is it calculated?

Response: Following the Reviewers' suggestions, we have redefined the terminology in the revised manuscript. We now distinguish between pest within and outside the native ranges of their host trees. The scale from -1 to +1 in Figure 1 (panels b, d, f, h) represents the standardised difference in pest richness between these two groups for each grid cell. Specifically, we calculated:

$$Scaled\ richness = \frac{R_{within} - R_{outside}}{R_{within} + R_{outside}}$$

where R_{within} and $R_{outside}$ denote pest richness within and outside the native ranges of host trees, respectively. The positive values indicate higher richness within host ranges, negative values indicate higher richness outside host ranges, and values near zero indicate similar richness between the two. We have clarified figure caption, as shown in our response above.

- use a different colour scale for (b), (d), (f), and (h)

Response: We have revised the figure to use a distinct colour scale for these panels to

better distinguish them from the richness maps and improve visual clarity.

- how do you interpret panels (b), (d), (f), and (h)?

Response: Panels (b), (d), (f), and (h) show the scaled difference between pest richness within and outside the native ranges of their host trees. Positive values indicate higher pest richness within host ranges, whereas negative values indicate higher richness outside host ranges, and values near zero indicate similar richness between the two. We have clarified the interpretation of these panels in the revised figure caption, as shown in our response above.

Fig. 2: panels a and b could use the Venn diagram-style displays similarly to Suppl.

Response: Following the Reviewers' recommendation, we have revised Fig. 2a and 2b to use Venn diagram-style displays, consistent with those used in the Supplementary Figures. The updated panels are shown in the revised Fig. 2 as below:

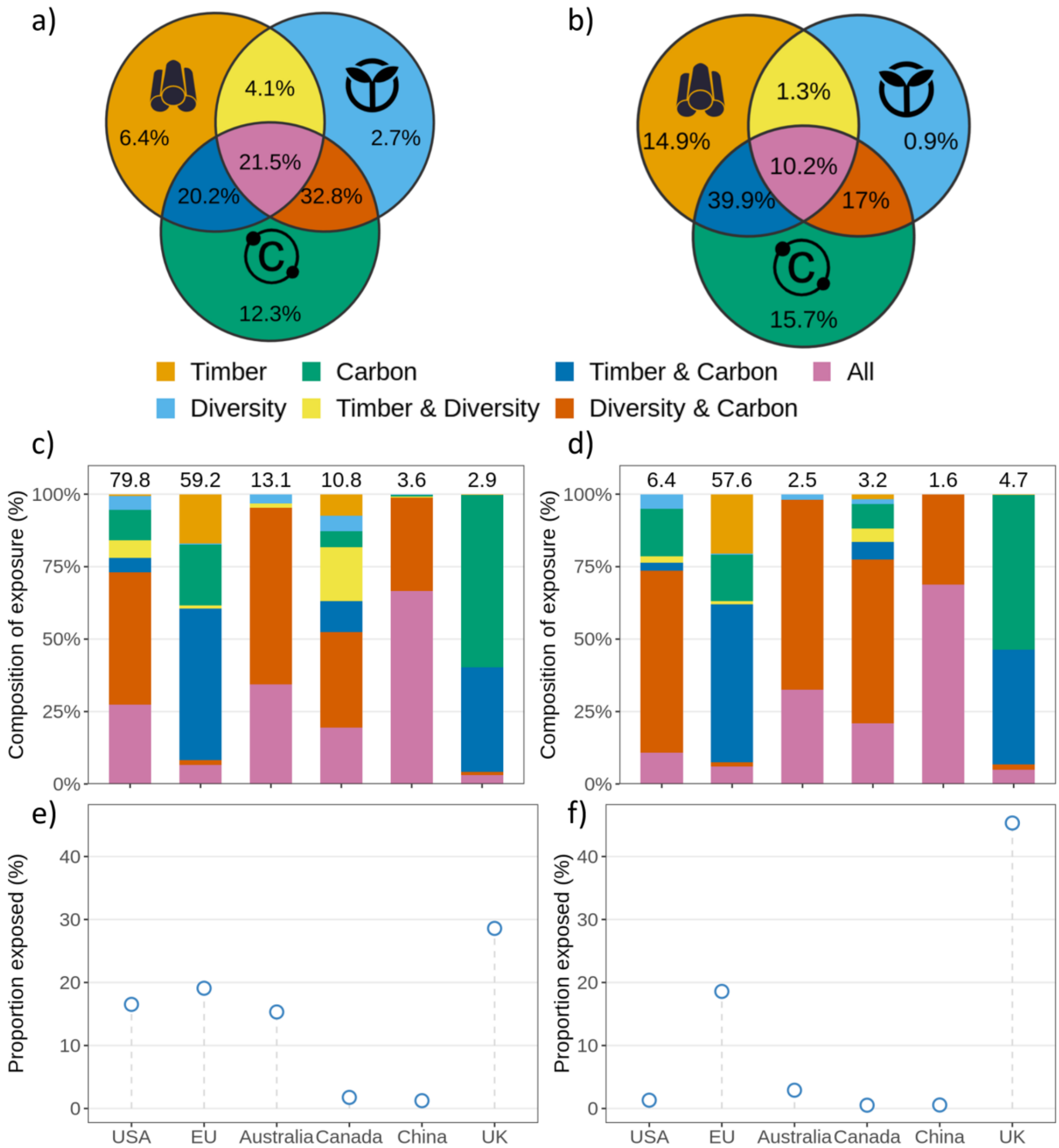

Fig. 2 Global potential risk zones exposed to forest pests across different ecosystem services. Percentages of total exposed land area of timber production, forest tree diversity, and potential carbon stock across different types of potential risk zones exposed to forest pests **(a)** within and **(b)** outside host native ranges. The orange, blue, and green colors represent potential risk zones exposed to forest pests across a single ecosystem service; the yellow, dark orange, and deep blue represent potential risk zones exposed to forest pests across pairs of ecosystem services; and the pink represents the intersection of all three ecosystem services. The bar charts represent the composition of exposed area for the countries/regions with the largest exposed zones **(c)** within and **(d)** outside host native ranges, and the numbers above the bars indicate the total area

exposed. The scatter point charts show the proportion of potential risk zones relative to total forest area across countries/regions **(e)** within and **(f)** outside host native ranges.

Fig. 2. Currently it is difficult to distinguish all the colours quickly. Panels c and d should have a standardised y-axis range to allow for comparisons (both the left and right y-axis ranges), and preferably the same ordering in the x-axis as well. Moreover, in panels c and d, you are connecting the dots as if the continents/countries are a continuous variable, which is not appropriate for a categorical variable. Also, the y-axes are not comparable.

Response: We have substantially revised Fig. 2c and 2d to improve clarity and comparability. The original panels 2c and 2d have been reorganised into four panels (Fig. 2c-f). Panels 2c and 2d now show the total area and proportional coverage of different exposure types across countries, while panels 2e and 2f show the proportion of potential risk zones relative to the total forest area in each country. This redesign improves the distinction among colours. We standard the y-axis ranges to allow direct comparisons across panels, and ensure a consistent ordering of countries along the x-axis. In addition, the connecting lines between points have been removed, as countries represent categorical variables.

Fig. 3: A few issues here as well.

- The caption needs proofreading.

Response: The caption of Fig. 3 has been carefully proofread and revised to improve clarity and wording in the revised manuscript as below:

"**Fig. 3 Spatial correlation between forest pest functional groups and ecosystem services across main exposure regions**. **(a)** The most spatially correlated forest pest functional groups with ecosystem services across the main exposure regions. Circular diagrams indicate the pest-service combinations with the highest global bivariate Moran's I values. The legend with grey color (light grey, pest within host native range; dark grey, pest outside host native range) indicates the three ecosystem services. **(b)** Heatmaps of global bivariate Moran's I values for all combinations of pest

functional groups and ecosystem services. A warmer color represents a clustered pattern for forest pests and ecosystem services and a cooler colour represents a dispersed pattern. A value tending to 0 represents a random pattern. Asterisks denote significance levels (p < *0.05**, p < *0.01***). Details on these results are given in Supplementary Table 1."

- Why present only the group with the highest spatial correlation? What if there is another group with a very similar spatial correlation, just slightly smaller numerically? That would be of interest too. It would be preferable to show all Moran indices using a heatmap, for instance.

Response: We thank the reviewer for the valuable suggestion. In the revised manuscript, we have retained the original panel highlighting the forest pest functional groups with the highest spatial correlation with ecosystem services, as this provides a clear focus on the strongest associations. In addition, we have added a new panel (Fig. 3b) showing a heatmap of all global bivariate Moran'I across all forest pest functional groups and ecosystem services as below:

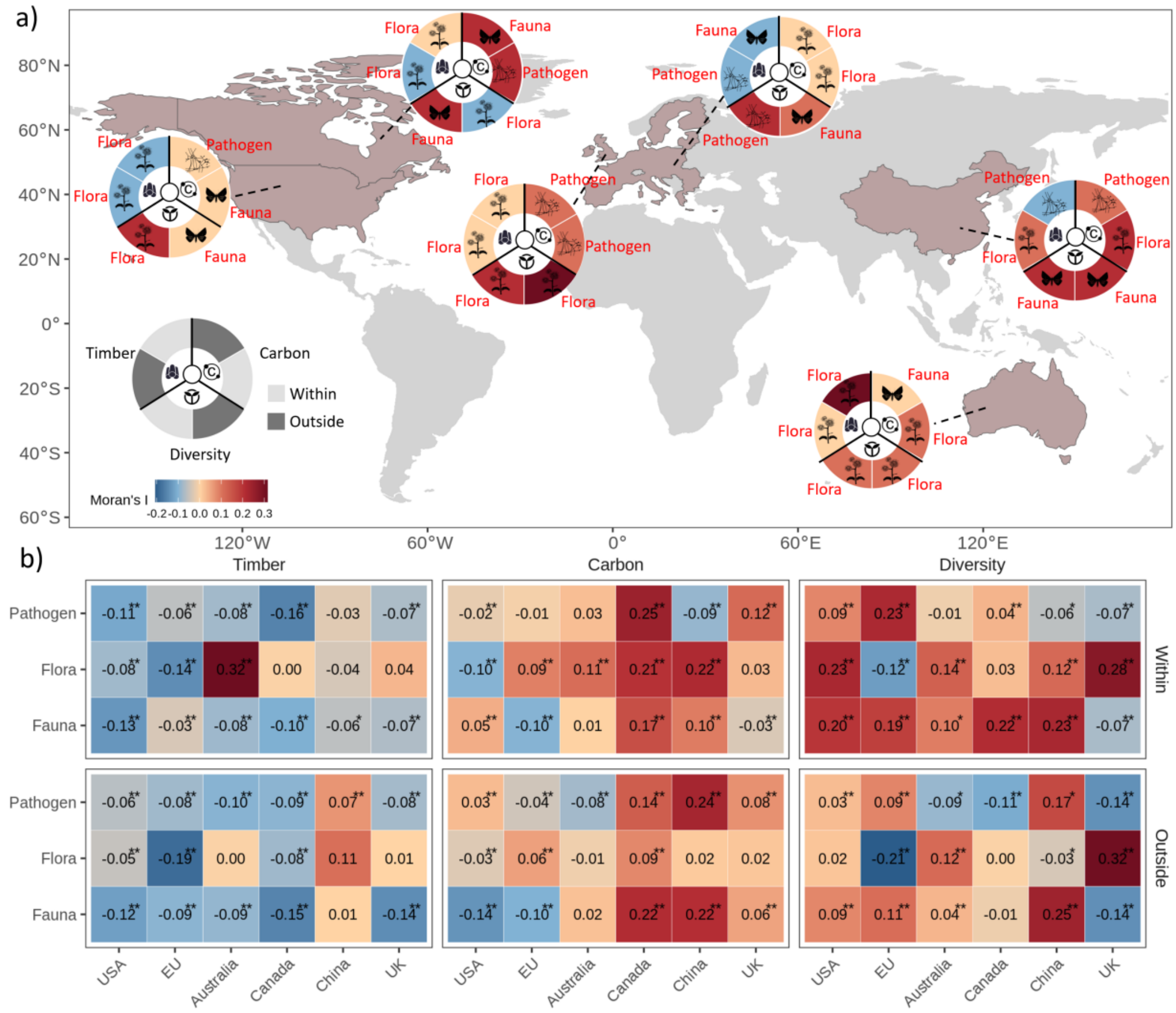

Fig. 3 Spatial correlation between forest pest functional groups and ecosystem services across main exposure regions. **(a)** The most spatially correlated forest pest functional groups with ecosystem services across the main exposure regions. Circle plots indicate the pest-service combinations with the highest global bivariate Moran's I values. The legend with grey color (light grey, pest within host native range; dark grey, pest outside host native range) indicates the three ecosystem services. **(b)** Heatmaps of global bivariate Moran's I values for all combinations of pest functional groups and ecosystem services. A warmer color represents a clustered pattern for forest pests and ecosystem services and a cooler colour represents a dispersed pattern. A value tending to 0 represents a random pattern. Asterisks denote significance levels (*p < 0.05**, *p < 0.01***). Details on these results are given in Supplementary Table 1.

- How do you interpret the grey wheel? What do wheel positions tell you? Some of the pest group diagrams are in a bolder font than others – is this intentional? So in the USA,

native flora are clustered in carbon stock forests? What does this actually mean? Provide a sample interpretation in the figure caption

Response: We thank the Reviewer for these critical questions. Regarding the grey wheel (legend), we have redesigned it to serve as a clear hierarchical spatial key. The wheel is partitioned into three primary sectors representing the ecosystem services (Timber, Carbon, and Diversity), with each sector subdivided by shading—light grey consistently represents pest within host ranges and dark grey represents pest outside host ranges. We have now standardized all fonts for consistency.

As the reviewer correctly interpreted, the clustering of native flora pests within carbon stock forests in the USA indicates a strong spatial overlap between high-value carbon areas and high richness of flora pests. To ensure these points are clear to all readers, we have revised the figure caption to include a sample interpretation, as shown in our response above.

Fig. 4: please use the actual predictor names instead of “bio_1”, “bio_12” etc. Change the ‘Outside’ label to ‘Non-native’. How are there multiple SHAP values per biological variable? What do low and high feature values mean?

Response: Thank you for the suggestion. The variables “bio_1”, “bio_12”, and “bio_3” refer to standard bioclimatic predictors derived from the WorldClim dataset. To improve clarity and readability, we have replaced these abbreviations with their full names in Fig. 4. Specifically, bio_1, bio_12, and bio_3 are now labelled as mean Temperature, mean precipitation, and isothermality, respectively. Following reviewer’s comments, we have adopted the terminology “within host native range” and “outside host native range” (as defined in the Methods and used consistently in all figures and text). For brevity within the figure panels, we use “Within” and “Outside”, and we clearly define these terms in the figure caption.

Each point in the SHAP plots represents the SHAP value of a predictor for an individual observation in the dataset. Therefore, multiple SHAP values appear for each predictor because the SHAP value is calculated for every sample used in the random forest model. The violin distributions summarize the variability of the SHAP values

across all observations, illustrating how each predictor contributes to the model predictions. In the SHAP summary plots, the color of each point represents the relative magnitude of the feature value for that predictor. Blue indicates low values of the feature, whereas red indicates high values. This color gradient allows visualization of how different magnitudes of a predictor influence the SHAP value and thus the model prediction. We have revised both caption of Fig. 4 to clarify the SHAP value plots as below:

"**Fig. 4 Factors influencing high risk zones exposed to forest pests among different ecosystem services.** The importance of each feature based on Gini coefficients and the mean absolute value of SHAP for potential risk zones of **(a)** timber-producing areas, **(d)** forest tree diversity and **(g)** potential carbon stock exposed to forest pests within and outside host native ranges. Distribution of SHAP values based on the random forest model among **(b, c)** timber-producing area, **(e, f)** forest tree diversity and **(h, i)** potential carbon stock within and outside host native ranges of forest pests, respectively. Each point represents the SHAP value for an individual observation, and the color gradient (blue to red) indicates low to high feature values."

Suppl. Fig. 1: typo: "defination" should be "definition" and "postive" should be "positive". Also, the caption is incomplete, please include a more descriptive caption.

Response: We have corrected "defination" to "definition" and "postive" to "positive" in the revised figure. Additionally, we have expanded the figure caption to provide a more comprehensive and descriptive overview of the species filtering pipeline as below:

"**Supplementary Fig. 1. Workflow for the identification and filtering of the global forest pest**. The flowchart shows the stepwise procedure used to compile the final forest pest dataset based on the FAO definition of forest pest. Forest pests were

determined using three criteria including species reported to affect forests, species distributed in forest habitats, and species whose hosts are trees. Species with negative impacts on trees or forests were retained, and the resulting records were further checked manually to remove uncertain or misclassified cases, yielding a final dataset of 2,038 forest pests."

Suppl. Fig. 3: why not use a gradient of colours throughout all plots, from top 1% to 99%, for instance? Currently the top 5% and 15% represent a very small portion of the maps and it is very difficult to see.

Response: We have revised Supplementary Fig. 3 to use a continuous colour gradient based on percentile ranking of values across all grid cells:

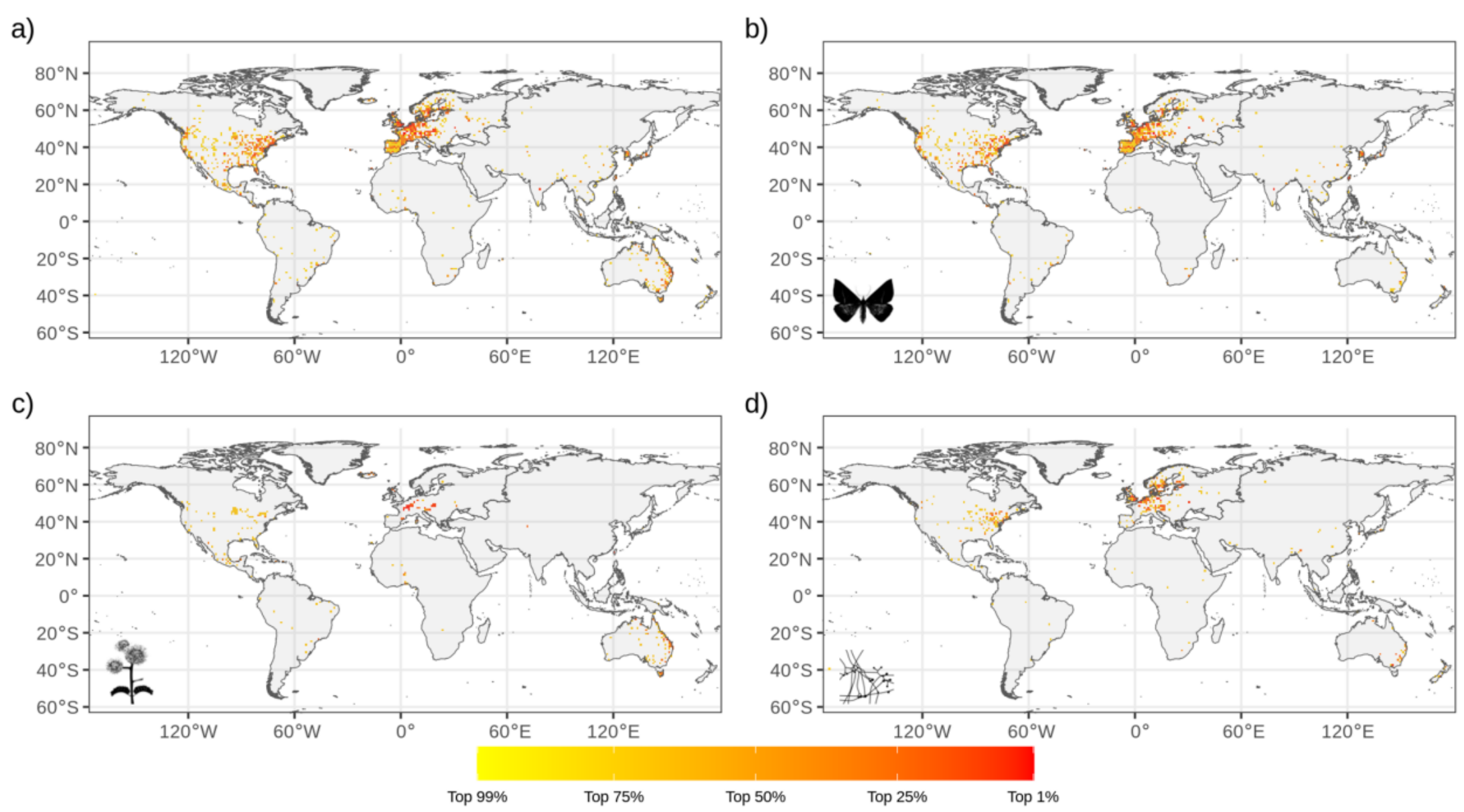

Supplementary Fig. 3 Global spatial pattern of forest pest richness. Spatial hotspots across **(a)** all, **(b)** fauna,**(c)** flora, and **(d)** pathogen forest pests. Colours represent the relative ranking of pest richness across grid cells expressed as percentiles, where higher values (top 1%) indicate greater richness and lower values (top 99%) indicate lower richness, displayed using a continuous yellow-to-red gradient. To improve display, all maps have been resampled to 1° resolution.

Suppl. Fig. 4: this display is worse than Suppl. Fig. 3 in terms of visualisation of the actual patterns. I would recommend perhaps decreasing the resolution? This way the squares would be slightly larger and could improve visualisation. Currently, there are 7 colours in the key, but it is very difficult to distinguish them.

Response: To improve the overall flow of the manuscript, we have integrated the Venn diagram-based analysis into Figure 2 (panels a and b) in the main text. This restructuring allows us to more effectively display the overlap between pest richness and ecosystem services using the clear, intuitive Venn-style visualization suggested by the reviewer. Consequently, the original Supplementary Figure 4 has been removed to avoid redundancy. We have also ensured that the color palette and resolution in the revised Figure 2 are clear.

Suppl. Fig. 6: the y-axis ranges should be standardises, at least across (a-d), (b-e) and (c-f).

Response: We have revised the figure (now Supplementary Fig. 7; previously Supplementary Fig. 6) by standardising the y-axis ranges within each ecosystem service category, specifically across panels (a-d), (b-e), and (c-f).

Suppl. Figs. 8, 9 and 10: captions need English revision. It also states a red curve, but the curve in the figure is yellow, not red. Panel (f) displays forest type as a continuous predictor, but that is a categorical variable. Also, having the curves the same colour as the points makes visualisation more difficult. You could use darker shades for the curves, instead.

Response: We have carefully revised the captions of Supplementary Figs. 8, 9, and 10 to improve the clarity. The description of the curve color has also been corrected to match the figure. In addition, we revised panel (j) to properly represent forest type as a categorical variable. Finally, the visualization has been improved by using darker shades for the curves to distinguish them more clearly from the scatter points

Suppl. Table 1: please inform the actual p-values rather than stating "< 0.05" or "not significant". The p-values stated as "< 0.01" are fine.

Response: Following the Reviewers' suggestion, we have revised the manuscript to provide exact p-value to two decimal places. For instances where p is less than 0.01, we have reported it as $p < 0.01$ in accordance with standard reporting conventions.

Typos/punctuation/recommended corrections:

Line 93: 'of records'

Response: Done.

Line 142: 'impact of'

Response: We have revised the text accordingly.

Line 173: change 'was' to 'had the', 'EU' to 'the EU'

Response: We have revised the text accordingly.

Line 177: 'established' instead of 'establish'

Response: We have revised the text accordingly.

Line 179: comma after 'where abundant'

Response: We have revised the text accordingly.

Line 180: 'the' before 'resource'

Response: We have revised the text accordingly.

Line 186: 'respectively' after '0.26'

Response: We have revised the text accordingly.

Line 187: remove 'While', change 'were most strongest' to 'had the strongest correlation'

Response: We have revised the text accordingly.

Line 191: ‘a’ before ‘previous’

Response: We have revised the text accordingly.

Line 205: ‘control for’

Response: We have revised the text accordingly.

Line 219: ‘a’ before ‘non-linear effect’

Response: We have revised the text accordingly.

Line 263: ‘unmanned aerial vehicles’?

Response: Done

Line 276: change ‘works were’ to ‘work was’

Response: We have revised the text accordingly.

Line 290: ‘pixel’ instead of ‘pixels’, ‘values’ instead of ‘value’

Response: We have revised the text accordingly.

Line 301: ‘intersection of’?

Response: Done

Line 303: ‘the’ before ‘top’

Response: We have revised the text accordingly.

Lines 308-310: change ‘ The warmer color represents the pattern of forest pests and ecosystem services is clustered, and colder color represents that is dispersed’ to ‘A warmer color represents a clustered pattern for forest pests and ecosystem services and a cooler colour represents a dispersed pattern’.

Response: We have revised the text accordingly.

Line 310: change 'Where the value tends to 0 represents random pattern' to 'a value tending to 0 represents a random pattern'

Response: We have revised the text accordingly.

Line 315: change ',' to 'for', 'area' to 'areas'

Response: We have revised the text accordingly.

Line 317: change 'SHAP values distribution' to the 'distribution of SHAP values', 'area' to 'areas'

Response: We have revised the text accordingly.

Line 327: 'and' after ','

Response: We have revised the text accordingly.

Line 328: change 'encompassing' to 'encompasses'

Response: We have revised the text accordingly.

Line 338: change 'first' to 'firstly'

Response: We have revised the text accordingly.

Line 340: change 'fuzzy' to 'unclear'

Response: We have revised the text accordingly.

Line 341: change 'second' to 'secondly'

Response: We have revised the text accordingly.

Line 342: change ‘third’ to ‘thirdly’

Response: We have revised the text accordingly.

Line 344: change ‘fourth’ to ‘fourthly’

Response: We have revised the text accordingly.

Line 345: change ‘pests’ to ‘pest’

Response: We have revised the text accordingly.

Line 365: developed – used?

Response: We have revised the text accordingly.

Line 366: change ‘categorizing’ to ‘to categorize’

Response: We have revised the text accordingly.

Line 378-380: put ‘the’ in front of each named map

Response: We have revised the text accordingly.

Line 386: ‘divided by’ instead of ‘divide’

Response: We have revised the text accordingly.

Line 389: ‘.’ instead of ‘,’. ‘as’ instead of ‘to’

Response: We have revised the text accordingly.

Line 390: ‘of’ before ‘at’

Response: We have revised the text accordingly.

Line 391: ‘pests’ instead of ‘pest’

Response: We have revised the text accordingly.

Line 394: ‘.’ instead of ‘,’

Response: We have revised the text accordingly.

Line 395: ‘identify’ instead of ‘identifies’

Response: We have revised the text accordingly.

Line 396: MODIS abbreviation not defined. ‘the’ before ‘MODIS’

Response: The full definition of MODIS has been added, and ‘the’ added

Line 397: ‘following; instead of ‘follows’

Response: We have revised the text accordingly.

Line 404: ‘the’ before GBIF

Response: We have revised the text accordingly.

Line 407: ‘standards’ instead of ‘standard’

Response: We have revised the text accordingly.

Line 408: ‘at’ before ‘which’

Response: We have revised the text accordingly.

Line 409: ‘the’ before ‘occurrence’, ‘as’ instead of ‘because’

Response: We have revised the text accordingly.

Line 411: ‘Correspondingly’ instead of ‘Corresponding’

Response: We have revised the text accordingly.

Line 412: ‘removed’ instead of ‘filtered out’

Response: We have revised the text accordingly.

Line 418: ‘combine’ instead of ‘overlap’? ‘the’ before ‘richness’

Response: We have revised the text accordingly.

Line 420: ‘areas’ instead of ‘area’

Response: We have revised the text accordingly.

Line 424: ‘ranges’ instead of ‘range’

Response: We have revised the text accordingly.

Line 426: ‘to’ before ‘total’, ‘coverage’ instead of ‘cover’?

Response: We have revised the text accordingly.

Line 432: ‘disperse’ instead of ‘dispersed’

Response: We have revised the text accordingly.

Line 435: ‘one’ instead of ‘unity’

Response: We have revised the text accordingly.

Line 441: ‘the’ before ‘”ranger”’

Response: We have revised the text accordingly.

Line 442: ‘the’ before ‘”fastshap”’

Response: We have revised the text accordingly.

Line 444: ‘an’ before ‘ensemble’

Response: We have revised the text accordingly.

Line 446: ‘offers’ instead of ‘offer’, change ‘reflects importance of variable in predicting power within the model’ to ‘reflects the predictive power of a variable in the

model'

Response: We have revised the text accordingly.

Line 451: change 'Random Forests' to 'RFs'?

Response: We have revised the text accordingly.

Line 455: 'with partitions the data into three spatial folds' to 'with three spatial fold partitions in the data'

Response: We have revised the text accordingly.

Line 457: 'the' before '"blockCV"', change comma to colon (:)

Response: We have revised the text accordingly.

Line 459: change 'the adjust' to 'adjusted'

Response: We have revised the text accordingly.

Line 466: 'simulations' instead of 'repeats'?

Response: We have revised the text accordingly.

Reviewer #3 (Remarks to the Author):

I co-reviewed this manuscript with one of the reviewers who provided the listed reports. This is part of the Nature Communications initiative to facilitate training in peer review and to provide appropriate recognition for Early Career Researchers who co-review manuscripts.

#################Part B: Response to manuscript annotations#################

The following comments were provided as inline annotations on the manuscript by Reviewer. We have addressed each comment below:

Lines 32-33 The authors should define here what a pest is (if defined as FAO in the

Suppl. figure 1, state it here). It doesn't seem that, in this paper, pests are only pathogenic microbes, but also insects.

Response: In the revised manuscript, we now explicitly define forest pests following the definition of the FAO. In our study, pests include not only pathogenic microbes but also other harmful organisms such as insects and parasitic plants that damage trees. This clarification has been added to the main text in Lines 33-37 as follows:

L33-37: "However, these services are increasingly jeopardized by outbreaks of pests—any living organism that is injurious to plants or plant products[3]. In forest ecosystems, these pests include insects, pathogenic microbes, parasitic plants and any other harmful organisms that cause physiological and structural damage to trees[3]."

Lines 44-45 The citations do not seem to support the statement, as they deal with climate warming an drought-induced amplified damage by pests, instead of global trade as a driver of pest invasions. Please, modify either the statement or the citations.

On a more general note, the paragraph addresses points that are not well connected: global warming, habitat degradation, global trade. It is not easy to get the point. Please, think of reframing it.

Response: We agree that the original paragraph presented multiple drivers (climate warming, habitat fragmentation, global trade) in a way that lacked clear logical flow, and that the citations at the end of the sentence did not specifically support the global trade statement. We have substantially reframed the paragraph (Lines 42-54) to improve the logical flow thus:

L42-54: "The escalating global threat from forest pests reflects the combined effects of climate change, landscape modification, and international trade. Climate warming and more frequent extreme droughts weaken tree resistance, extend pest overwintering ranges, and enhance pesticide resistance[8,9], exacerbating pest spread. In Europe, the overlap between forest and pest habitats is projected to increase by 51% under medium-to-high emissions scenarios[10]. These climatic shifts are compounded by human-

mediated dispersal. Global trade is a primary vector for such introductions as exemplified, for example, by the flight-limited European spongy moth, which causes extensive damage in Canada—its spread accelerated by the transport of infested wood materials[11]. Once introduced, the establishment and rapid colonization of pests are further facilitated by landscape modification because of human disturbances to natural environments. Habitat degradation and fragmentation[12], combined with the uniform genetic composition and closely spaced trees of monoculture plantations, provide easily accessible food for pests, leading to rapid colonization[13]."

Line 48 The chestnut blight is tha disease, which is caused by a fungal pathogen. It is the fungal pathogen that was introduced not the disease itself (the disease does not usually happen in a pathogen's area of origin). Replace by something along the lines of: 'the introduction of the pathogen causing the chestnut blight disease' from Asia to the USA'

Response: We have revised the sentence (Lines 56-59) to accurately state that it was the fungal pathogen causing chestnut blight that was introduced, not the disease itself as follows:

L56-59: "For example, the introduction of the pathogen causing the chestnut blight disease from Asia to the USA, which led to the functional extinction of the American chestnut, killing an estimated four billion trees[18]."

Lines 51-52 The citations are not appropriate, please review them.

Response: We have now replaced them with more relevant and appropriate references in Line 61.

Line 121 Could you state the percentage (as previously done for native forest pest in 32.8% of carbon stock and high-diversity forests)?

Response: We have revised the sentence in Lines 158-162 to state this percentage explicitly:

L158-162: "For pests outside host native ranges, potential risk zones spanned 60.4 Mha of timber-producing areas, 30.1 Mha of forest areas characterized by high forest tree diversity (averaging 52 species per ha), and 27.7 Mha of potential carbon stock (averaging 26 ton per ha), with 39.9% of these potential risk showing co-occurrence of timber-producing and high carbon stock areas (Fig. 2b)."

Line 135 Already stated in the first line of this paragraph

Response: The term "main exposure regions" was already defined in the first sentence of the paragraph. We have now removed the redundant definition from the second.

Lines 187-188 Please, review this sentence. 'While' implies a second clause. And 'most strongest' is not correct.

Response: We have revised the sentence as follows:

L232-234: "Regions with potential carbon stock had the strongest correlation with pathogenic pest richness in the UK (Moran's I = 0.12 and 0.08 for within and outside host native range, respectively; $p < 0.01$) (Fig. 3)."

Line 217 Earlier is written without semicolon. Homogenize throughout the manuscript.

Response: We have now standardized the use of "e.g." throughout the manuscript to "e.g.," (with a comma), which is the preferred format in scientific writing.

Line 230 This figure is lacking in the supplementary material.

Response: We have now revised the corresponding supplementary in Lines 296-298 as below:

L296-298: "These two opposing pathways both shape the distribution of potential risk zones for forest tree diversity, resulting in a nonlinear relationship between precipitation and pest exposure risk (Fig. 4e, f; Supplementary Fig. 9d)."

Lines 288-292 Are a), c), e) and g) representing species richness, and b), d), f) and h) representing the non-native to native ratio? It is not clear in the figure caption.

Response: We have revised the caption to improve clarity as below:

"**Fig. 1 Global spatial pattern of forest pest richness. (a, b)** All forest pests with **(a)** overall richness and **(b)** scaled richness ranging from -1 to +1 comparing the relative difference in pest richness within and outside the native ranges of host trees (positive values indicate higher pest richness within host native ranges). **(c, d)** Fauna**.** **(e, f)** Flora**.** **(g, h)** Pathogenic forest pests. The color bar indicates the species richness of each pixel, with warmer colors representing higher values. To improve display, all maps have been resampled to 1° resolution."

Lines 356-357 This reference only contains a list of species, but lacks their geographic ranges?

Response: To improve clarity, we have revised the paragraph in Lines 469-478 as follows:

L469-478: "To characterize pest distributions, we followed the framework of Gougherty & Davies[90] and distinguished between pests occurring within and outside their host native ranges. This host-centred classification reflects the ecological dependence of forest pests on their hosts, as pest establishment and spread are strongly mediated by host availability and host-pest interactions[91]. We first compiled host associations for 2,038 forest pests and standardized host names using the "taxize" package[92]. Host geographic ranges were then compiled from Global Tree Search[93], the Global Naturalized Alien Flora database[94], CABI[87] and the World Checklist of Vascular Plants[95]. We integrated pest occurrence records with host geographic ranges to determine whether the pest occurred within or outside the native range of its host tree(s).

Overall, we obtained usable geographic range information for 1,875 forest pests."

Lines 357-358 It looks like this list refers to host non-native range, which may be or may not be a native range for the pest. Therefore, this is confusing. Could the authors further explain this? A pest can infect, in its native range, hosts that are in their non-native range, and vice versa.

Response: In our study, the classification is host-centred. Specifically, we categorize pest occurrences based on whether they occur within the native ranges of their host tree species or outside those native host ranges. This approach focuses on the ecological context of the pest-host interaction rather than the biogeographic origin of the pest itself. Under this framework, pest occurrences within host native ranges represent interactions in long-established host-pest systems, whereas occurrences outside host native ranges reflect situations where hosts have been introduced into novel regions and may interact with new pest communities. Importantly, this classification does not require the pest itself to be native or non-native in that region, which avoids the ambiguity highlighted by the reviewer.

To make this clearer, we have rewritten the "Geographic ranges of forest pests" subsection in the Methods to explicitly state that pest occurrences are classified relative to host native ranges. In addition, we have removed the potentially ambiguous terms "native pests" and "non-native pests" throughout the manuscript and replaced them with "pests within host native ranges" and "pests outside host native ranges".

Methods (Lines 469-478):

L469-478: "To characterize pest distributions, we followed the framework of Gougherty & Davies[90] and distinguished between pests occurring within and outside their host native ranges. This host-centred classification reflects the ecological dependence of forest pests on their hosts, as pest establishment and spread are strongly mediated by host availability and host-pest interactions[91]. We first compiled host associations for 2,038 forest pests and standardized host names using the "taxize" package[92]. Host geographic ranges were then compiled from Global Tree Search[93], the

Global Naturalized Alien Flora database[94], CABI[87] and the World Checklist of Vascular Plants[95]. We integrated pest occurrence records with host geographic ranges to determine whether the pest occurred within or outside the native range of its host tree(s). Overall, we obtained usable geographic range information for 1,875 forest pests."

Lines 369-375 This is hugely restrictive as proxy, as timber production is often produced without causing dramatic forest loss. Therefore, how did the authors include all the sustainable timber-producing areas? Why didn't the authors use other sources, such as FAO? Or forest industry reports? Or even complement their approach with timber produced with a certified label?

Response: We agree that timber production does not always lead to detectable forest loss, particularly in sustainably managed forests where harvesting can occur without large-scale canopy removal. However, most alternative data sources suggested by the Reviewer—such as FAO production statistics, forest industry reports, or trade data—are typically reported at national or regional administrative levels and do not provide spatially explicit information on where timber harvesting occurs within countries. Because our analysis requires spatially explicit overlays between pest distributions and timber-producing areas at the grid-cell level, these aggregated datasets cannot be directly integrated into the spatial risk mapping.

For this reason, we used the remote-sensing dataset developed by Curtis et al.[12], which identifies forest disturbances associated with forestry activities using satellite imagery and machine-learning classification. This dataset provides globally consistent spatial coverage at 10 km resolution and has been widely used in global-scale studies to approximate forestry activities[19,20]. In our analysis, grid cells classified as forestry-related disturbances were used as a spatial proxy for timber-producing area.

Following the Reviewer's suggestion, we also explored whether trade-related indicators could help explain pest patterns. Although timber import data cannot be spatially allocated within countries, we incorporated national-level timber import statistics as explanatory variables in the Random Forest driver analysis. This allows us to evaluate whether international timber trade contributes to pest distribution patterns,

while maintaining spatial consistency in the exposure mapping. We have therefore clarified the rationale for using a spatially explicit proxy and including data source of timber import volume in the revised manuscript (Lines 480-489) thus:

L480-489: "To better capture spatial precise distribution of timber production, we employed the data from Curtis et al.[96], which provides a global analysis of forest loss drivers from 2001 to 2015 using satellite imagery and machine learning classification at 10 km resolution. Using 4,985 high-resolution training samples from Google Earth imagery, the dataset used a decision-tree model categorize forest disturbances into five primary drivers, such as commodity-driven deforestation, shifting agriculture, forestry, wildfires, and urbanization. We only included grid cells associated with forestry activities as areas of timber production. To further capture the influence of wood production trade on forest pest exposure, we include timber import volume data which obtained from the Food and Agriculture Organization (FAO) FAOSTAT database[97] in driver analysis."

Line 392 Could you at least provide the list of forest types? It is not clear the sense of that classification and the way it impacts forest vulnerability to pests.

Response: We used the MODIS land cover product (MCD12Q1, IGBP classification scheme), which includes the following forest categories: evergreen needleleaf forest, evergreen broadleaf forest, deciduous needleleaf forest, deciduous broadleaf forest, and mixed forest. Non-forest land cover classes (e.g. grasslands, shrublands, croplands) were excluded from analysis. Different forest types vary in host species composition, climatic conditions, and structural characteristics, which can influence pest host availability and outbreak dynamics[21]. To clarify this point, we have revised the manuscript to briefly describe the forest type classification in Lines 516-521:

L516-521: "Forest type was derived from the Moderate-resolution Imaging Spectroradiometer (MODIS) land cover product (MCD12Q1) which provides 500-m resolution land cover classifications. We filtered the forest grid cell following the global

vegetation classification scheme of International Geosphere Biosphere Programme (IGBP), including evergreen needleleaf, evergreen broadleaf, deciduous needleleaf, deciduous broadleaf and mixed forest."

Line 395 While forest fragmentation is cited in this publication as an important driver of vulnerability to pests, I can't find the link to 'forest type' and 'age'.

Response: The cited reference primarily discusses the role of forest fragmentation in influencing pest vulnerability. In the revised manuscript, we have clarified the sentence so that the citation specifically refers to fragmentation effects. For forest type and stand age, we have now added corresponding citations to support their roles in influencing host composition and susceptibility to pest outbreaks in Lines 514-516 as below:

L514-516: "FFI changes local microclimate and dispersal corridors facilitating pest colonization[12], while forest type and age influence host composition and susceptibility to pest outbreaks[110,111]."

Line 410 "To focus the species occurrence"?

Response: Replaced with "To reduce spatial sampling bias and avoid multiple records representing the same local occurrence" (Lines 532-534). The change clarifies the rationale for retaining only one record per species per grid cell.

Line 411 Correspondingly?

Response: We have revised the text accordingly in Line 535.

Lines 420-423 Suppl. Fig. 5 shows is a risk map, but not Suppl. Fig. 4. Risk is the interaction between a hazard (pest in this case) and the exposure (Fig. 4). So, the latter should be changed to exposure map. And Suppl. Fig. 5 should be called risk map.

Response: We have revised the figure references throughout the manuscript. The risk map is now consistently presented as the overlap between pest richness (hazard) and ecosystem service layers (exposure), as shown in Supplementary Fig. 6 in Lines 563-

567:

L563-567: "To identify potential risk zones, we overlap richness pattern of pests (within and outside host native ranges) and three critical forest ecosystem services, including timber-producing areas, forest diversity and potential carbon stock, respectively. Potential risk zones were defined as grid cells where both pest richness and ecosystem service values fall within the top 50% of their respective distributions (Supplementary Fig. 6)."

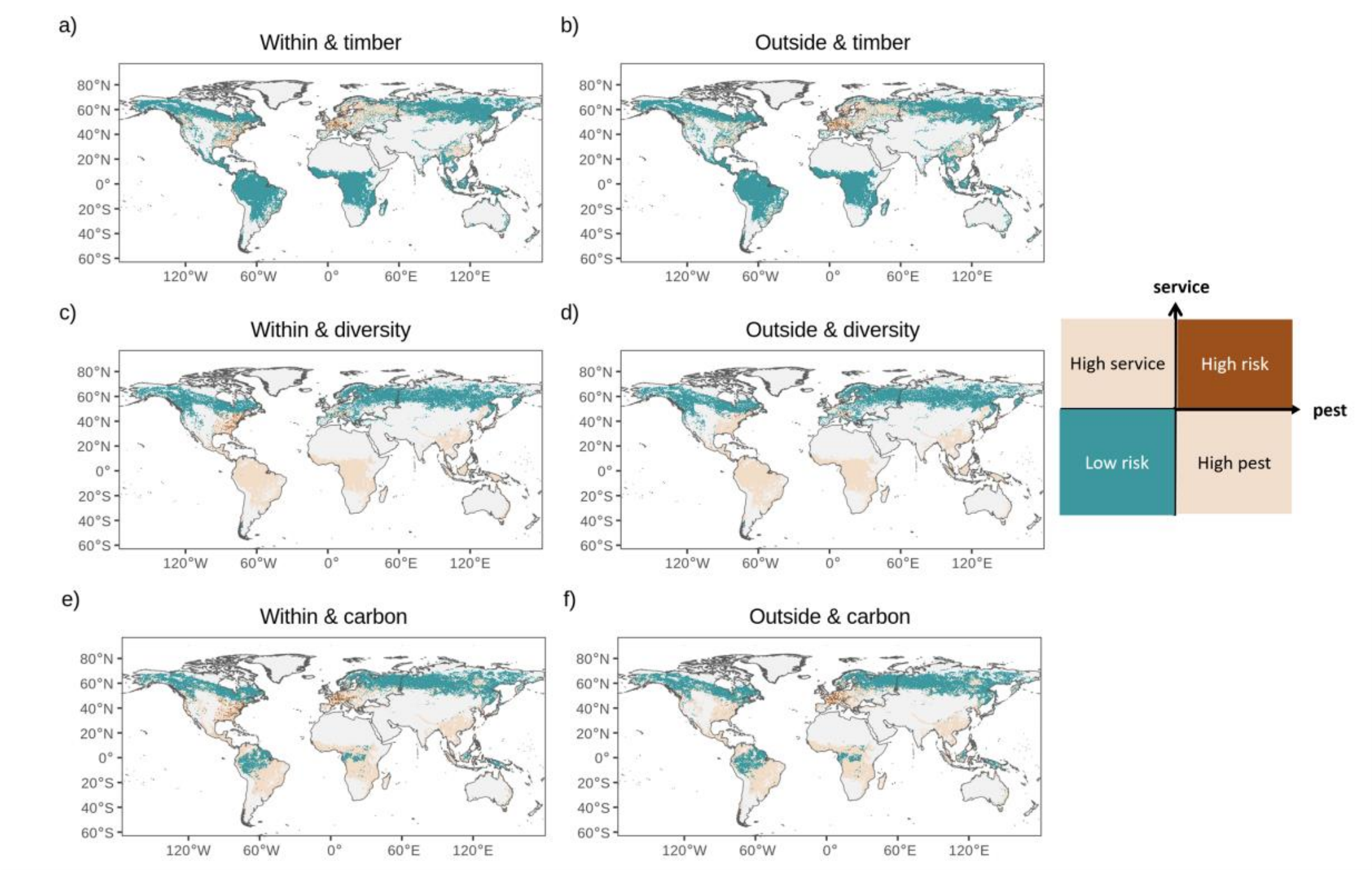

Supplementary Fig. 6 Spatial distribution overlap analysis between different geographic range of forest pests and ecosystem services. Spatial pattern of forests pests within host native ranges between **(a)** timber-producing area, **(c)** forest diversity and **(e)** potential carbon stock. Spatial pattern of forests pests outside host native ranges between **(b)** timber-producing area, **(d)** forest diversity and **(f)** potential carbon stock. The brown color indicated the grid cell where has both high forest pests and ecosystem services, whereas blue indicated the grid cell where has both low forest pests and ecosystem services.

Lines 426-427 in R?

Response: Yes, the analysis was performed in R. We used the “rnaturalearth” package along with other R packages for spatial operations. We have revised the sentence to explicitly state “in R” to avoid any ambiguity in Line 574.

Line 444 ‘is an ensemble-method built-on decision tree’?

Response: We have revised the sentence to: "The random forest algorithm is an ensemble-method built-on decision tree to capture complex nonlinear interactions among predictors, and widely used to process large-scale data with highly-dimensional features."

Line 445 highly-dimensional?

Response: We have revised the text accordingly in Line 603.

Line 453 After running random forest, to apply SHAP? Or for the RF itself? Nine variables are not many for a RF algorithm. Have the authors tested how the RF model errors increase when variables are removed? That might have an impact.

Response: The Random Forest (RF) model was first fitted using the selected predictors, and SHAP values were subsequently applied to interpret the fitted RF model. We have clarified this workflow in the revised manuscript to avoid potential confusion.

Before fitting the RF model, we conducted a variable screening step to reduce strong multicollinearity among predictors. Although Random Forest algorithms are relatively robust to correlated variables, highly correlated predictors ($r > 0.9$) were removed to reduce redundancy and improve the interpretability of variable contributions in the subsequent SHAP analysis. The final model retained nine predictors representing climatic, ecological, and socioeconomic factors (BIO1, BIO3, BIO12, forest age, forest type, gross national income, timber import volume, population density, and travel time). While Random Forest models can accommodate a larger number of predictors, our objective was to retain a parsimonious set of variables representing key hypothesized drivers of pest richness.

Following the Reviewer's suggestion, we additionally evaluated the sensitivity of the Random Forest model to predictor selection by comparing model performance between a model including all candidate predictors and the reduced model containing the nine selected variables. Model performance was nearly identical between the two models, with only negligible differences in spatial cross-validated accuracy (ΔCV accuracy < 0.02; Fig. R1). These results indicate that the reduced predictor set provides

a parsimonious representation of the main drivers without materially affecting predictive performance. Therefore, the nine predictors were retained in the final model.

To further improve clarity, we have also moved the sentence describing the variable filtering step so that it now appears immediately after the description of the Random Forest model and before the introduction of the SHAP analysis (Lines 591–601):

L591–601: "We further used the random forest (RF) machine learning algorithm using the "ranger" package[117], along with Shapley additive explanations (SHAP) using the "fastshap" package[118] to quantify the relative importance of the different factors on high risk zones. The response variable was the presence of potential risk zones identified from the spatial overlap between pest richness and ecosystem services. Predictor variables included environmental, landscape, and socioeconomic factors. Although Random Forests are inherently robust to multicollinearity, we removed several highly correlated climate predictors (*r* > *0.9*) to improve computational efficiency and model interpretability. Nine variables were retained for final model construction, including mean temperature (BIO1), isothermality (BIO3), mean precipitation (BIO12), forest age, forest type, GNI, timber import volume, population density, and travel time."

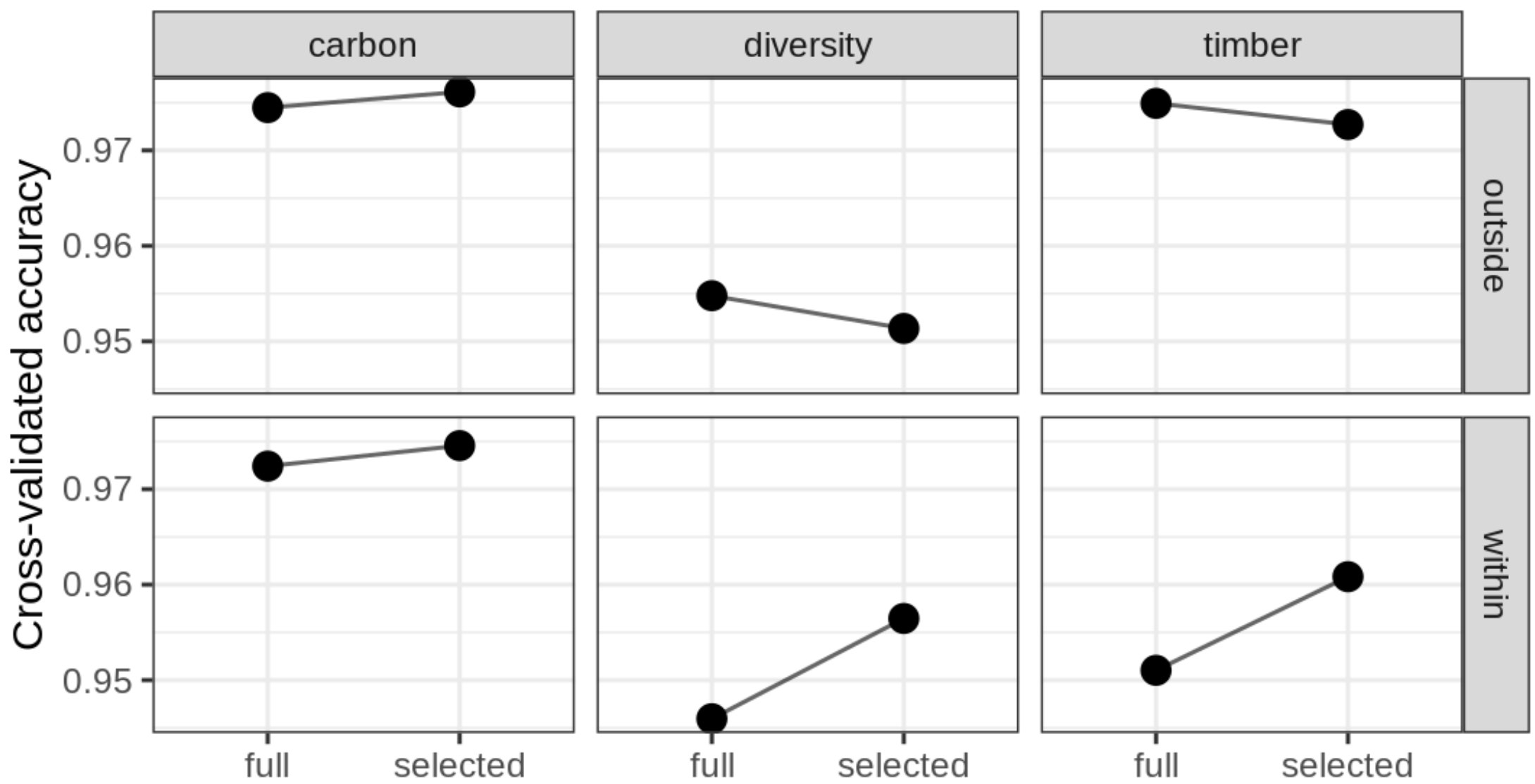

Fig. R1 Sensitivity of Random Forest model performance to predictor selection.

Model performance was compared between a model including all candidate predictors ("full") and a reduced model including the nine selected predictors used in the final analysis ("selected"). Points represent spatial cross-validated accuracy for each dataset, and lines connect results from the same dataset. Differences in model performance between the two models were minimal, indicating that removing highly correlated predictors did not materially affect model accuracy.

Line 455 Check grammar please.

Response: We have revised the sentence to: "For each forest ecosystem service with native or non-native range combinations, we adopted spatial cross-validation with three spatial fold partitions in the data to control spatial autocorrelation and avoid overoptimistic performance estimates."

Supplementary Fig1: FAO definition, not FAO defination

Response: We have revised the supplementary Fig.1 accordingly.

References:

1. Fabre, B., Piou, D., Desprez-Loustau, M.-L. & Marçais, B. Can the emergence of pine diplodia shoot blight in France be explained by changes in pathogen pressure linked to climate change? *Global Change Biol.* **17**, 3218–3227 (2011).
2. Lorenz, M. International Co-operative programme on assessment and monitoring of air pollution effects on forests-ICP forests-. *Water, Air, Soil Pollut.* **85**, 1221–1226 (1995).
3. EPPO. EPPO Global Database. (2025).
4. Castro-Souza, R. A. *et al.* Mapping ignorance to uncover shortfalls in the knowledge on global orthoptera distribution. *npj Biodivers.* **3**, 22 (2024).
5. Olivares-Rojas, J. P., Cook, C. N., Duncan, D. H. & Walsh, J. C. A synthesis of threats impacting australian threatened ecological communities. *Divers. Distrib.* **31**, e13929 (2025).
6. Scott, P., Bader, M. K.-F., Burgess, T., Hardy, G. & Williams, N. Global biogeography and invasion risk of the plant pathogen genus phytophthora. *Environ. Sci. Policy* **101**, 175–182 (2019).
7. Janz, N., Nylin, S. & Wahlberg, N. Diversity begets diversity: host expansions and the diversification of plant-feeding insects. *BMC Evol. Biol.* **6**, 4 (2006).

8. Dyer, L. A. *et al.* Host specificity of lepidoptera in tropical and temperate forests. *Nature* **448**, 696–699 (2007).
9. Meyer, C., Kreft, H., Guralnick, R. & Jetz, W. Global priorities for an effective information basis of biodiversity distributions. *Nat. Commun.* **6**, 8221 (2015).
10. Civitello, D. J. *et al.* Biodiversity inhibits parasites: broad evidence for the dilution effect. *Proc. Natl. Acad. Sci.* **112**, 8667–8671 (2015).
11. Field, E., Hector, A., Barsoum, N. & Koricheva, J. Tree diversity reduces pathogen damage in temperate forests: a systematic review and meta-analysis. *For. Ecol. Manage.* **578**, 122398 (2025).
12. Curtis, P. G., Slay, C. M., Harris, N. L., Tyukavina, A. & Hansen, M. C. Classifying drivers of global forest loss. *Science* **361**, 1108–1111 (2018).
13. Reisinger, A. *et al. The Concept of Risk in the IPCC Sixth Assessment Report: A Summary of Cross-Working Group Discussions*. https://www.ipcc.ch/site/assets/uploads/2021/01/Revised_Guidance_Note_on_Risk_2020_Final.pdf (2020).
14. Gougherty, A. V. & Davies, T. J. A global analysis of tree pests and emerging pest threats. *Proc. Natl. Acad. Sci.* **119**, e2113298119 (2022).
15. Wang, S. S., Gougherty, A. V. & Davies, T. J. Non-native tree pests have a broader host range than native pests and differentially impact host lineages. *J. Ecol.* **110**, 2898–2910 (2022).
16. Zou, Y. *et al.* Fragmentation increased in over half of global forests from 2000 to 2020. *Science* **389**, 1151–1156 (2025).
17. Liang, J. *et al.* Co-limitation towards lower latitudes shapes global forest diversity gradients. *Nat. Ecol. Evol.* **6**, 1423–1437 (2022).
18. Michael A. Charles & Duane D. McKenna. Did the Functional Extinction of the American Chestnut, Castanea dentata, Result in the Extinction of the Greater Chestnut Weevil, Curculio caryatrypes? *Northeast. Nat.* **30**, 511–520 (2024).
19. Bousfield, C. G., Lindenmayer, David. B. & Edwards, D. P. Substantial and increasing global losses of timber-producing forest due to wildfires. *Nat. Geosci.* **16**, 1145–1150 (2023).
20. Bousfield, C. G., Morton, O. & Edwards, D. P. Climate change will exacerbate land conflict between agriculture and timber production. *Nat. Clim. Change* **14**, 1071–1077 (2024).
21. Stewart, P. S. *et al.* Patterns and drivers of pest and disease occurrence in UK treescapes. *Global Change Biol.* **32**, e70706 (2026).

########################Point-by-point response letter ######################

Reviewer #1 (Remarks to the Author):

I have read the manuscript and the modifications introduced by the authors, and I acknowledge the great effort they have made to include the suggested changes. The manuscript has remarkably improved in clarity and robustness. However, I still have some concerns in terms of the interpretation of the additional analysis regarding sampling bias.

Response: We thank the Reviewer for their positive assessment of the revised manuscript and for their careful comments on the sampling-bias analyses. We agree that the SRR analyses required clearer interpretation.

In response, we have substantially revised the sampling-bias section. First, we recalculated the SRR following the Reviewer's suggestion, using country-level pest species richness and the remaining forest pest groups as the reference values. Second, we applied a minimum-observation filter to exclude countries with very sparse records. Third, we updated Supplementary Fig. 5 to show country-level raw richness, occurrence-record density and residuals together, allowing the residual model to be interpreted in the context of recording intensity. We have also revised the Results, Methods, and figure captions accordingly to enhance clarity.

My first concern is the way of calculating SRR values. The authors take reference values to be the total cases in GBIF, but reference values should be the raw number of records for a set of problems selected to be typical of the host population of interest (Fabre et al 2011), which is usually taken to be the raw number of records of all the problems detected in the country except for the case study (in that case either fauna or flora or pathogens). That means that the SRR for plants would take as reference cases all the other records except for plants. That leads to the interpretation that SRR > 1 (positive values in log scale) means that the richness is higher than the average, and SRR < 1 (negative values in log scale) means that the richness is lower than the average. I believe that this should not change enormously the computation of SRR made by the

authors, but as they cite Fabre et al 2011, it should be either done that way, or explained why it is done otherwise.

Response: We agree that using all GBIF records as the reference background did not fully match the method of Fabre et al[1]. Although all GBIF records provide a broad estimate of background biodiversity reporting, they may be influenced by highly recorded taxa unrelated to forest pest detection. Following the Reviewer's comment, we have recalculated the SRR using country-level pest species richness. For each country and pest group, observation value was defined as the species richness of that type. Reference value was defined as the summed species richness of the remaining two forest pest groups in the same country. Thus, for flora pests, the reference value was the summed richness of fauna and pathogen forest pests. The country-level SRR was then standardized by log10-transformed.

As shown in the updated Supplementary Fig. 4, the spatial patterns of the scaled SRR better account for whether raw pest-richness patterns were robust to uneven recording intensity. Positive values indicate that a given pest type accounts for a higher richness in a country than expected from the global average, whereas negative values indicate that this pest group accounts for a lower richness than expected. Several western European countries retained positive SRR values for some pest groups. For example, fauna pests showed positive SRR values in France (log10 SRR = 0.08), Germany (0.06), the United Kingdom (0.11), Belgium (0.21), and the Netherlands (0.41), while flora pests showed positive SRR values in France (0.02), Germany (0.02), Spain (0.18), and Italy (0.08). The USA had lower SRR for flora (-0.28), and positive relative richness for fauna (0.05) and pathogens (0.15), while Canada showed lower SRR for fauna (-0.02) and flora (-0.31), and positive pathogen SRR (0.27). Therefore, this pattern derived from scaled SRR showed that western Europe remains more consistently supported as a high-richness region after SRR standardization, whereas high raw richness in eastern North America should be interpreted more cautiously and may partly reflect stronger observation pressure.

We have updated the Results (Lines 124-136), Methods (Lines 553-565), Supplementary Fig.4 accordingly as below:

L124-136: “To assess the robustness of these patterns, we computed scaled standardized report rates (SRR) following Fabre et al.[1]. Our SRR-scaled analysis revealed that several western European countries retained high pest richness after standardization, with Belgium, the UK, France and Germany showing positive scaled SRR values (Supplementary Fig. 4). This pattern was accompanied by high occurrence-record density, consistent with established forest health monitoring networks supported by dense scientific survey and cross-border collaboration in Europe[2,3]. In contrast, the high raw richness observed in eastern North America was less consistently retained after standardization. The USA showed only positive scaled SRR values for fauna (0.05) and pathogen pests (0.15), but a negative value for flora pests (-0.28)(Supplementary Fig. 4). These results indicate that the high raw richness in eastern North America is not consistently retained after SRR standardization, and is likely influenced by high reporting intensity for some pest groups, especially those with negative scaled SRR values.”

L553-565: “To evaluate whether raw pest-richness patterns were robust to uneven recording intensity, we calculated a standardized report rate (SRR) following the framework of Fabre et al.[1]. For each pest type (fauna, flora, and pathogen) and country, observation value was defined as the species richness of that type. Reference value was defined as the summed species richness of the remaining two forest pest groups in the same country. The SRR was calculated as the ratio of observation value to reference value and weighted by the global expected frequency of each pest type (i.e., the proportion of global richness belonging to that pest type relative to other pest groups). The SRR values were log10-transformed, so that positive values indicate that a given pest type accounts for a higher richness in a country than expected from the global average, whereas negative values indicate that this pest group accounts for a lower richness than expected (Supplementary Fig. 4). Because SRR values can be unstable in countries with very few records, we retained SRR values only for countries with at least 10 records for the pest type being assessed before mapping the results.”

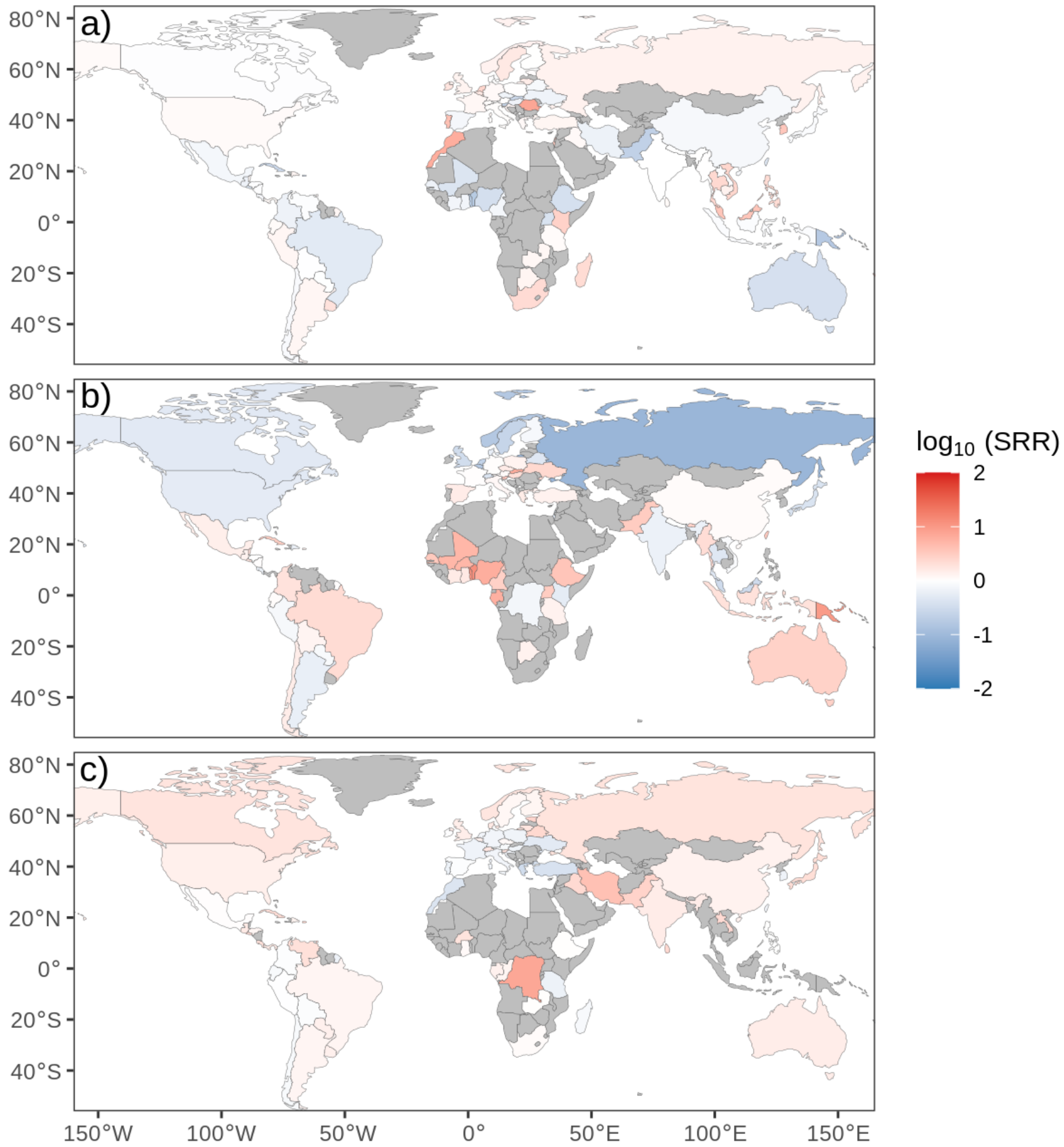

Supplementary Fig. 4 Standardized report rate (SRR) in forest pest richness. Global distribution of SRR for (a) fauna, (b) flora, and (c) pathogen forest pest at the country level. Positive values (red) indicate that a given pest group accounts for a higher forest pest richness in a country than expected from the global average, whereas negative values (blue) indicate lower than expected. Countries with fewer than 10 records for the pest group being assessed were treated as insufficiently sampled and are shown in grey.

Second, the SRR or scaled SRR -which I presume is the log-transformed SRR- is exactly the unbiased value of richness itself (of fauna, flora or pathogen forest pests)

that the authors aimed at evaluating. The scaled SRR itself does not indicate that observation effort varies substantially across regions relative to background sampling effort for forest pest surveillance (cited from rebuttal letter); it is the comparison of SRR with the raw richness data that would indicate this (the dissimilarity with this data). While western Europe it is confirmed to host highly rich fauna, flora and pathogen forest pests, eastern North America is not, because the scaled SRR is too low (the pattern does not hold after correcting for the sampling bias). That means that the high raw richness values observed in eastern North America are due to a higher observation pressure. Western Europe presents both high unbiased richness (approximated through SRR) and high observation pressure. That means that the high observed richness is genuinely high, and not due to a high observation pressure. Therefore, this sentence in the rebuttal letter "United States shows scaled SRR values close to zero (fauna = -0.03, pathogen = -0.02, overall = -0.12), suggesting that pest occurrence records in eastern USA reflect background-level sampling effort rather than strong oversampling" is incorrect. The below-zero log-transformed SRR values (unbiased richness) indicate that actual richness is lower than expected, and the high raw values are explained by high observation pressure. All in all, SRR is an estimation of unbiased richness to be able to be compared to other values across the globe, and should be presented as such. Authors interpretation is then not accurate and can lead to wrong conclusions.

Response: Following the Reviewer's suggestion, we recalculated the SRR using country-level pest species richness and the reference groups described above. We also agree that scaled SRR should be interpreted as a standardized relative-richness metric rather than as a direct measure of observation effort. Therefore, where raw richness is high, but scaled SRR is low or negative, the raw richness pattern is likely to be influenced by high reporting intensity. In western Europe, several countries showed both high raw richness and positive scaled SRR values for more than one pest type. For example, France had high total pest richness and positive scaled SRR values for fauna (0.08) and flora pests (0.02), while Germany showed a similar pattern with positive scaled SRR values for fauna (0.06) and flora pests (0.02). Belgium and the UK also retained positive scaled SRR values for fauna and pathogen pests. These results indicate

that the high raw richness observed in parts of western Europe is retained after SRR standardization and is therefore more robustly supported.

The United States had positive scaled SRR values for fauna (0.05) and pathogen pests (0.15), but a negative value for flora pests (-0.28). Canada had a positive scaled SRR value only for pathogen pests (0.27), but negative values for fauna (-0.02) and flora pests (-0.31). These results indicate that the high raw richness observed in eastern North America should be interpreted cautiously and is likely influenced by high reporting intensity.

The same revised Results, Methods and Supplementary Fig. 4 caption are provided above.

Finally, the Sahel areas as well as Greenland support remarkably high values of SRR (as guessed by the scaled values). Those are most likely an artefact of the SRR computation. This standardization works well when there is a minimal density of observations. There is no established threshold, but I would say under 10 observed values (as a very minimal value) it would not be correct to use it. Observations are almost absent from those areas in Supplementary Figure 3. One solution would be to group a few countries together into a bigger region for SRR calculation. Otherwise they should be excluded from the analyses, along with the explanation of very low observation density.

Response: We agree with the Reviewer that SRR estimates can become unstable in countries with very low numbers of observations. Because SRR is a ratio-based metric, small focal or reference values can generate extreme scaled SRR values that are unlikely to represent reliable standardized richness. Following the Reviewer's suggestion, we therefore applied a minimum-observation filter to the recalculated SRR analysis. Specifically, country-pest group combinations with fewer than 10 observed records were treated as insufficiently sampled and excluded from the SRR maps and interpretation.

We have updated the Method (Lines 563-565) and Supplementary Fig. 4 accordingly as below:

L563-565: “Because SRR values can be unstable in countries with very few records, we retained SRR values only for countries with at least 10 records for the pest type being assessed before mapping the results.”

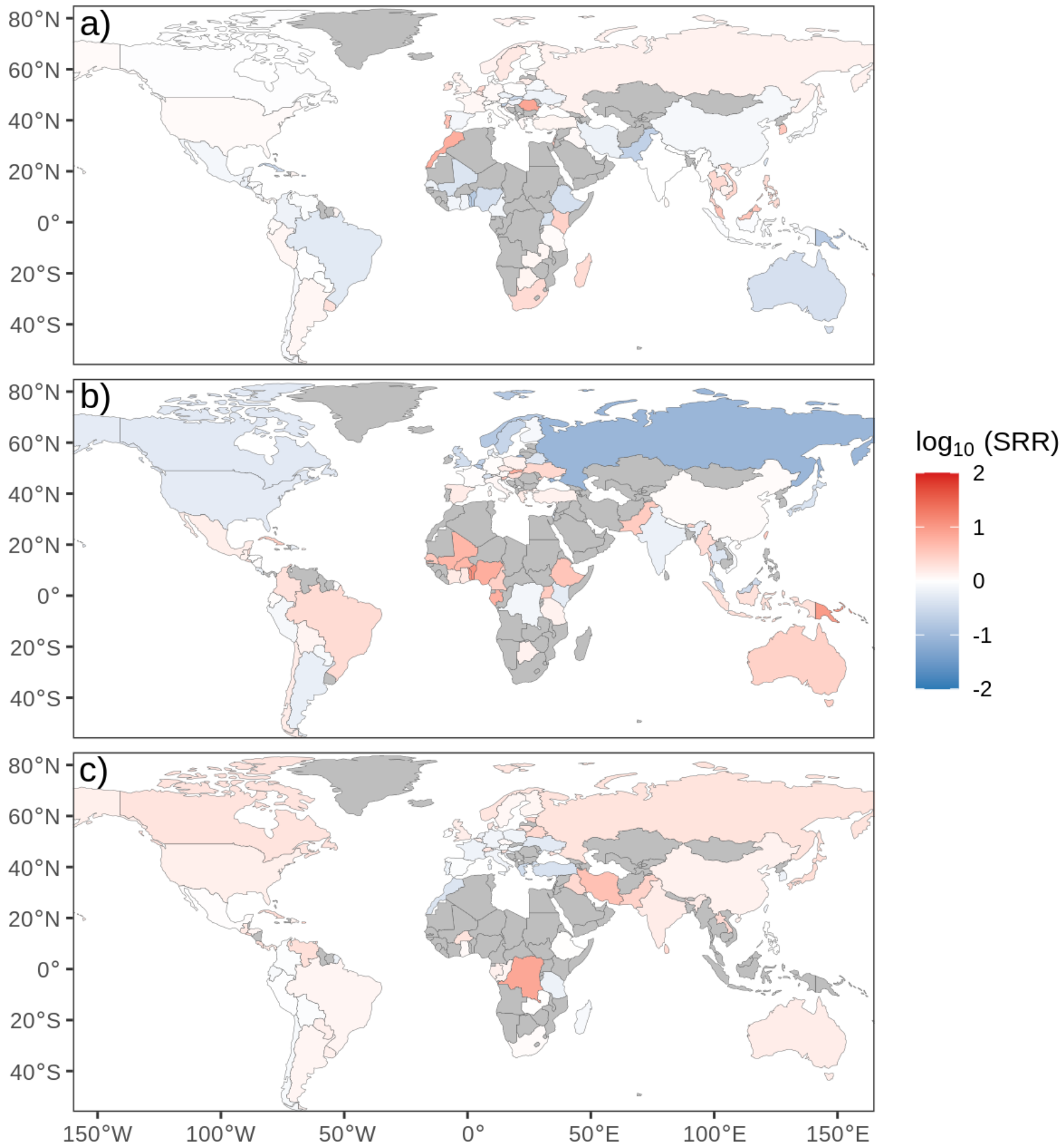

Supplementary Fig. 4 Standardized report rate (SRR) in forest pest richness. Global distribution of SRR for (a) fauna, (b) flora, and (c) pathogen forest pest at the country level. Positive values (red) indicate that a given pest group accounts for a higher forest pest richness in a country than expected from the global average, whereas negative values (blue) indicate lower than expected. Countries with fewer than 10 records for the pest group being assessed were treated as insufficiently

sampled and are shown in grey.

With regard to the second approach (GNI, imports and forest area), I see it as a complementary analysis to validate SRR data, the latter being the main output of the study. However, while residuals are informative, it would be even more informative to visualize the sampling effort of the countries itself showing all GBIF records per country (or whatever has been used to calculate SRR values), along with those residuals. Otherwise, it is difficult to imagine whether the residuals are due to socio-economic or ecological drivers. I find the interpretation difficult to reconcile and assign to an underlying cause.

Response: We agree that the residual analysis should be interpreted as a complementary diagnostic analysis and that its interpretation is clearer when shown together with country-level observation density. To address this, we have updated supplementary figure 5 showing three country-level analysis. First, we show total forest pest species richness per country, which is the response variable used in the residual model and also the richness basis for the recalculated SRR analysis. Second, we show the total number of forest pest occurrence records per country, as an indicator of country-level recording intensity. Third, we show the standardized residuals from the model relating country-level forest pest richness to GNI, timber import volume and forest area.

This updated figure allows direct comparison among raw richness, recording intensity and model residuals. For example, countries with high recording intensity, such as France, Spain, Australia, the United Kingdom and the Netherlands, also showed positive standardized residuals, indicating that their observed forest-pest richness remained higher than expected after accounting for GNI, forest area and timber imports. This pattern is consistent with the recalculated SRR results for parts of western Europe, where high raw richness was also retained after standardization. By contrast, eastern North America showed high raw richness and high occurrence records, but this pattern was less consistently retained across the standardized analyses. The United States had a positive standardized residual (0.57), and its scaled SRR values were positive for

fauna and pathogen pests but negative for flora pests. Canada had high occurrence records but a negative standardized residual (-1.15), with positive scaled SRR only for pathogen pests. These results indicate that high raw richness in eastern North America does not consistently correspond to higher-than-expected richness after accounting for country-level predictors, nor to positive scaled SRR values across all pest groups. In tropical regions, many countries had low occurrence records or insufficient data for reliable SRR estimation.

We have updated the Results (Lines 136-143), Method (Lines 566-568) and Supplementary Fig. 5 accordingly as below:

L136-143: "To support the interpretation of the SRR analysis, we constructed a null model incorporating gross national income (GNI), forest area, and timber import volume (R^2 = 0.718, $p < 0.01$). After accounting for these predictors, northwestern European countries still exhibited positive residuals (Supplementary Fig. 5), indicating that observed pest richness in these regions remained higher than expected given these predictors. Together with high occurrence records and positive scaled SRR, this suggests that high raw richness in western Europe was robust after standardization."

L566-568: "To support the interpretation of the SRR analysis, we further constructed a linear model relating pest richness to economic capacity, trade pressure, and host resource availability following the approach of Scott et al.[4]."

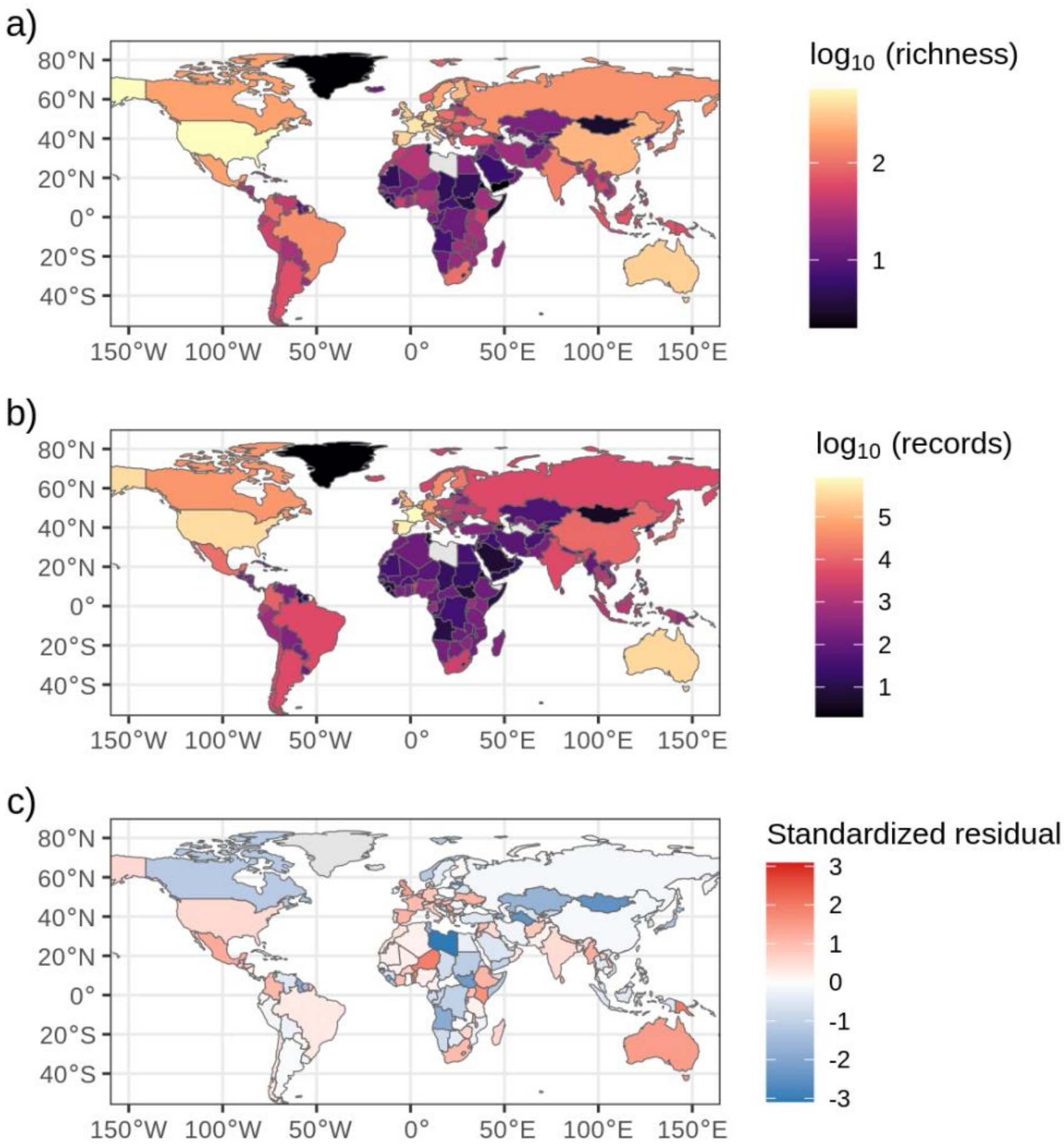

Supplementary Fig. 5 Country-level forest-pest richness, observation density and residual richness patterns. (a) Total forest pest species richness per country, used as the response variable in the residual model. **(b)** Total number of forest pest occurrence records per country, used as an indicator of country-level recording intensity. In **a** and **b**, colours represent log10-transformed richness and records, respectively, with darker colours indicating lower values and lighter yellow colours indicating higher values. **(c)** Standardized residuals from the model relating country-level forest pest richness to gross national income, timber import volume and forest area. Positive (red) value indicates countries where observed richness was higher than predicted by the model, whereas negative (blue) value indicates countries where observed richness was lower than predicted. Grey indicates countries with missing covariate data or insufficient

information that were not included in the residual model.

Figure 5 should state what grey color corresponds to.

Response: We thank the Reviewer for noting this. We have revised the caption to clarify the meaning of the grey colour in Supplementary Fig. 5 as above. Grey now indicates countries with insufficient data or missing covariate information that were not included in the corresponding analysis.

Regarding the relationship between forest pest richness and forest diversity, I agree with the authors justification. However, it is not supported by the SRR data, which shows that tropical regions in America have not a high pest diversity, while a few countries in tropical Africa do. But this inconsistency can be understood by the fact that the characteristics of hyper-diverse forests may need an even higher sampling effort to detect the real pest diversity level in tropical areas. I suggest that the authors adapt their statements to the right interpretation of the SRR.

Response: We agree that the SRR results should be interpreted cautiously across tropical regions because pest observations remain limited and uneven in these areas. SRR values were not consistently high across tropical regions, but this may reflect the greater sampling effort needed to detect low density pests in hyper-diverse forests.

Accordingly, we have revised the manuscript in Lines 143-154 as below:

L143-145: "In tropical regions, low raw pest richness was often accompanied by low occurrence records or insufficient data for reliable SRR estimation, indicating that limited recording may constrain the assessment of forest pest richness."

The other remarks have been carefully and correctly addressed, and I congratulate the authors for that effort.

Response: We thank the Reviewer for this positive assessment and for their

constructive suggestions, which have helped us improve the manuscript.

Reviewer #2 (Remarks to the Author):

We thank the authors for their careful revision of their manuscript and for ensuring their results are fully reproducible. We are satisfied with the authors' responses to our queries and their revised version of the text. We would recommend acceptance of the paper at this stage.

Response: We appreciate Reviewer's recognition of the revisions made to improve the clarity and reproducibility of the study, and we are grateful that our responses have addressed their concerns.

Minor comments:

Should line 22 of fig1.R refer to "./data" instead of "pests/forest_pests/data"?

Response: We thank the Reviewer for carefully checking the reproducibility of the code. We have rechecked the remaining scripts to ensure that file paths are relative to the repository structure and can be run after downloading the code and data following the README instructions.

The ReadMe file was a great addition to GitHub. However, some packages used in the scripts are missing from the installation section, e.g. data.table.

Response: We thank the Reviewer for carefully checking the GitHub repository and for pointing out this omission. We have now rechecked all R scripts and updated the README to include the missing dependencies.

Reviewer #2 (Remarks on code availability):

We thank the authors for ensuring their work is fully reproducible.

Response: We thank the Reviewer for their positive assessment of our revised code and data availability materials.

Reviewer #3 (Remarks to the Author):

I co-reviewed this manuscript with one of the reviewers who provided the listed reports. This is part of the Nature Communications initiative to facilitate training in peer review and to provide appropriate recognition for Early Career Researchers who co-review manuscripts.

References:

1. Fabre, B., Piou, D., Desprez-Loustau, M.-L. & Marçais, B. Can the emergence of pine diplodia shoot blight in France be explained by changes in pathogen pressure linked to climate change? *Global Change Biol.* **17**, 3218–3227 (2011).
2. Lorenz, M. International Co-operative programme on assessment and monitoring of air pollution effects on forests-ICP forests-. *Water, Air, Soil Pollut.* **85**, 1221–1226 (1995).
3. EPPO. EPPO Global Database. (2025).
4. Scott, P., Bader, M. K.-F., Burgess, T., Hardy, G. & Williams, N. Global biogeography and invasion risk of the plant pathogen genus phytophthora. *Environ. Sci. Policy* **101**, 175–182 (2019).

########################Point-by-point response letter ######################

Reviewer #1 (Remarks to the Author):

I have carefully revised the changes introduced in the last version of the manuscript following previous comments and recommendations. I am now fully assured of the robustness of the analyses and the conclusions derived from it. I congratulate the authors for this effort and for the remarkable contribution that this paper does in terms of identification of global risk areas to forest pests. The only very minor suggestion I have to this version is that the authors change "SRR standardization" by " the standardization of forest pest records", as SRR contains already the notion of standardization and is thus a repetition.

Response: We thank the reviewer for the positive assessment and helpful suggestion. We have replaced “SRR standardization” with “the standardization of forest pest records” in the revised manuscript.
